# Systematic misestimation of machine learning performance in neuroimaging studies of depression

**DOI:** 10.1038/s41386-021-01020-7

**Published:** 2021-05-06

**Authors:** Claas Flint, Micah Cearns, Nils Opel, Ronny Redlich, David M. A. Mehler, Daniel Emden, Nils R. Winter, Ramona Leenings, Simon B. Eickhoff, Tilo Kircher, Axel Krug, Igor Nenadic, Volker Arolt, Scott Clark, Bernhard T. Baune, Xiaoyi Jiang, Udo Dannlowski, Tim Hahn

**Affiliations:** 1 Department of Psychiatry, University of Münster, Münster, Germany; 2 Faculty of Mathematics and Computer Science, University of Münster, Münster, Germany; 3 Discipline of Psychiatry, School of Medicine, University of Adelaide, Adelaide, SA Australia; 4 Department of Psychiatry, Melbourne Medical School, The University of Melbourne, Parkville, VIC Australia; 5 Institute of Neuroscience and Medicine (INM-7) Research Center Jülich, Jülich, Germany; 6 Institute of Systems Neuroscience, Medical Faculty, Heinrich Heine University Düsseldorf, Düsseldorf, Germany; 7 Department of Psychiatry and Psychotherapy, University of Marburg, Marburg, Germany; 8 The Florey Institute of Neuroscience and Mental Health, The University of Melbourne, Parkville, VIC Australia

**Keywords:** Translational research, Diagnostic markers

## Abstract

We currently observe a disconcerting phenomenon in machine learning studies in psychiatry: While we would expect larger samples to yield better results due to the availability of more data, larger machine learning studies consistently show much weaker performance than the numerous small-scale studies. Here, we systematically investigated this effect focusing on one of the most heavily studied questions in the field, namely the classification of patients suffering from Major Depressive Disorder (MDD) and healthy controls based on neuroimaging data. Drawing upon structural MRI data from a balanced sample of *N* = 1868 MDD patients and healthy controls from our recent international Predictive Analytics Competition (PAC), we first trained and tested a classification model on the full dataset which yielded an accuracy of 61%. Next, we mimicked the process by which researchers would draw samples of various sizes (*N* = 4 to *N* = 150) from the population and showed a strong risk of misestimation. Specifically, for small sample sizes (*N* = 20), we observe accuracies of up to 95%. For medium sample sizes (*N* = 100) accuracies up to 75% were found. Importantly, further investigation showed that sufficiently large test sets effectively protect against performance misestimation whereas larger datasets per se do not. While these results question the validity of a substantial part of the current literature, we outline the relatively low-cost remedy of larger test sets, which is readily available in most cases.

## Introduction

In psychiatry, we are witnessing an explosion of interest in machine learning (ML) and artificial intelligence for prediction and biomarker discovery, paralleling similar developments in personalized medicine [1–4]. In contrast to the majority of investigations employing classic group-level statistical inference, ML approaches aim to build models which allow for individual (i.e., single subject) predictions, thus enabling direct assessment of individual differences and clinical utility [5]. While this constitutes a major advancement for clinical translation, recent results of large-scale investigations have given rise to a fundamental concern in the field: Specifically, machine learning studies including larger samples did not yield stronger performance, but consistently showed weaker results than studies drawing on small samples, calling into question the validity and generalizability of a large number of highly published proof-of-concept studies.

The magnitude of this issue was impressively illustrated by the results of the Predictive Analytics Competition (PAC 2018; Supplementary appendix B) in which participants developed machine learning models to classify healthy controls (HC) and depressive patients (MDD) based on structural MRI data from *N* = 2,240 participants. Despite the best efforts of ~170 machine learners in 49 teams from around the world, performance ranged between 60 and 65% accuracy in a large, independent test set. This is in strong contrast to the numerous smaller studies showing accuracies of 80% or more [6–8].

Further empirical studies focusing on other disorders support this observed effect of performance deterioration with increasing sample size: In a large-scale investigation, Neuhaus & Popescu [9] aggregated original studies across disorder categories, including schizophrenia (total observation *N* = 5563), MDD (*N* = 2042), and attention deficit hyperactivity disorder (ADHD, *N* = 8084), finding an inverse relationship between sample size and balanced accuracy (schizophrenia, *r* = −0.34; MDD, *r* = −0.32; and ADHD, *r* = −0.43). Similar results were observed in a recent review of 200 neuroimaging classification studies of brain disorders, finding a general trend towards lower reported accuracy scores in larger samples [10]. Given that model performance would be expected to increase with more data, these results hint at a fundamental issue hampering current predictive biomarker studies in psychiatry.

From a methodological point of view, it has been known since the early 90’s that training samples should be large when there is a high number of features (i.e., measured variables) and a complex classification rule being fit to a dataset [11]. Recent works have further reiterated this point [12]. Moreover, these effects may have been further amplified by certain cross-validation schemes. For example, Kambeitz et al. [13] observed higher accuracy estimates in studies using hold-out cross-validation strategies compared to 10-fold and leave-one-out (LOOCV), whilst Varoquaux et al. observed that LOOCV leads to unstable and biased estimates, concluding that repeated random splits should be preferred [14].

Although these findings have sparked in-depth conceptual considerations [15], empirical investigations of this problem have been limited to specific cross-validation schemes and small test set sizes [16]. Here, we aim to systematically investigate the effects of both train and test set sample sizes on machine learning model performance in neuroimaging based MDD classification. In addition, as it is possible that effects of systematic misestimation have arisen due to suboptimal pipeline configurations (i.e., the disproportionate use of LOOCV and linear support-vector machines on samples containing more predictors than observations), we also test a further 48 different pipeline configurations to quantify the influence of these additional factors. To demonstrate that this effect was not dependent on the data or any pipeline configurations used in our analyses, we repeat the process using a dummy classifier. To quantify the magnitude of these effects in each configuration, we drew samples of various sizes from the PAC dataset—mimicking the process by which researchers would draw samples from the population of ML studies reported in the literature. The resulting probability distributions are investigated.

## Materials and methods

To investigate the effects of both train and test set sample sizes on machine learning model performance in neuroimaging based MDD classification, we repeatedly drew samples of different sizes from the PAC dataset to imitate the procedure reported in the literature. Subsequently, the resulting probability distributions are investigated.

### Dataset description

The PAC dataset comprised anonymized, pre-processed MRI data of *N* = 2240 individuals obtained from two large, independent, ongoing studies—the Münster Neuroimaging Cohort [17, 18] (*N* = 724 MDD; *N* = 285 HC) and the FOR2107-study [19] (*N* = 582 MDD, *N* = 649 HC). Case/control status was diagnosed with the SCID-IV [20] interview employed by trained clinical raters in both studies. In both cohorts, exclusion criteria were any neurological or severe medical condition, MRI contraindications, substance-related disorders, Benzodiazepine treatment and head injuries. For healthy controls, any current or previous psychiatric disorder or use of any psychotropic substances. The Münster Neuroimaging Cohort was scanned at one single MRI-scanner with the same sequence, while the FOR2107-study was scanned at two independent sites [21], yielding 3 different scanner types/sequences. The structural T1-weighted magnetic resonance imaging (MRI) scans were pre-processed with the CAT12 toolbox (http://www.neuro.uni-jena.de/cat, r1184) using default parameters to obtain modulated, normalized grey matter segments (resolution 1.5 × 1.5 × 1.5 mm^3^) which were used for the present analysis. Furthermore, age, gender, scanner type, and total intracranial volume (TIV) were provided.

The FOR2107 cohort project was approved by the Ethics Committees of the Medical Faculties, University of Marburg and University of Münster.

### Machine learning pipeline

To ensure the unbiased approximation of the model’s performance in previously unseen patients (i.e., model generalization), we trained and tested all models in a pipeline to prevent information leaking between patients used for training and validation. To avoid a confounding effect due to an imbalance in the sample, we used random under-sampling in a first step to obtain a balanced sample of 934 MDD cases and 934 healthy controls (HC) (see Supplementary appendix A for summary statistics). To reduce the computational effort, all images have been scaled down to a voxel size of 3 × 3 × 3 mm^3^. Following, every image was converted to a vector, where every voxel served as a feature. After background elimination (features with no variance), 58,255 features remained. For standardization, the features were scaled to have zero mean and unit variance. Finally, a linear support-vector machine (SVM) with default parameters (Scikit-learn [22], v0.20) was trained and model performance was calculated and analysed in the subsequent analyses. The effect of varying scanner-distributions across samples was found to be negligible (see Supplementary appendix D).

### Overall sample size effects

To first examine the effects of overall-set size, we randomly sampled the PAC dataset in steps of 1 from *N* = 4 to 150. For each N value, we created 1000 balanced samples. This yielded 147,000 random samples with equal numbers of patients and healthy controls. Applying the SVM pipeline described above to each of these samples independently allowed us to obtain a distribution of accuracy scores for each N value, respectively. On each sample, one SVM was trained with default parameters using LOOCV. Thus, we trained a total of 11,315,000 SVMs. Since the computational effort increases quadratically with increasing sample size, and in addition, the most recent neuroimaging ML studies using LOOCV rarely exceed *N* = 150, we decided to stop at this value. Following, we evaluated the distribution of accuracy scores estimated for each sample size (*N* = 4–150).

### Training set size effects

Second, to examine the effects of training set size, we varied the size of each training set and then tested performance on a fixed hold-out set. Specifically, we randomly sampled the PAC dataset in steps of 1 from *N* = 4–150 as was done in the previous analysis. For each N value, we created 1000 balanced samples and used each of them to train different models. To then test the effects of training set size on test set performance, we tested each trained model on a balanced test set of *N* = 300. From the resulting distributions, we quantified the probability of overestimating accuracy as a function of training set size.

### Test set size effects

**Fig. 1:**
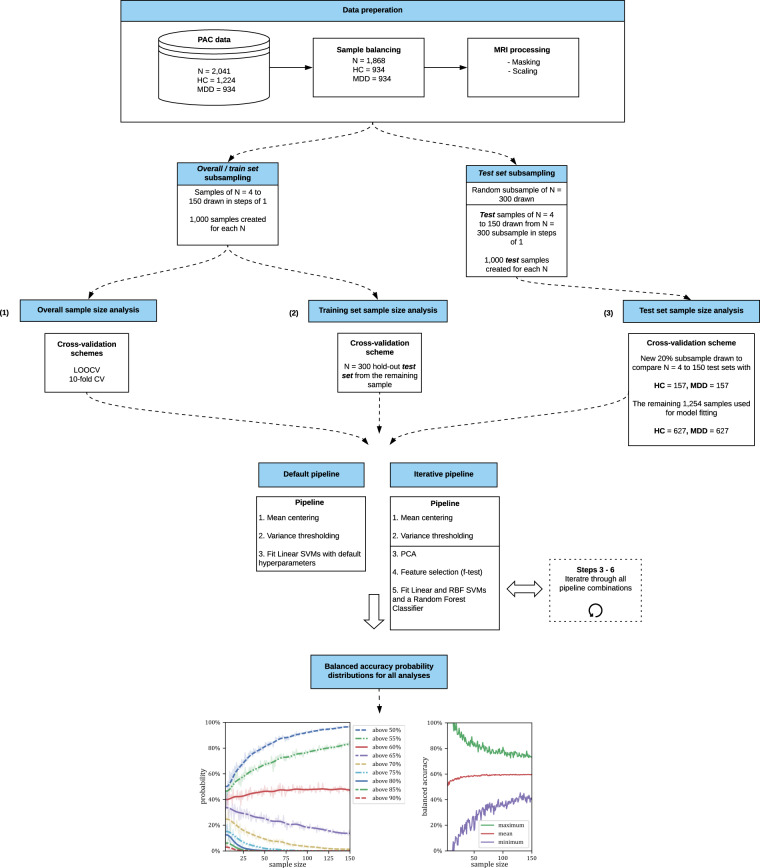
Workflow to investigate the correlations between sample size and misestimation. First the effect of misestimation is investigated over the whole classification process (**(1)** Overall sample size analysis). Following the process of training and testing is evaluated seperatly (**(2)** Training set sample size analysis, **(3)** Test set sample size analysis).

To assess the effects of test set size on classification accuracy, we randomly sampled a balanced group of 300 subjects from our full sample of 934 MDD cases and 934 HC’s. From this sample, we randomly sub-sampled *test* sets of *N* = 4 to 150 in steps of 1. For each value of N, we took 1000 samples, resulting in the creation of 147,000 random *test* samples. From the remaining 784 MDD cases and 784 HC’s, we took out another 20% sample (MDD = 157 and HC = 157). The prediction of this sample provides a reliable basis for overall performance estimation and allows for a comparison with the results obtained from smaller samples. We then trained a single SVM on the remaining sample (MDD = 627, HC = 627). The trained SVM was then tested on each test set sample (*N* = 4–150). From the resulting *test* set accuracy distributions, we derived the probability of obtaining accuracy scores between 50 and 90% accuracy by chance. See Fig. 1 for an overview of all analyses.

### Generalizability of statistical effect

#### Alternative pipeline configurations

As we have attempted to hold other components of the modelling process constant so any observed effects of systematic misestimation can be attributed to sample size alone, it is also possible that our results may be dependent on the basic configuration of our machine learning pipeline (e.g., the use of a linear SVM, default hyperparameters, and LOOCV). Therefore, we trained a further 48 pipeline configurations, including the use of both linear and radial basis function SVMs and a Random Forest classifier, all of which have demonstrated their efficacy in neuroimaging classification studies [13]. Within these configurations, we conducted dimensionality reduction using principal components analysis as well as f-test based feature selection. This allowed us to assess whether our findings were being confounded by the large number of predictors used in the main analysis. For further information see Supplement Appendix F and for all results see supplementary results F.1, supplementary Figs. F4–F51 and Tables F9–F56.

#### Dummy classifier

In order to show that the observed effects were not specific to the PAC dataset or any of the alternative pipeline configurations in these analyses, we repeated the procedures described above with a dummy classifier. Our dummy classifier assumed a prior probability for MDD vs control classification based on the percentage proportion of each class in the training data (prevalence). As the dataset was balanced with random under-sampling, the prior and subsequent ground truth of the model was equal to exactly 50%. This approach allowed us to compare our distribution of dummy performance estimates derived from our subsample analysis to this ground truth value. Importantly, this approach allows for the quantification of accuracy misestimation as a function of sample size completely independent of any unique characteristics that may be specific to the PAC dataset or our pipeline configurations. Therefore, we can then be sure that any subsequent changes in classifier performance are attributable to sample size alone.

## Results

### Overall sample size effects

**Fig. 2:**
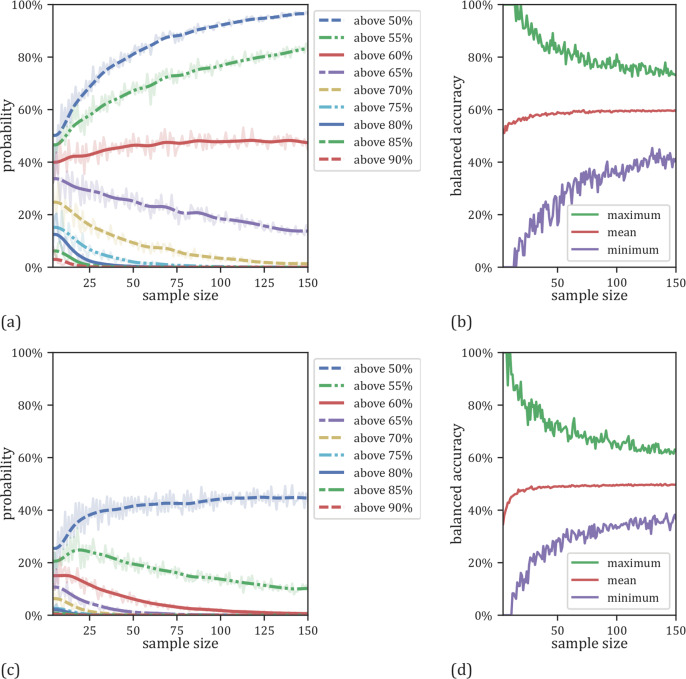
Effects of varying overall sample sizes employing LOOCV. **a** Probabilities for linear SVMs to yield an accuracy exceeding a certain threshold as a function of sample size employing LOOCV. **b** Minimum, maximum and mean results for the linear SVMs as a function of sample size employing LOOCV. **c** Probabilities for dummy classifiers to get an accuracy above a certain chance level related to the size of the used sample. **d** Minimum, maximum and mean results for the dummy classifiers related to the size of the used sample size for training and testing.

In the overall sample size analysis using LOOCV, we were able to show that the risk of overestimating the classifier performance increases with decreasing overall sample size (Fig. 2a, Supplements Table C2a). Specifically, accuracies of 70% or higher are observed with a probability of 13% on sample sizes of *N* = 20 whereas this probability is reduced to 2% for sample sizes of *N* = 100. In addition, the sample size has a profound impact on the variability of accuracy estimates: For samples of size *N* = 20, accuracies ranged from 10 to 95% (standard deviation=15%) while for samples of *N* = 100, accuracies ranged between 35 and 81% (standard deviation = 6%) (Fig. 2b, Supplementary Table C2b). Note that this effect is symmetrical and also applies to the underestimation of performance (Fig. 2b). In addition, the results of the dummy classifier (Fig. 2c, d, Supplementary Table C3) show that the observed overestimation effect is a general effect of sample size as previously pointed out by Varoquaux [16]. As the regularization of the SVM is sensitive to the total number of outliers, which may increase in parallel with sample size, we conducted an additional analysis with adjusted C parameters, with the observed effect remaining constant across these analyses (see Supplementary Fig. E1 and E2).

### Training set size effects

**Fig. 3:**
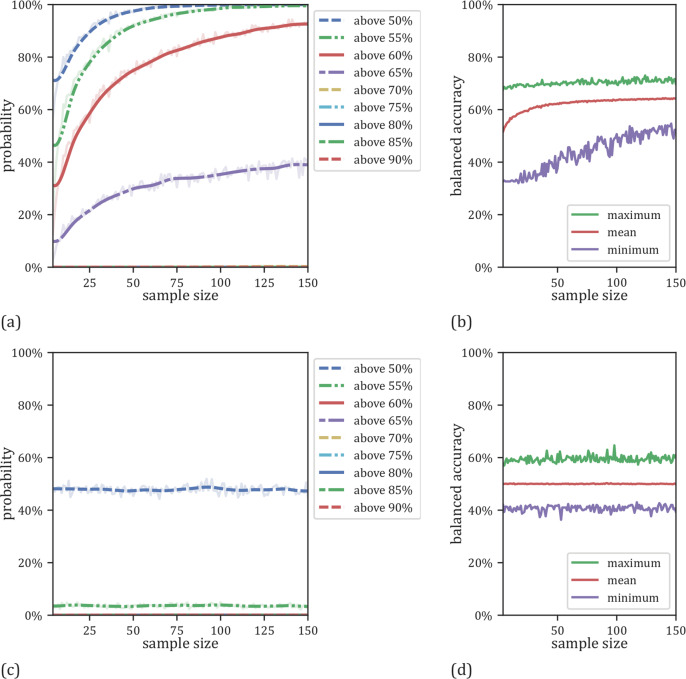
Results as a function of training set sizes with a fixed test set size of *N* = 300. **a** Probabilities for linear SVMs to yield an accuracy exceeding a certain threshold as a function of training sample size. **b** Minimum, maximum and mean results for the linear SVMs as a function of training sample size. **c** Probabilities for the dummy classifier to get an accuracy above a certain chance level related to the size of the training set size. **d** Minimum, maximum and mean results for the dummy classifier related to the size of the used sample size for training.

When examining the effects of training set size (*N* = 4–150) using a large test set for evaluation (*N* = 300), we did not observe any systematic misestimation (Fig. 3a, Supplementary Table C4a). In fact, models trained on virtually any training set size from *N* = 4–150 were sufficient to obtain maximum model performance. However, increasing training sample size decreased the probability of obtaining very low performance estimate. For training sets of size *N* = 20, accuracies ranged from 32 to 69% (standard deviation = 7.1%) while for training sets of N = 100, accuracies ranged between 51 and 70% (standard deviation = 3.0%) (Fig. 3b, Supplementary Table C4b). In accordance with the overall sample size analysis, the results of the dummy classifier (Fig. 3c, d, Supplementary Table C5) showed that this observed effect was general in nature.

### Test set size effects

**Fig. 4:**
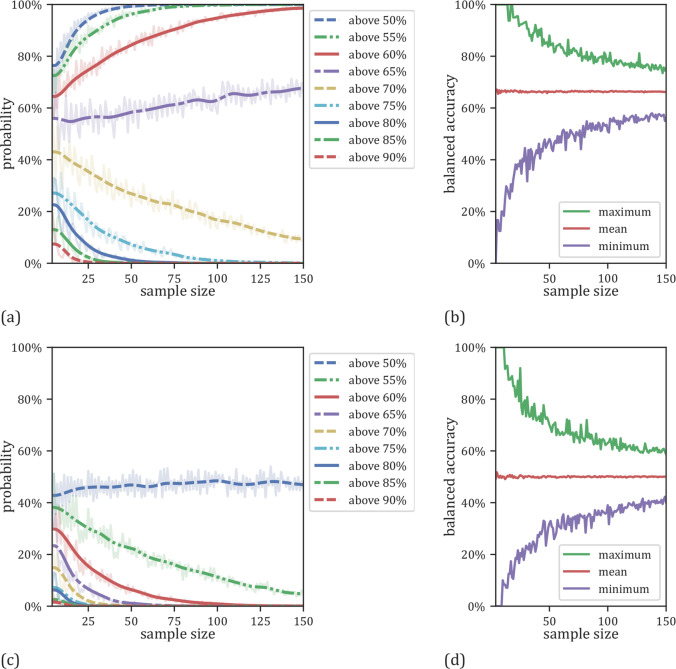
Results as a function of variable test set sizes with and a fixed classifier. **a** Probabilities for linear SVMs to yield an accuracy exceeding a certain threshold as a function of test sample size. **b** Minimum, maximum and mean results for the linear SVMs as a function of test sample size. **c** Probabilities for the dummy classifier to get an accuracy above a certain chance level related to the size of the test set size. **d** Minimum, maximum and mean results for the dummy classifier related to the size of the used sample size for testing.

For our analysis varying test set size, we found a similar pattern of systematic misestimation as that in our first overall sample size analysis using LOOCV. With a sample size of *N* = 20, we obtain results of 70% accuracy or higher with a probability of 30%, whilst the mean accuracy on the full dataset (*N* = 268) was only 61%. This probability dropped to 13% when the test sample size was *N* = 100. For test sets of size *N* = 20, accuracies ranged from 35 to 95% (standard deviation = 10%) whilst for test sets of size *N* = 100, accuracies ranged from 51 and 79% (standard deviation = 4%) (Fig. 4b, Supplementary Table C6b). Running the analysis again using our dummy classifier, we were able to show that the general pattern of systematic misestimation was independent of the specific dataset used (Fig. 4c, d, Supplementary Table C7). To show the independent and generalizable character of the observed effect, we repeated the analysis on the 48 unique pipeline configurations discussed above (see Supplementary appendix F). Specifically, the results are comparable to the originally used configuration, i.e., an SVM with a linear kernel and no preprocessing. Finally, our analysis of scanner sites revealed no effects on model performance (see Supplementary appendix D).

## Discussion

Sparked by the observation that machine learning studies drawing on larger neuroimaging samples consistently showed weaker results than studies drawing on smaller ones, we drew samples of various sizes from the PAC dataset, thereby mimicking the process by which researchers would draw samples from the population of ML studies reported in the literature. When applying a linear SVM with LOOCV, as is the most common approach in the neuroimaging literature [10], we observed a higher risk of misestimations, which may lead to artificially high performance estimates in smaller samples. Importantly, our analyses revealed that this is primarily due to a small test set, not training set size. Generally, this shows that a small test sample size may explain many of the highly optimistic results published in recent years. When considering the well-established effect of publication bias, even if underestimated results are equally as likely, they will have a significantly lower chance of being published.

Our results are the first to disentangle the effects of training and test set size effects which are typically inseparable in common cross-validation frameworks such as LOOCV. This delineation of effects enabled two important insights for biomarker discovery and outcome prediction. First, researchers need to validate their models on large, independent test sets. Our results indicate that in the PAC dataset, a test set size of *N* = 100 was already sufficient to lower the probability of obtaining artificially good performance (i.e., 70% or higher) to 13%. With a median N of less than 100 in many published studies [10], this may seem challenging. However, online infrastructure for independent machine learning model evaluation is available (e.g., www.photon-ai.com/repo). If researchers open-source their models, anyone—independent of technical knowledge or machine learning expertise—can evaluate them. This way, large independent test datasets can be obtained in a short time without the need for data sharing. This is not restricted to neuroimaging data, but any machine learning model. In addition, efforts from consortia will also help to mitigate this problem and should be considered by machine learning practitioners.

Second, the size of the training set alone cannot serve as an indicator of later model performance. Larger training sets are more likely to generalize to new data and broaden model scope (i.e., about which groups within a population a given model can make reasonable predictions), however, in the current analysis, the linear rule learned by an SVM on high-dimensional neuroimaging data could be approximated with only a handful of samples. From a training set size of 30 onward, we no longer observed any increase in model performance. This somewhat counterintuitive effect arises whenever a simple rule is approximated. For higher complexity models (i.e., models capable of learning more complex rules), we, of course, expect performance increases as training sample size increases. However, considering the results of the PAC competition (Supplementary appendix B), high complexity models such as Deep Learning approaches did not yield higher performance when trained with ~1000 samples. Thus, we conclude that simple models are competitive for sample sizes of up to *N* = 1000 for this particular classification problem. Whether more complex rules can be discovered beyond this point or whether other fundamental issues hamper biomarker discovery in psychiatry (cf. e.g., biotype identification [19] and normative modelling approaches [23]) remains an open question.

An intuitive criticism of our main analyses would be that it has merely replicated methods similar to those of previous low-quality works (for example, studies using only linear SVMs with default parameters, tested in LOOCV schemes, on samples with many more predictors than observations). Whilst this pipeline configuration was used in the current analysis to (a) hold constant properties, that if varied, may have been indistinguishably responsible for changes in accuracy misestimation, and (b) replicate the most commonly used ML pipeline configuration in our field, it was important to conduct complementary analyses to rule out these confounds. Therefore, we tested a further 48 ML pipeline configurations (see Supplementary appendix F) using both linear (a linear SVM) and non-linear (a radial basis function SVM and a Random Forest) classifiers. In addition, we conducted PCA based dimensionality reduction as well as f-test based feature selection within these classifiers to delineate whether our findings were confounded by the large size of the predictor space relative to our number of observations. Importantly, all pipeline configurations demonstrated the same pattern of systematic misestimation as that in our main analyses. The second potential criticism of the current work is that these findings may merely be modality-specific, limiting the generalizability of these findings across domains. However, the use of a dummy classifier that completely ignored the input predictor space (the voxels), and instead, classified samples based only on their prevalence in training (MDD = 50%, Control = 50%), showed the same pattern of sample size based systematic misestimation across all pipeline configurations, thus, demonstrating a generalizable statistical effect regardless of the data modality used.

Given the profound effect of the test set size on systematic misestimation, it is important to consider why an effect of overestimation may arise. Previous work by Schnack and Kahn [24] suggests that patient characteristics in smaller samples tend to be more homogenous. In the case of small N, participants may be more likely to be recruited from the same data collection site and of a similar age (for example, in the case of a university recruited convenience sample). In addition, stringent recruitment criterion may be easily met, resulting in a well-defined phenotype that is not truly representative of the parent population of interest. Whilst this explanation makes sense for samples collected in this manner, it fails to explain why we observed this phenomenon in our random sampling procedure, and more importantly, with our dummy classifiers that paid no attention to participants, their characteristics, or the inputted predictor variables. This observation suggests a mechanism for systematic misestimation that is not just sample/patient-specific or contingent on sample homogeneity, but instead, inherent in the natural variation that arises in small test samples. Indeed, this effect is known as sampling error, and as demonstrated by Combrisson et al. [25] can lead to an effect whereby we exceed a machine learning model’s chance level, purely by chance.

In addition to sample size, other issues such as data leakage [13], are likely contributing to the systematic overestimation seen in the literature. Dedicated cross-platform software to help avoid data leakage is freely available (e.g., PHOTON, www.photon-ai.com or Scikit-learn [22]). Finally, code should be made available on request or provided in an online repository (e.g., GitHub or GitLab) upon submission for review. In addition, a more elaborate evaluation framework including the analysis of model scope assessment as well as incremental utility and risk analysis is needed to move the field beyond proof-of-concept studies. The success of current translational efforts in neuroimaging and psychiatry will crucially depend on the timely adoption of guidelines and rules for medical machine learning models (for an in-depth introduction, see [15]).

In summary, our results indicate that—while many of the most highly published results might strongly overestimate true performance—evaluation on large test sets constitutes a straightforward remedy. Given that simple, low-complexity models such as linear SVMs did not gain from larger training set size, researchers should not discard their models due to low training N but seek evaluation on a large test set for any model showing good performance.

### Data access and responsibility

All pIease take responsibility for the integrity of the respective study data and their components. All authors and coauthors had full access to all study data.

## Funding and disclosure

Biomedical financial interests or potential conflicts of interest: TK received unrestricted educational grants from Servier, Janssen, Recordati, Aristo, Otsuka, neuraxpharm. The other authors (CF, MC, NO, RR, DMAM, DE, NRW, RL, SBE, AK, IN, VA, SC, BTB, XJ, UD, TH) declare no conflicts of interest. This work was funded by the German Research Foundation (DFG, grant FOR2107 DA1151/5-1 and DA1151/5-2 to UD; SFB-TRR58, Projects C09 and Z02 to UD) and the Interdisciplinary Centre for Clinical Research (IZKF) of the medical faculty of Münster (grant Dan3/012/17 to UD). TH was supported by the German Research Foundation (DFG grants HA7070/2-2, HA7070/3, HA7070/4). This work is part of the German multicenter consortium “Neurobiology of Affective Disorders. A translational perspective on brain structure and function”, funded by the German Research Foundation (Deutsche Forschungsgemeinschaft DFG; Forschungsgruppe/Research Unit FOR2107). Principal investigators (PIs) with respective areas of responsibility in the FOR2107 consortium are: Work Package WP1, FOR2107/MACS cohort and brain imaging: TK (speaker FOR2107; DFG grant numbers KI 588/14-1, KI 588/14-2), UD (co-speaker FOR2107; DA 1151/5-1, DA 1151/5-2), AK (KR 3822/5-1, KR 3822/7-2), IN (NE 2254/1-2), CK (KO 4291/3-1). WP2, animal phenotyping: Markus Wöhr (WO 1732/4-1, WO 1732/4-2), Rainer Schwarting (SCHW 559/14-1, SCHW 559/14-2). WP3, miRNA: Gerhard Schratt (SCHR 1136/3-1, 1136/3-2). WP4, immunology, mitochondriae: Judith Alferink (AL 1145/5-2), Carsten Culmsee (CU 43/9-1, CU 43/9-2), Holger Garn (GA 545/5-1, GA 545/7-2). WP5, genetics: Marcella Rietschel (RI 908/11-1, RI 908/11-2), Markus Nöthen (NO 246/10-1, NO 246/10-2), Stephanie Witt (WI 3439/3-1, WI 3439/3-2). WP6, multi-method data analytics: Andreas Jansen (JA 1890/7-1, JA 1890/7-2), TH (HA 7070/2-2), Bertram Müller-Myhsok (MU1315/8-2), Astrid Dempfle (DE 1614/3-1, DE 1614/3-2). CP1, biobank: Petra Pfefferle (PF 784/1-1, PF 784/1-2), Harald Renz (RE 737/20-1, 737/20-2). CP2, administration. TK (KI 588/15-1, KI 588/17-1), UD (DA 1151/6-1), Carsten Konrad (KO 4291/4-1). Open Access funding enabled and organized by Projekt DEAL.

## Supplementary information

Supplementary Online Content

## References

[1] Darcy AM, Louie AK, Roberts LW (2016). Machine learning and the profession of medicine. J Am Med Assoc.

[2] Eyre HA, Singh AB, Reynolds C (2016). Tech giants enter mental health. World Psychiatry.

[3] Gabrieli JDE, Ghosh SS, Whitfield-Gabrieli S (2015). Prediction as a humanitarian and pragmatic contribution from human cognitive neuroscience. Neuron..

[4] Jordan MI, Mitchell TM (2015). Machine learning: Trends, perspectives, and prospects. Science..

[5] Hahn T, Nierenberg AA, Whitfield-Gabrieli S (2017). Predictive analytics in mental health: applications, guidelines, challenges and perspectives. Mol Psychiatry..

[6] Johnston BA, Steele JD, Tolomeo S, Christmas D, Matthews K (2015). Structural MRI-based predictions in patients with treatment-refractory depression (TRD). PLoS One..

[7] Mwangi B, Ebmeier KP, Matthews K, Douglas Steele J (2012). Multi-centre diagnostic classification of individual structural neuroimaging scans from patients with major depressive disorder. Brain..

[8] Patel MJ, Andreescu C, Price JC, Edelman KL, Reynolds CF, Aizenstein HJ (2015). Machine learning approaches for integrating clinical and imaging features in late-life depression classification and response prediction. Int J Geriatr Psychiatry..

[9] Neuhaus AH, Popescu FC (2018). Sample Size, Model Robustness, and Classification Accuracy in Diagnostic Multivariate Neuroimaging Analyses. Biol Psychiatry..

[10] Arbabshirani MR, Plis S, Sui J, Calhoun VD (2017). Single subject prediction of brain disorders in neuroimaging: Promises and pitfalls. Neuroimage..

[11] Raudys S, Jain A (1991). Small Sample Size Effects in Statistical Pattern Recognition: Recommendations for Practitioners. IEEE Trans Pattern Anal Mach Intell.

[12] van der Ploeg T, Austin PC, Steyerberg EW (2014). Modern modelling techniques are data hungry: a simulation study for predicting dichotomous endpoints. BMC Med Res Methodol.

[13] Kambeitz J, Cabral C, Sacchet MD, Gotlib IH, Zahn R, Serpa MH (2017). Detecting Neuroimaging Biomarkers for Depression: A Meta-analysis of Multivariate Pattern Recognition Studies. Biol Psychiatry..

[14] Varoquaux G, Raamana PR, Engemann DA, Hoyos-Idrobo A, Schwartz Y, Thirion B (2017). Assessing and tuning brain decoders: cross-validation, caveats, and guidelines. Neuroimage..

[15] Hahn T, Ebner-Priemer U, Meyer-Lindenberg A Transparent Artificial Intelligence – A Conceptual Framework for Evaluating AI-based Clinical Decision Support Systems. OSF Prepr. 2019. 2019. 10.31219/OSF.IO/UZEHJ.

[16] Varoquaux G (2018). Cross-validation failure: small sample sizes lead to large error bars. Neuroimage..

[17] Dannlowski U, Kugel H, Grotegerd D, Redlich R, Suchy J, Opel N (2015). NCAN cross-disorder risk variant is associated with limbic gray matter deficits in healthy subjects and major depression. Neuropsychopharmacology..

[18] Dannlowski U, Grabe HJ, Wittfeld K, Klaus J, Konrad C, Grotegerd D (2015). Multimodal imaging of a tescalcin (TESC)-regulating polymorphism (rs7294919)-specific effects on hippocampal gray matter structure. Mol Psychiatry..

[19] Kircher T, Wöhr M, Nenadic I, Schwarting R, Schratt G, Alferink J, et al. Neurobiology of the major psychoses: a translational perspective on brain structure and function—the FOR2107 consortium. Eur Arch Psychiatry Clin Neurosci. 2018:1–14. 10.1007/s00406-018-0943-x 30267149

[20] Wittchen H-U, Wunderlich U, Gruschwitz S, Zaudig M SKID I. Strukturiertes Klinisches Interview für DSM-IV. Achse I: Psychische Störungen. Interviewheft und Beurteilungsheft. Eine deutschsprachige, erweiterte Bearb. d. amerikanischen Originalversion des SKID I. Göttingen: Hogrefe; 1997.

[21] Vogelbacher C, Möbius TWD, Sommer J, Schuster V, Dannlowski U, Kircher T (2018). The Marburg-Münster Affective Disorders Cohort Study (MACS): A quality assurance protocol for MR neuroimaging data. Neuroimage..

[22] Pedregosa F, Varoquaux G, Gramfort A, Michel V, Thirion B, Grisel O (2012). Scikit-learn: Machine Learning in Python. J Mach Learn Res.

[23] Marquand AF, Rezek I, Buitelaar J, Beckmann CF (2016). Understanding heterogeneity in clinical cohorts using normative models: beyond case-control studies. Biol Psychiatry..

[24] Schnack HG, Kahn RS (2016). Detecting neuroimaging biomarkers for psychiatric disorders: sample size matters. Front Psychiatry.

[25] Combrisson E, Jerbi K (2015). Exceeding chance level by chance: the caveat of theoretical chance levels in brain signal classification and statistical assessment of decoding accuracy. J Neurosci Methods..

