# Supplementary material for: Systematic misestimation of machine learning performance in neuroimaging studies of depression
Source: Neuropsychopharmacology. 2021 May 6;46(8):1510–7. doi: 10.1038/s41386-021-01020-7 (PMC8209109; doi:10.1038/s41386-021-01020-7)
Supplement: Supplementary file 1 — Supplementary Online Content [file 41386_2021_1020_MOESM1_ESM.pdf]

Supplementary Online Content

Systematic Misestimation of Machine Learning Performance in Neuroimaging Studies of Depression

Claas Flint[a,b,1], Micah Cearns[c,e,1], Nils Opel[a], Ronny Redlich[a], David M. A. Mehler[a], Daniel Emden[a], Nils R. Winter[a], Ramona Leenings[a], Simon B. Eickhoff[d,h], Tilo Kircher[f], Axel Krug[f], Igor Nenadic[f], Volker Arolt[a], Scott Clark[c], Bernhard T. Baune[c,e,g], Xiaoyi Jiang[b], Udo Dannlowski[a,2,*], Tim Hahn[a,2]

[a] *Department of Psychiatry, University of Münster, Germany*
[b] *Faculty of Mathematics and Computer Science, University of Münster, Germany*
[c] *Discipline of Psychiatry, School of Medicine, University of Adelaide, Australia*
[d] *Institute of Neuroscience and Medicine (INM-7) Research Center Jülich*
[e] *Department of Psychiatry, Melbourne Medical School, The University of Melbourne, Parkville, Australia*
[f] *Department of Psychiatry and Psychotherapy, University of Marburg, Germany*
[g] *The Florey Institute of Neuroscience and Mental Health, The University of Melbourne, Parkville, Australia*
[h] *Institute of Systems Neuroscience, Medical Faculty, Heinrich Heine University Düsseldorf, Düsseldorf, Germany*

Contents

Appendix A Summary statistics for the final study sample 7

Appendix B Predictive Analytics Competition 2018 7

Appendix C Additional tabular presentations 8

Appendix D Influence of the scanner distribution 15

Appendix E Adjustment of SVM regularization based on sample size 15

Appendix F Alternative Machine Configurations 16
- Appendix F.1 Results . . . 17
  - Appendix F.1.1 Graphical results representation . . . 17
  - Appendix F.1.2 Tabular results representation . . . 42

List of Figures

E.1 Effects of varying overall sample sizes with an adjusted C parameter. . . . 15
E.2 Effects of varying train sample sizes with an adjusted C parameter. . . . 16

*Corresponding author
Email address: dannlow@uni-muenster.de (Udo Dannlowski)
[1]these authors contributed equally to this work and are equal first authors
[2]these authors contributed equally to this work and are equal senior authors

F.3 Effects of varying test sample size. Random Forest; No preprocessing . . 18
F.4 Effects of varying test sample size. SVM (kernel = RBF); No preprocessing 18
F.5 Effects of varying test sample size. Linear SVM; No preprocessing 19
F.6 Effects of varying test sample size. Random Forest; Preprocessing: ANOVA feature selection ($k_{\text{best}} = 10$) 19
F.7 Effects of varying test sample size. SVM (kernel = RBF); Preprocessing: ANOVA feature selection ($k_{\text{best}} = 10$) 20
F.8 Effects of varying test sample size. Linear SVM; Preprocessing: ANOVA feature selection ($k_{\text{best}} = 10$) 20
F.9 Effects of varying test sample size. Random Forest; Preprocessing: ANOVA feature selection ($k_{\text{best}} = 100$) 21
F.10 Effects of varying test sample size. SVM (kernel = RBF); Preprocessing: ANOVA feature selection ($k_{\text{best}} = 100$) 21
F.11 Effects of varying test sample size. Linear SVM; Preprocessing: ANOVA feature selection ($k_{\text{best}} = 100$) 22
F.12 Effects of varying test sample size. Random Forest; Preprocessing: ANOVA feature selection ($k_{\text{best}} = 1{,}000$) 22
F.13 Effects of varying test sample size. SVM (kernel = RBF); Preprocessing: ANOVA feature selection ($k_{\text{best}} = 1{,}000$) 23
F.14 Effects of varying test sample size. Linear SVM; Preprocessing: ANOVA feature selection ($k_{\text{best}} = 1{,}000$) 23
F.15 Effects of varying test sample size. Random Forest; Preprocessing: ANOVA feature selection ($k_{\text{best}} = 10{,}000$) 24
F.16 Effects of varying test sample size. SVM (kernel = RBF); Preprocessing: ANOVA feature selection ($k_{\text{best}} = 10{,}000$) 24
F.17 Effects of varying test sample size. Linear SVM; Preprocessing: ANOVA feature selection ($k_{\text{best}} = 10{,}000$) 25
F.18 Effects of varying test sample size. Random Forest; Preprocessing: ANOVA feature selection ($k_{\text{best}} = 50{,}000$) 25
F.19 Effects of varying test sample size. SVM (kernel = RBF); Preprocessing: ANOVA feature selection ($k_{\text{best}} = 50{,}000$) 26
F.20 Effects of varying test sample size. Linear SVM; Preprocessing: ANOVA feature selection ($k_{\text{best}} = 50{,}000$) 26
F.21 Effects of varying test sample size. Random Forest; Preprocessing: PCA ($n_{\text{components}} = 10$) 27
F.22 Effects of varying test sample size. SVM (kernel = RBF); Preprocessing: PCA ($n_{\text{components}} = 10$) 27
F.23 Effects of varying test sample size. Linear SVM; Preprocessing: PCA ($n_{\text{components}} = 10$) 28
F.24 Effects of varying test sample size. Random Forest; Preprocessing: PCA ($n_{\text{components}} = 50$) 28
F.25 Effects of varying test sample size. SVM (kernel = RBF); Preprocessing: PCA ($n_{\text{components}} = 50$) 29
F.26 Effects of varying test sample size. Linear SVM; Preprocessing: PCA ($n_{\text{components}} = 50$) 29
F.27 Effects of varying test sample size. Random Forest; Preprocessing: PCA ($n_{\text{components}} = 50$); ANOVA feature selection ($k_{\text{best}} = 10$) 30

F.28 Effects of varying test sample size. SVM (kernel = RBF); Preprocessing: PCA ($n_{\text{components}} = 50$); ANOVA feature selection ($k_{\text{best}} = 10$) 30
F.29 Effects of varying test sample size. Linear SVM; Preprocessing: PCA ($n_{\text{components}} = 50$); ANOVA feature selection ($k_{\text{best}} = 10$) 31
F.30 Effects of varying test sample size. Random Forest; Preprocessing: PCA ($n_{\text{components}} = 500$) 31
F.31 Effects of varying test sample size. SVM (kernel = RBF); Preprocessing: PCA ($n_{\text{components}} = 500$) 32
F.32 Effects of varying test sample size. Linear SVM; Preprocessing: PCA ($n_{\text{components}} = 500$) 32
F.33 Effects of varying test sample size. Random Forest; Preprocessing: PCA ($n_{\text{components}} = 500$); ANOVA feature selection ($k_{\text{best}} = 10$) 33
F.34 Effects of varying test sample size. SVM (kernel = RBF); Preprocessing: PCA ($n_{\text{components}} = 500$); ANOVA feature selection ($k_{\text{best}} = 10$) 33
F.35 Effects of varying test sample size. Linear SVM; Preprocessing: PCA ($n_{\text{components}} = 500$); ANOVA feature selection ($k_{\text{best}} = 10$) 34
F.36 Effects of varying test sample size. Random Forest; Preprocessing: PCA ($n_{\text{components}} = 500$); ANOVA feature selection ($k_{\text{best}} = 100$) 34
F.37 Effects of varying test sample size. SVM (kernel = RBF); Preprocessing: PCA ($n_{\text{components}} = 500$); ANOVA feature selection ($k_{\text{best}} = 100$) 35
F.38 Effects of varying test sample size. Linear SVM; Preprocessing: PCA ($n_{\text{components}} = 500$); ANOVA feature selection ($k_{\text{best}} = 100$) 35
F.39 Effects of varying test sample size. Random Forest; Preprocessing: PCA ($n_{\text{components}} = \text{all}$) 36
F.40 Effects of varying test sample size. SVM (kernel = RBF); Preprocessing: PCA ($n_{\text{components}} = \text{all}$) 36
F.41 Effects of varying test sample size. Linear SVM; Preprocessing: PCA ($n_{\text{components}} = \text{all}$) 37
F.42 Effects of varying test sample size. Random Forest; Preprocessing: PCA ($n_{\text{components}} = \text{all}$); ANOVA feature selection ($k_{\text{best}} = 10$) 37
F.43 Effects of varying test sample size. SVM (kernel = RBF); Preprocessing: PCA ($n_{\text{components}} = \text{all}$); ANOVA feature selection ($k_{\text{best}} = 10$) 38
F.44 Effects of varying test sample size. Linear SVM; Preprocessing: PCA ($n_{\text{components}} = \text{all}$); ANOVA feature selection ($k_{\text{best}} = 10$) 38
F.45 Effects of varying test sample size. Random Forest; Preprocessing: PCA ($n_{\text{components}} = \text{all}$); ANOVA feature selection ($k_{\text{best}} = 100$) 39
F.46 Effects of varying test sample size. SVM (kernel = RBF); Preprocessing: PCA ($n_{\text{components}} = \text{all}$); ANOVA feature selection ($k_{\text{best}} = 100$) 39
F.47 Effects of varying test sample size. Linear SVM; Preprocessing: PCA ($n_{\text{components}} = \text{all}$); ANOVA feature selection ($k_{\text{best}} = 100$) 40
F.48 Effects of varying test sample size. Random Forest; Preprocessing: PCA ($n_{\text{components}} = \text{all}$); ANOVA feature selection ($k_{\text{best}} = 1{,}000$) 40
F.49 Effects of varying test sample size. SVM (kernel = RBF); Preprocessing: PCA ($n_{\text{components}} = \text{all}$); ANOVA feature selection ($k_{\text{best}} = 1{,}000$) 41
F.50 Effects of varying test sample size. Linear SVM; Preprocessing: PCA ($n_{\text{components}} = \text{all}$); ANOVA feature selection ($k_{\text{best}} = 1{,}000$) 41

List of Tables

A.1 Summary statistics for the final study sample. 7
B.2 Split between training and test data for the Pac 2018. 8
B.3 Test results on the held-back test set by the top five teams at the Pac 2018. 8
C.4 Varying overall set size with a linear SVM and LOOCV 9
C.5 Varying overall set size with a Dummy Classifier and LOOCV 10
C.6 Varying train set size and fixed validation set on a linear SVM 11
C.7 Varying train set size and fixed validation set on a Dummy Classifier . . 12
C.8 Varying test set size on a fixed pretrained linear SVM 13
C.9 Varying test set size on a fixed pretrained Dummy Classifier 14
F.10 Configurations for the preprocessing 17
F.11 Effects of varying test sample size. Random Forest; No preprocessing . . 43
F.12 Effects of varying test sample size. SVM (kernel = RBF); No preprocessing 44
F.13 Effects of varying test sample size. Linear SVM; No preprocessing 45
F.14 Effects of varying test sample size. Random Forest; Preprocessing: ANOVA feature selection ($k_{\text{best}} = 10$) 46
F.15 Effects of varying test sample size. SVM (kernel = RBF); Preprocessing: ANOVA feature selection ($k_{\text{best}} = 10$) 47
F.16 Effects of varying test sample size. Linear SVM; Preprocessing: ANOVA feature selection ($k_{\text{best}} = 10$) 48
F.17 Effects of varying test sample size. Random Forest; Preprocessing: ANOVA feature selection ($k_{\text{best}} = 100$) 49
F.18 Effects of varying test sample size. SVM (kernel = RBF); Preprocessing: ANOVA feature selection ($k_{\text{best}} = 100$) 50
F.19 Effects of varying test sample size. Linear SVM; Preprocessing: ANOVA feature selection ($k_{\text{best}} = 100$) 51
F.20 Effects of varying test sample size. Random Forest; Preprocessing: ANOVA feature selection ($k_{\text{best}} = 1{,}000$) 52
F.21 Effects of varying test sample size. SVM (kernel = RBF); Preprocessing: ANOVA feature selection ($k_{\text{best}} = 1{,}000$) 53
F.22 Effects of varying test sample size. Linear SVM; Preprocessing: ANOVA feature selection ($k_{\text{best}} = 1{,}000$) 54
F.23 Effects of varying test sample size. Random Forest; Preprocessing: ANOVA feature selection ($k_{\text{best}} = 10{,}000$) 55
F.24 Effects of varying test sample size. SVM (kernel = RBF); Preprocessing: ANOVA feature selection ($k_{\text{best}} = 10{,}000$) 56
F.25 Effects of varying test sample size. Linear SVM; Preprocessing: ANOVA feature selection ($k_{\text{best}} = 10{,}000$) 57
F.26 Effects of varying test sample size. Random Forest; Preprocessing: ANOVA feature selection ($k_{\text{best}} = 50{,}000$) 58
F.27 Effects of varying test sample size. SVM (kernel = RBF); Preprocessing: ANOVA feature selection ($k_{\text{best}} = 50{,}000$) 59
F.28 Effects of varying test sample size. Linear SVM; Preprocessing: ANOVA feature selection ($k_{\text{best}} = 50{,}000$) 60
F.29 Effects of varying test sample size. Random Forest; Preprocessing: PCA ($n_{\text{components}} = 10$) 61
F.30 Effects of varying test sample size. SVM (kernel = RBF); Preprocessing: PCA ($n_{\text{components}} = 10$) 62

F.31 Effects of varying test sample size. Linear SVM; Preprocessing: PCA ($n_{\text{components}} = 10$) 63
F.32 Effects of varying test sample size. Random Forest; Preprocessing: PCA ($n_{\text{components}} = 50$) 64
F.33 Effects of varying test sample size. SVM (kernel = RBF); Preprocessing: PCA ($n_{\text{components}} = 50$) 65
F.34 Effects of varying test sample size. Linear SVM; Preprocessing: PCA ($n_{\text{components}} = 50$) 66
F.35 Effects of varying test sample size. Random Forest; Preprocessing: PCA ($n_{\text{components}} = 50$); ANOVA feature selection ($k_{\text{best}} = 10$) 67
F.36 Effects of varying test sample size. SVM (kernel = RBF); Preprocessing: PCA ($n_{\text{components}} = 50$); ANOVA feature selection ($k_{\text{best}} = 10$) 68
F.37 Effects of varying test sample size. Linear SVM; Preprocessing: PCA ($n_{\text{components}} = 50$); ANOVA feature selection ($k_{\text{best}} = 10$) 69
F.38 Effects of varying test sample size. Random Forest; Preprocessing: PCA ($n_{\text{components}} = 500$) 70
F.39 Effects of varying test sample size. SVM (kernel = RBF); Preprocessing: PCA ($n_{\text{components}} = 500$) 71
F.40 Effects of varying test sample size. Linear SVM; Preprocessing: PCA ($n_{\text{components}} = 500$) 72
F.41 Effects of varying test sample size. Random Forest; Preprocessing: PCA ($n_{\text{components}} = 500$); ANOVA feature selection ($k_{\text{best}} = 10$) 73
F.42 Effects of varying test sample size. SVM (kernel = RBF); Preprocessing: PCA ($n_{\text{components}} = 500$); ANOVA feature selection ($k_{\text{best}} = 10$) 74
F.43 Effects of varying test sample size. Linear SVM; Preprocessing: PCA ($n_{\text{components}} = 500$); ANOVA feature selection ($k_{\text{best}} = 10$) 75
F.44 Effects of varying test sample size. Random Forest; Preprocessing: PCA ($n_{\text{components}} = 500$); ANOVA feature selection ($k_{\text{best}} = 100$) 76
F.45 Effects of varying test sample size. SVM (kernel = RBF); Preprocessing: PCA ($n_{\text{components}} = 500$); ANOVA feature selection ($k_{\text{best}} = 100$) 77
F.46 Effects of varying test sample size. Linear SVM; Preprocessing: PCA ($n_{\text{components}} = 500$); ANOVA feature selection ($k_{\text{best}} = 100$) 78
F.47 Effects of varying test sample size. Random Forest; Preprocessing: PCA ($n_{\text{components}} = \text{all}$) 79
F.48 Effects of varying test sample size. SVM (kernel = RBF); Preprocessing: PCA ($n_{\text{components}} = \text{all}$) 80
F.49 Effects of varying test sample size. Linear SVM; Preprocessing: PCA ($n_{\text{components}} = \text{all}$) 81
F.50 Effects of varying test sample size. Random Forest; Preprocessing: PCA ($n_{\text{components}} = \text{all}$); ANOVA feature selection ($k_{\text{best}} = 10$) 82
F.51 Effects of varying test sample size. SVM (kernel = RBF); Preprocessing: PCA ($n_{\text{components}} = \text{all}$); ANOVA feature selection ($k_{\text{best}} = 10$) 83
F.52 Effects of varying test sample size. Linear SVM; Preprocessing: PCA ($n_{\text{components}} = \text{all}$); ANOVA feature selection ($k_{\text{best}} = 10$) 84
F.53 Effects of varying test sample size. Random Forest; Preprocessing: PCA ($n_{\text{components}} = \text{all}$); ANOVA feature selection ($k_{\text{best}} = 100$) 85
F.54 Effects of varying test sample size. SVM (kernel = RBF); Preprocessing: PCA ($n_{\text{components}} = \text{all}$); ANOVA feature selection ($k_{\text{best}} = 100$) 86

F.55 Effects of varying test sample size. Linear SVM; Preprocessing: PCA ($n_{\text{components}}$ = all); ANOVA feature selection ($k_{\text{best}} = 100$) 87
F.56 Effects of varying test sample size. Random Forest; Preprocessing: PCA ($n_{\text{components}}$ = all); ANOVA feature selection ($k_{\text{best}} = 1{,}000$) 88
F.57 Effects of varying test sample size. SVM (kernel = RBF); Preprocessing: PCA ($n_{\text{components}}$ = all); ANOVA feature selection ($k_{\text{best}} = 1{,}000$) 89
F.58 Effects of varying test sample size. Linear SVM; Preprocessing: PCA ($n_{\text{components}}$ = all); ANOVA feature selection ($k_{\text{best}} = 1{,}000$) 90

Appendix A. Summary statistics for the final study sample

		MDD	HC
	n	934	934
**Sex**	female	556	548
	male	378	368
**Scanner**	1	285	512
	2	395	236
	3	254	186

(a) Summary of sample size (n), sex and scanner site.

		Mean	SD	Min	Max
**MDD**	Age ($n = 934$)	37.76	12.94	16	65
	TIV ($n = 933$)	1,575.94	152.97	1,115.00	2,189.00
**HC**	Age ($n = 934$)	34.15	12.42	17	65
	TIV ($n = 933$)	1,575.61	156.38	1,146.23	2,706.76

(b) Summage of Age (in years) and TIV (total intracranial volume).

Table A.1: Summary statistics for the final study sample. MDD = Major Depressive Disorder; HC = Healthy Controls.

Appendix B. Predictive Analytics Competition 2018

The medical machine learning lab from Prof. Dr. Tim Hahn invited teams from all over the world to develop a model classifying patients suffering from *Major Depressive Disorder* (MDD) and healthy individuals based on structural Magnetic Resonance Imaging (sMRI) data. This competition was called *Predictive Analytics Competition 2018* (Pac 2018).

Data

The data for this competition - comprising sMRI datasets with and without MDD from N = 2,240 subjects - was provided by the *Institute of Translational Psychiatry*, Münster. The images were preprocessed in advanced with the SPM toolbox CAT-12 (Matlab 9.0 / SMP12 rev. 6685 / CAT12 v.1184) and qualitychecked. The participants got, additional to the diagnosis, the age, gender, total intracranial volume (TIV) and the scanner-site to consider them as covariates.

The data was split into a training and test set in advance (Table B.2). The test set was held back and only used in the final step to designate the winner.

	Training	Test
Mdd	1,033	273
Hc	759	175
**Total**	1,792	448

Table B.2: Split between training and test data for the Pac 2018.

Abbreviations: Hc - Healthy Control
Mdd - Major Depressive Disorder

Timeline

Registration opened:	1st	February	2018
Training data available:	1st	March	2018
Test data available:	27th	April	2018
Test prediction upload opened:	4th	May	2018
Test prediction upload closed:	31st	May	2018

The "Pac Award Winner 2018" was announced in a ceremony held at annually meeting of the *Organization for Human Brain Mapping* (OHBM) in Singapore at the 21st June 2018.

Results

In total 49 teams registered with at least 170 participants. The winner was determined by the highest balanced accuracy score on the held-back test set. The team with the highest score was "paranoidandroid" (Table B.3) and won the Pac 2018 Award.

	Team	BA	TPR	TNR
1st	paranoidandroid	0.65	0.53	0.77
2nd	utastoot	0.64	0.51	0.77
3rd	berlin_brain_decoders	0.64	0.58	0.69
4th	depression	0.63	0.59	0.67
5th	neuronauts_p	0.63	0.61	0.64

Table B.3: Test results on the held-back test set by the top five teams at the Pac 2018.

Abbreviations: BA - balanced accuracy
TPR - true positive rate (sensitivity)
TNR - true negative rate (specificity)

Appendix C. Additional tabular presentations

This section contains the tabular presentation of the graphical visualized data in the paper.

overall sample size n	≥ accuracy (%) 50	55	60	65	70	75	80	85	90
10	0.54	0.54	0.43	0.33	0.15	0.15	0.05	0.05	0.01
20	0.64	0.52	0.40	0.24	0.13	0.06	0.01	0.01	0.00
30	0.70	0.61	0.39	0.29	0.11	0.06	0.01	0.00	0.00
40	0.78	0.63	0.47	0.23	0.09	0.02	0.00	0.00	0.00
50	0.82	0.70	0.51	0.27	0.08	0.02	0.00	0.00	0.00
60	0.81	0.62	0.41	0.19	0.05	0.01	0.00	0.00	0.00
70	0.88	0.72	0.42	0.20	0.07	0.01	0.00	0.00	0.00
80	0.90	0.73	0.48	0.18	0.04	0.00	0.00	0.00	0.00
90	0.89	0.76	0.45	0.20	0.03	0.00	0.00	0.00	0.00
100	0.93	0.75	0.47	0.17	0.02	0.00	0.00	0.00	0.00
125	0.94	0.81	0.48	0.14	0.01	0.00	0.00	0.00	0.00
150	0.96	0.83	0.45	0.12	0.01	0.00	0.00	0.00	0.00

(a) Probabilities to yield an accuracy exceeding a certain threshold related to the overall sample size.

overall sample size n	**mean**	**median**	**min**	**max**	**std**
10	0.55	0.60	0.00	1.00	0.20
20	0.56	0.60	0.10	0.95	0.15
30	0.58	0.58	0.20	0.87	0.12
40	0.59	0.60	0.25	0.82	0.10
50	0.59	0.60	0.16	0.82	0.09
60	0.58	0.58	0.32	0.82	0.08
70	0.59	0.60	0.36	0.81	0.07
80	0.59	0.60	0.36	0.76	0.07
90	0.59	0.60	0.34	0.77	0.07
100	0.60	0.60	0.35	0.81	0.06
125	0.59	0.60	0.41	0.74	0.05
150	0.59	0.59	0.41	0.73	0.05

(b) Summary of the accuracy value distributions for certain overall sample size.

Table C.4: Effects of varying overall sample sizes for training and testing a linear SVM employing LOOCV.

overall sample size	n	≥ accuracy (%) 50	55	60	65	70	75	80	85	90
	10	0.25	0.25	0.17	0.10	0.03	0.03	0.01	0.01	0.00
	20	0.32	0.18	0.10	0.04	0.02	0.01	0.00	0.00	0.00
	30	0.33	0.22	0.06	0.03	0.01	0.00	0.00	0.00	0.00
	40	0.37	0.18	0.09	0.02	0.00	0.00	0.00	0.00	0.00
	50	0.39	0.21	0.07	0.01	0.00	0.00	0.00	0.00	0.00
	60	0.40	0.16	0.04	0.01	0.00	0.00	0.00	0.00	0.00
	70	0.40	0.16	0.02	0.00	0.00	0.00	0.00	0.00	0.00
	80	0.41	0.13	0.03	0.00	0.00	0.00	0.00	0.00	0.00
	90	0.41	0.14	0.02	0.00	0.00	0.00	0.00	0.00	0.00
	100	0.43	0.11	0.02	0.00	0.00	0.00	0.00	0.00	0.00
	125	0.46	0.13	0.01	0.00	0.00	0.00	0.00	0.00	0.00
	150	0.44	0.09	0.01	0.00	0.00	0.00	0.00	0.00	0.00

(a) Probabilities to yield an accuracy exceeding a certain threshold related to the overall sample size.

overall sample size	n	**mean**	**median**	**min**	**max**	**std**
	10	0.44	0.40	0.00	1.00	0.17
	20	0.47	0.45	0.15	0.80	0.11
	30	0.48	0.47	0.23	0.80	0.09
	40	0.49	0.50	0.25	0.70	0.08
	50	0.49	0.48	0.28	0.74	0.07
	60	0.49	0.50	0.28	0.68	0.06
	70	0.49	0.50	0.31	0.69	0.06
	80	0.49	0.50	0.30	0.66	0.06
	90	0.49	0.49	0.34	0.68	0.05
	100	0.50	0.50	0.33	0.64	0.05
	125	0.50	0.50	0.33	0.63	0.05
	150	0.50	0.49	0.37	0.63	0.04

(b) Summary of the accuracy value distributions for certain overall sample size.

Table C.5: Effects of varying overall sample sizes for training and testing a Dummy Classifier employing LOOCV.

	n	≥ accuracy (%) 50	55	60	65	70	75	80	85	90
**train sample size**	10	0.80	0.62	0.42	0.16	0.00	0.00	0.00	0.00	0.00
	20	0.87	0.75	0.55	0.20	0.00	0.00	0.00	0.00	0.00
	30	0.94	0.83	0.65	0.26	0.00	0.00	0.00	0.00	0.00
	40	0.97	0.90	0.72	0.29	0.00	0.00	0.00	0.00	0.00
	50	0.97	0.93	0.76	0.31	0.00	0.00	0.00	0.00	0.00
	60	0.99	0.95	0.76	0.30	0.00	0.00	0.00	0.00	0.00
	70	0.99	0.96	0.83	0.35	0.00	0.00	0.00	0.00	0.00
	80	0.99	0.97	0.83	0.34	0.00	0.00	0.00	0.00	0.00
	90	1.00	0.98	0.89	0.36	0.00	0.00	0.00	0.00	0.00
	100	1.00	0.98	0.87	0.35	0.00	0.00	0.00	0.00	0.00
	125	1.00	0.99	0.91	0.39	0.00	0.00	0.00	0.00	0.00
	150	1.00	0.99	0.94	0.39	0.00	0.00	0.00	0.00	0.00

(a) Probabilities to yield an accuracy exceeding a certain threshold related to the train sample size.

	n	**mean**	**median**	**min**	**max**	**std**
**train sample size**	10	0.57	0.58	0.33	0.69	0.08
	20	0.59	0.61	0.32	0.69	0.07
	30	0.61	0.62	0.36	0.69	0.06
	40	0.62	0.63	0.35	0.70	0.05
	50	0.62	0.63	0.38	0.70	0.05
	60	0.63	0.64	0.46	0.70	0.04
	70	0.63	0.64	0.45	0.71	0.04
	80	0.63	0.64	0.45	0.70	0.03
	90	0.64	0.64	0.49	0.71	0.03
	100	0.64	0.64	0.51	0.70	0.03
	125	0.64	0.64	0.51	0.71	0.03
	150	0.64	0.65	0.51	0.70	0.03

(b) Summary of the accuracy value distributions for certain train sample size.

Table C.6: Results of a fixed validation set of $n = 300$ and varying train sample sizes on a linear SVM using default parameter. ($C = 1$)

		≥ accuracy (%)								
	n	50	55	60	65	70	75	80	85	90
**train sample size**	10	0.49	0.04	0.00	0.00	0.00	0.00	0.00	0.00	0.00
	20	0.47	0.04	0.00	0.00	0.00	0.00	0.00	0.00	0.00
	30	0.47	0.04	0.00	0.00	0.00	0.00	0.00	0.00	0.00
	40	0.47	0.03	0.00	0.00	0.00	0.00	0.00	0.00	0.00
	50	0.45	0.04	0.00	0.00	0.00	0.00	0.00	0.00	0.00
	60	0.46	0.04	0.00	0.00	0.00	0.00	0.00	0.00	0.00
	70	0.48	0.03	0.00	0.00	0.00	0.00	0.00	0.00	0.00
	80	0.49	0.04	0.00	0.00	0.00	0.00	0.00	0.00	0.00
	90	0.47	0.03	0.00	0.00	0.00	0.00	0.00	0.00	0.00
	100	0.48	0.05	0.00	0.00	0.00	0.00	0.00	0.00	0.00
	125	0.45	0.03	0.00	0.00	0.00	0.00	0.00	0.00	0.00
	150	0.45	0.03	0.00	0.00	0.00	0.00	0.00	0.00	0.00

(a) Probabilities to yield an accuracy exceeding a certain threshold related to the train sample size.

	n	**mean**	**median**	**min**	**max**	**std**
**train sample size**	10	0.50	0.50	0.41	0.59	0.03
	20	0.50	0.50	0.39	0.59	0.03
	30	0.50	0.50	0.41	0.58	0.03
	40	0.50	0.50	0.38	0.60	0.03
	50	0.50	0.50	0.42	0.61	0.03
	60	0.50	0.50	0.40	0.60	0.03
	70	0.50	0.50	0.41	0.58	0.03
	80	0.50	0.50	0.42	0.62	0.03
	90	0.50	0.50	0.42	0.58	0.03
	100	0.50	0.50	0.40	0.60	0.03
	125	0.50	0.50	0.40	0.59	0.03
	150	0.50	0.50	0.39	0.61	0.03

(b) Summary of the accuracy value distributions for certain train sample size.

Table C.7: Results of a fixed validation set of $n = 300$ and varying train sample sizes on a Dummy Classifier.

test sample size: n	≥ accuracy (%): 50	55	60	65	70	75	80	85	90
10	0.78	0.78	0.66	0.54	0.29	0.29	0.10	0.10	0.02
20	0.91	0.81	0.69	0.48	0.30	0.17	0.06	0.06	0.00
30	0.96	0.90	0.73	0.57	0.27	0.15	0.02	0.01	0.00
40	0.98	0.91	0.80	0.49	0.25	0.09	0.02	0.01	0.00
50	0.99	0.97	0.85	0.58	0.22	0.07	0.00	0.00	0.00
60	1.00	0.97	0.84	0.56	0.23	0.05	0.00	0.00	0.00
70	1.00	0.99	0.88	0.61	0.21	0.04	0.00	0.00	0.00
80	1.00	0.99	0.90	0.56	0.17	0.02	0.00	0.00	0.00
90	1.00	1.00	0.94	0.63	0.17	0.02	0.00	0.00	0.00
100	1.00	0.99	0.94	0.58	0.13	0.01	0.00	0.00	0.00
125	1.00	1.00	0.97	0.63	0.13	0.00	0.00	0.00	0.00
150	1.00	1.00	0.98	0.66	0.08	0.00	0.00	0.00	0.00

(a) Probabilities to yield an accuracy exceeding a certain threshold related to the test sample size.

test sample size: n	**mean**	**median**	**min**	**max**	**std**
10	0.66	0.70	0.20	1.00	0.15
20	0.67	0.65	0.35	0.95	0.11
30	0.66	0.67	0.40	0.93	0.08
40	0.66	0.65	0.42	0.88	0.07
50	0.66	0.66	0.48	0.88	0.06
60	0.66	0.67	0.47	0.83	0.06
70	0.66	0.67	0.50	0.83	0.05
80	0.66	0.66	0.49	0.80	0.05
90	0.66	0.67	0.53	0.78	0.04
100	0.66	0.66	0.51	0.79	0.04
125	0.66	0.66	0.54	0.78	0.03
150	0.66	0.66	0.56	0.75	0.03

(b) Summary of the accuracy value distributions for certain test sample size.

Table C.8: Effects of varying test sample sizes on a fixed pretrained linear SVM.

		≥ accuracy (%)								
	n	50	55	60	65	70	75	80	85	90
**test sample size**	10	0.39	0.39	0.29	0.17	0.05	0.05	0.01	0.01	0.00
	20	0.42	0.24	0.13	0.05	0.02	0.01	0.00	0.00	0.00
	30	0.42	0.28	0.10	0.05	0.01	0.00	0.00	0.00	0.00
	40	0.44	0.19	0.08	0.01	0.00	0.00	0.00	0.00	0.00
	50	0.44	0.20	0.06	0.01	0.00	0.00	0.00	0.00	0.00
	60	0.45	0.15	0.03	0.00	0.00	0.00	0.00	0.00	0.00
	70	0.45	0.17	0.02	0.00	0.00	0.00	0.00	0.00	0.00
	80	0.44	0.10	0.01	0.00	0.00	0.00	0.00	0.00	0.00
	90	0.43	0.12	0.01	0.00	0.00	0.00	0.00	0.00	0.00
	100	0.47	0.10	0.01	0.00	0.00	0.00	0.00	0.00	0.00
	125	0.53	0.09	0.00	0.00	0.00	0.00	0.00	0.00	0.00
	150	0.45	0.04	0.00	0.00	0.00	0.00	0.00	0.00	0.00

(a) Probabilities to yield an accuracy exceeding a certain threshold related to the test sample size.

	n	**mean**	**median**	**min**	**max**	**std**
**test sample size**	10	0.50	0.50	0.10	1.00	0.16
	20	0.50	0.50	0.15	0.85	0.11
	30	0.50	0.50	0.23	0.73	0.09
	40	0.50	0.50	0.28	0.72	0.07
	50	0.50	0.50	0.32	0.70	0.06
	60	0.50	0.50	0.30	0.67	0.06
	70	0.50	0.50	0.34	0.67	0.05
	80	0.50	0.50	0.35	0.64	0.05
	90	0.50	0.50	0.36	0.67	0.04
	100	0.50	0.50	0.39	0.63	0.04
	125	0.50	0.50	0.39	0.62	0.04
	150	0.50	0.50	0.40	0.59	0.03

(b) Summary of the accuracy value distributions for certain test sample size.

Table C.9: Effects of varying test sample sizes on a fixed pretrained Dummy Classifier.

Appendix D. Influence of the scanner distribution

Scanner site distributions were not well balanced in the PAC sample. This imbalance was even stronger in our randomly drawn sub-samples. To determine the influence of the different scanner-sites on model accuracy, we took the same methodical approach that we used for the "train sample size effect analysis" and determined the scanner distribution for each set. Following, we calculated Spearman's rho between the scanner-distribution and the accuracy of the hold-out test set ($n = 300$). The scanner-distribution for each set was approximated using Gini's index. Here, we show that the scanner distribution has a statistically significant influence on test set accuracy but explains only 0.79 % of the variance.

Appendix E. Adjustment of SVM regularization based on sample size

The regularization of a linear SVM depends on the absolute number of outliers in a sample. To exclude the possibility that the use of default hyperparameters (a constant value of $C = 1$) caused the effect that we observed, we have adjusted the SVM regularization (our C hyperparameter) based on the size of each analyses sub-sample. In this adjustment, a constant value k is divided by the sample size. To approximate the default parameter $C = 1$ on average, we have set $k = 75$. For example, C would be $C = 100/75 = 1.33$ for a sample size of $n = 100$. In this case, the adjusted regularization did not deliver results as good as what we observed with $C = 1$ (see Figure E.1, E.2). Therefore, we conclude that the default value of $C = 1$ across changing N values did not increase the probability of misestimating accuracy as sample size decreased.

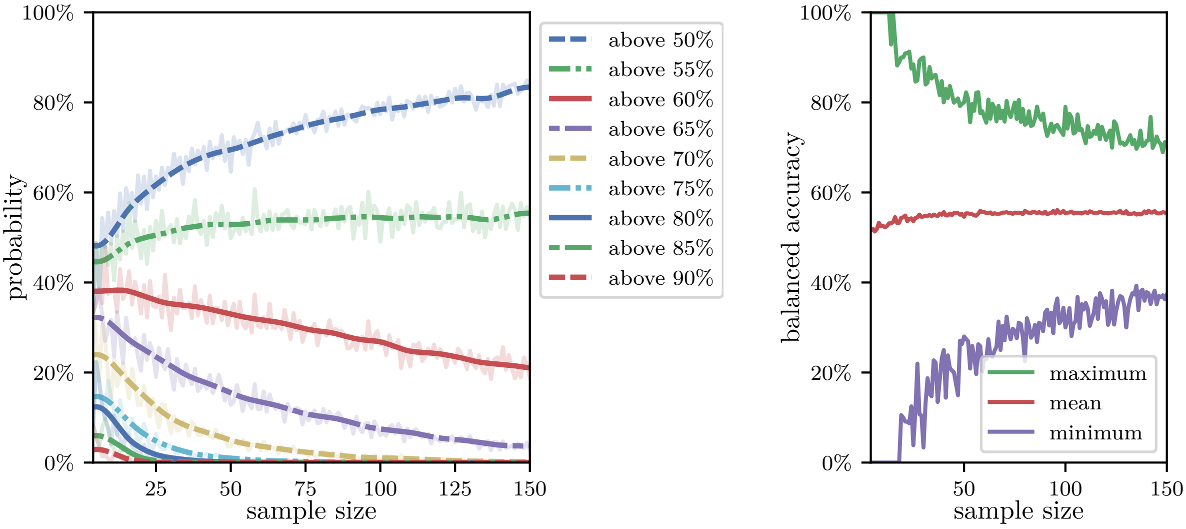

(a) Probabilities for linear SVMs to yield an accuracy exceeding a certain threshold as a function of sample size employing LOOCV.

(b) Minimum, maximum and mean results for the linear SVMs as a function of sample size employing LOOCV.

Figure E.1: Effects of varying overall sample sizes employing LOOCV with an adjusted C parameter with $C = \frac{75}{\text{sample size}}$.

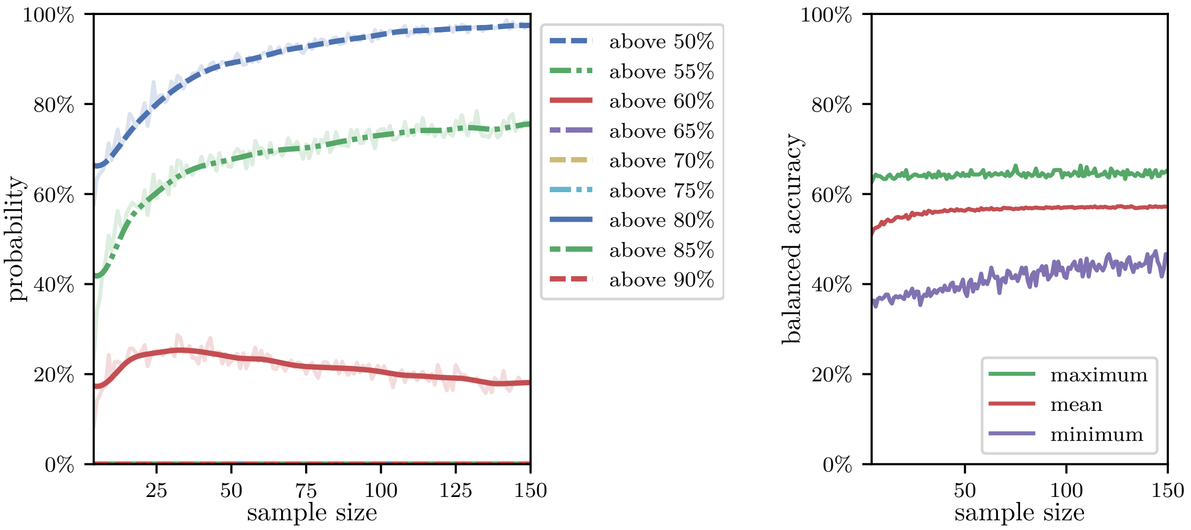

(a) Probabilities for linear SVMs to yield an accuracy exceeding a certain threshold as a function of training sample size.

(b) Minimum, maximum and mean results for the linear SVMs as a function of training sample size.

Figure E.2: Results as a function of training set sizes with a fixed test set size of $N = 300$ and an adjusted C parameter with $C = \frac{75}{\text{sample size}}$.

Appendix F. Alternative Machine Configurations

To exclude the possibility that the observed effects can only be traced back to our specific configuration, we have tested other usual configurations. The configurations consist of a preprocessing and a classification. In the preprocessing step, we used a method for reduction of feature space and a method for features selection. The reduction of the feature space was achieved with a Principal Component Analysis (PCA), where only a certain number the first components were used. Afterwards, for feature selection, an ANOVA was calculated and a specific number of feature beginning with the highest F-value were taken. The actual configuration used for preprocessing is listed in Table F.10. For the classification, we chose three specific machines:

1. An SVM with a linear kernel and default parameter. ($C = 1.0$)
2. An SVM with an RBF kernel and default parameter ($C = 1.0$; $\gamma = 1/n_{\text{feature}}$)
3. A Random Forrest and default parameter ($n_{\text{estimators}} = 100$)

In combination with the preprocessing, this results in 48 configurations. Since the found effect is limited to the test set size, we have only repeated our analyses for the test set for each of these configurations.

		**PCA $n_{\mathbf{components}}$**				
		no	all	500	50	10
**ANOVA $k_{\mathbf{best}}$**	no	×	×	×	×	×
	10	×	×	×	×	
	100	×	×	×		
	1,000	×	×			
	10,000	×				
	50,000	×				

Table F.10: The × marks the used combinations of configurations for the preprocessing. The *no*-label means that this preprocessing step were left out.

Appendix F.1. Results

The results are comparable to the originally used configuration, an SVM with a linear kernel and no preprocessing (see Figure F.3 to F.50 and Table F.11 to F.58). The results thus underline a generally valid character of the findings. An outlier in the results can be found for a specific configuration, a PCA with 10 components and the SVM with an RBF kernel (see Figure F.22 and Table F.30). This result can be explained due to overfitting, the machine constantly returns one constant class as a prediction.

Appendix F.1.1. Graphical results representation

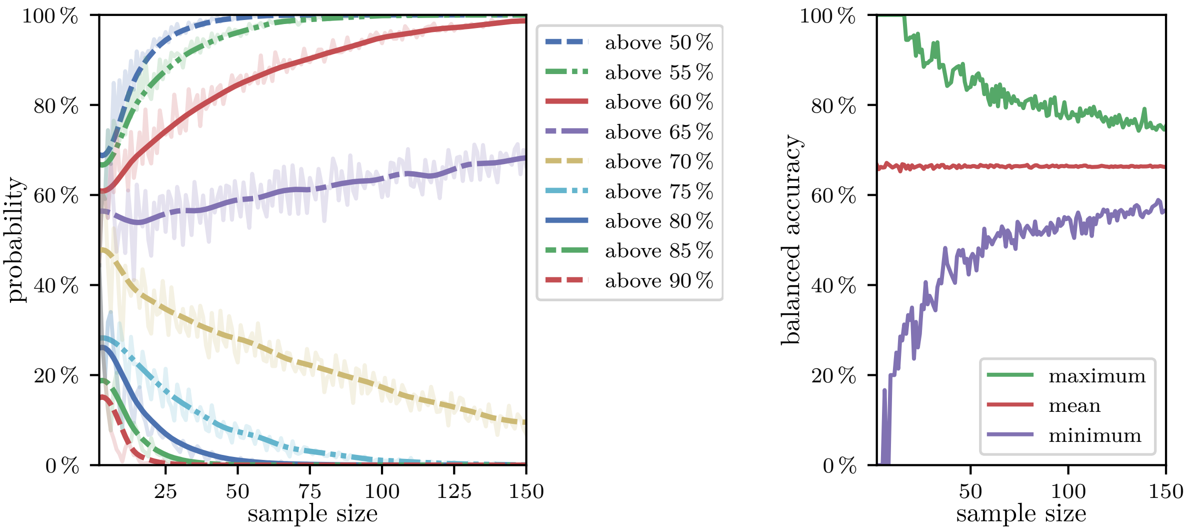

(a) Probabilities for Random Forest to yield an accuracy exceeding a certain threshold as a function of test sample size.

(b) Minimum, maximum and mean results for the Random Forest as a function of test sample size.

Figure F.3: Results as a function of variable test set sizes with a fixed classifier. A **Random Forest** was trained with default parameters. ($n_{\text{estimators}} = 100$)

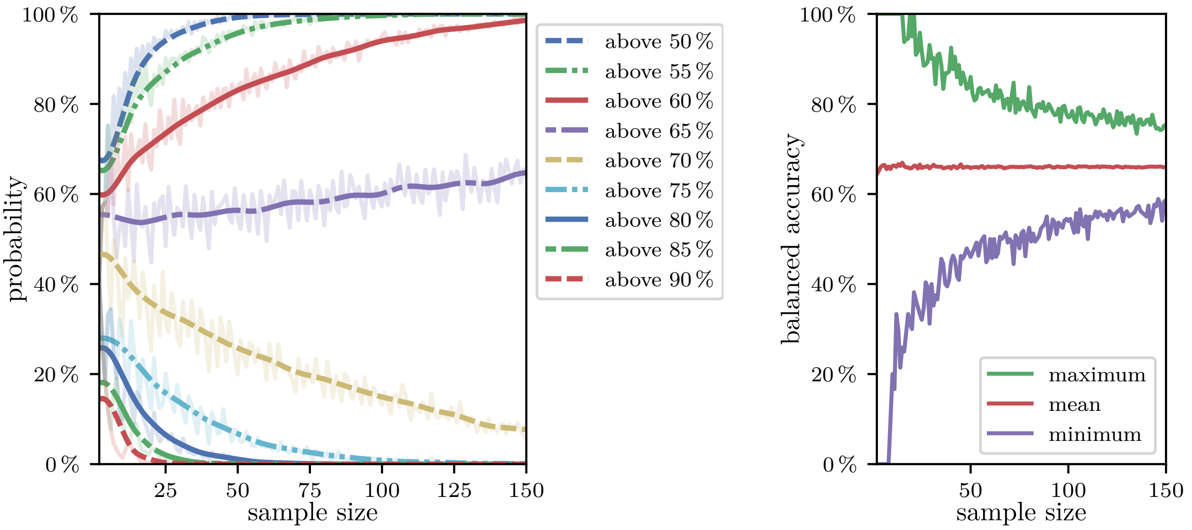

(a) Probabilities for SVM with an RBF kernel to yield an accuracy exceeding a certain threshold as a function of test sample size.

(b) Minimum, maximum and mean results for the SVM with an RBF kernel as a function of test sample size.

Figure F.4: Results as a function of variable test set sizes with a fixed classifier. An **SVM** with an **RBF kernel** was trained with default parameters. ($C = 1.0$; $\gamma = {}^1/_{n_{\text{feature}}}$)

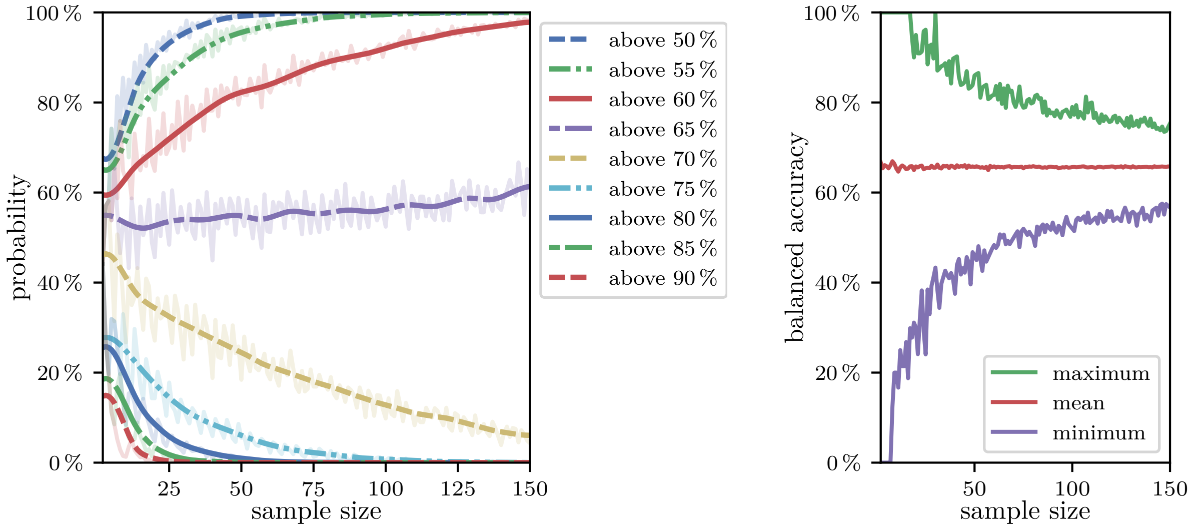

(a) Probabilities for linear SVM to yield an accuracy exceeding a certain threshold as a function of test sample size.

(b) Minimum, maximum and mean results for the linear SVM as a function of test sample size.

Figure F.5: Results as a function of variable test set sizes with a fixed classifier. A **linear SVM** was trained with default parameters. ($C = 1.0$)

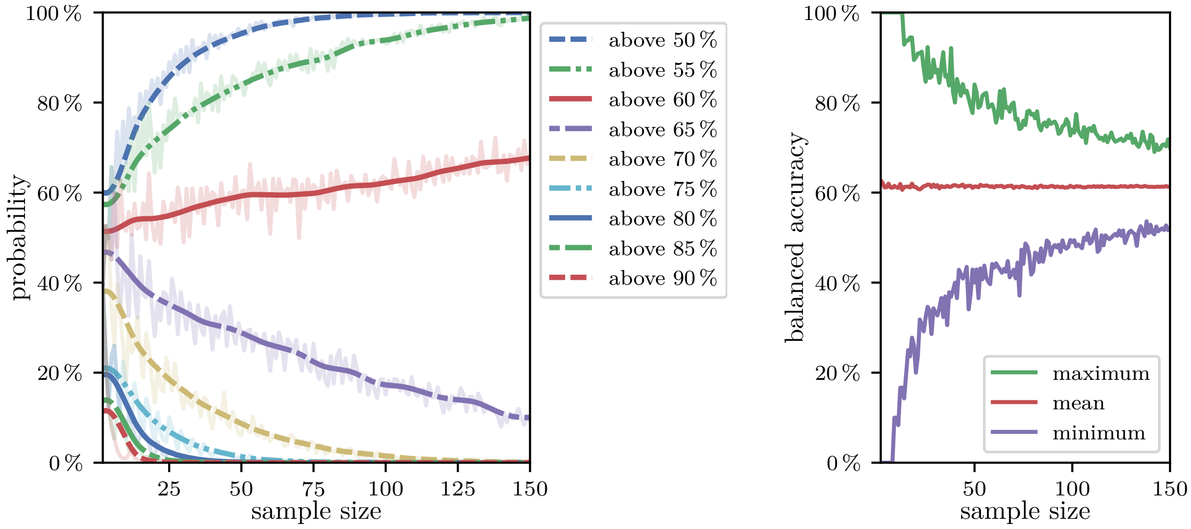

(a) Probabilities for Random Forest to yield an accuracy exceeding a certain threshold as a function of test sample size.

(b) Minimum, maximum and mean results for the Random Forest as a function of test sample size.

Figure F.6: Results as a function of variable test set sizes with a fixed classifier. For **feature selection** an ANOVA was computed inside the the pipeline and the top 10 **features** were taken based on the ANOVA F-values. Following, a **Random Forest** was trained with default parameters. ($n_{\text{estimators}} = 100$)

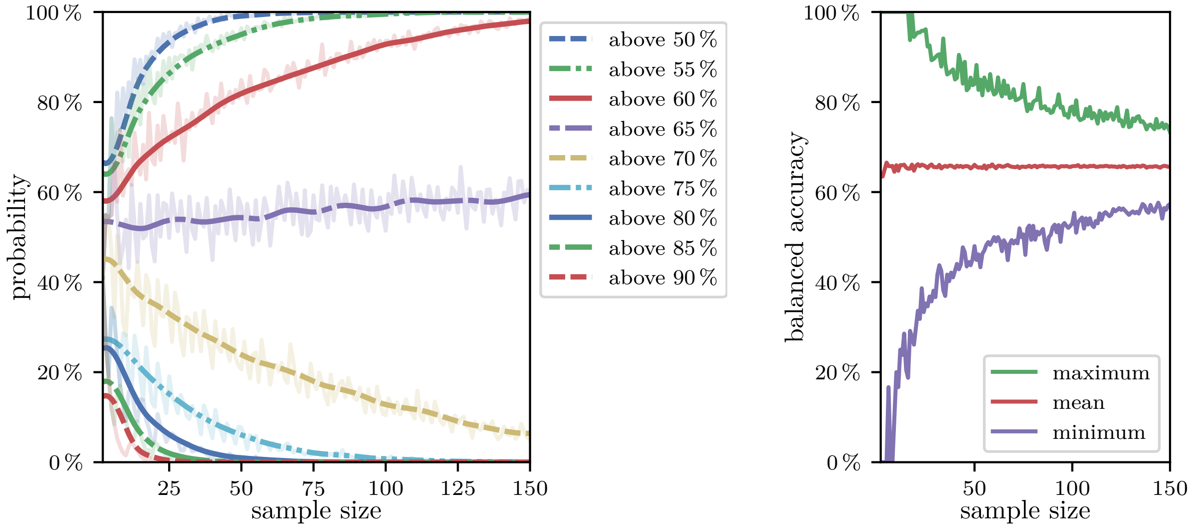

(a) Probabilities for SVM with an RBF kernel to yield an accuracy exceeding a certain threshold as a function of test sample size.

(b) Minimum, maximum and mean results for the SVM with an RBF kernel as a function of test sample size.

Figure F.7: Results as a function of variable test set sizes with a fixed classifier. For **feature selection** an ANOVA was computed inside the the pipeline and the top 10 **features** were taken based on the ANOVA F-values. Following, an **SVM** with an **RBF kernel** was trained with default parameters. ($C = 1.0$; $\gamma = {}^{1}/{n_{\text{feature}}}$)

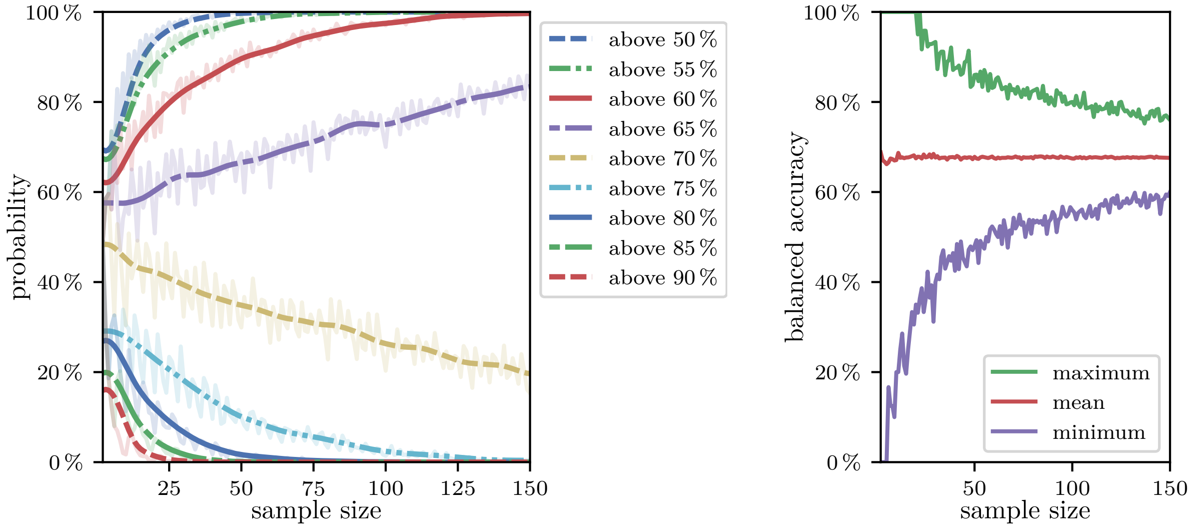

(a) Probabilities for linear SVM to yield an accuracy exceeding a certain threshold as a function of test sample size.

(b) Minimum, maximum and mean results for the linear SVM as a function of test sample size.

Figure F.8: Results as a function of variable test set sizes with a fixed classifier. For **feature selection** an ANOVA was computed inside the the pipeline and the top 10 **features** were taken based on the ANOVA F-values. Following, a **linear SVM** was trained with default parameters. ($C = 1.0$)

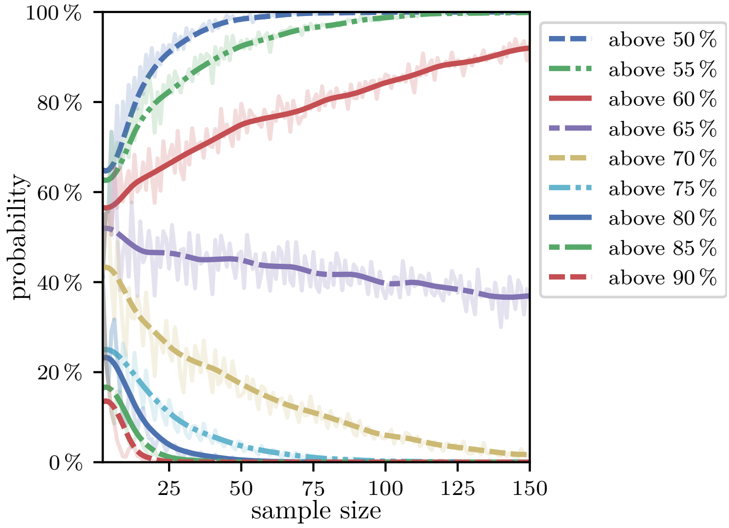

(a) Probabilities for Random Forest to yield an accuracy exceeding a certain threshold as a function of test sample size.

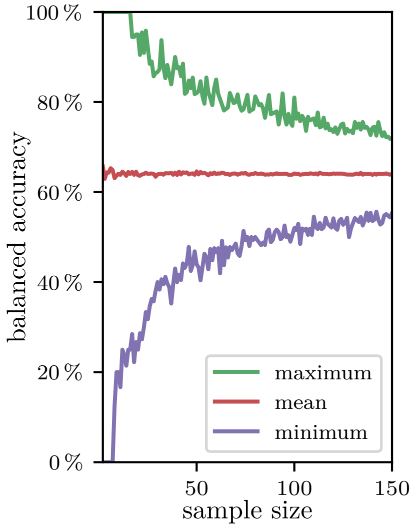

(b) Minimum, maximum and mean results for the Random Forest as a function of test sample size.

Figure F.9: Results as a function of variable test set sizes with a fixed classifier. For **feature selection** an ANOVA was computed inside the the pipeline and the top 100 **features** were taken based on the ANOVA F-values. Following, a **Random Forest** was trained with default parameters. ($n_{\text{estimators}} = 100$)

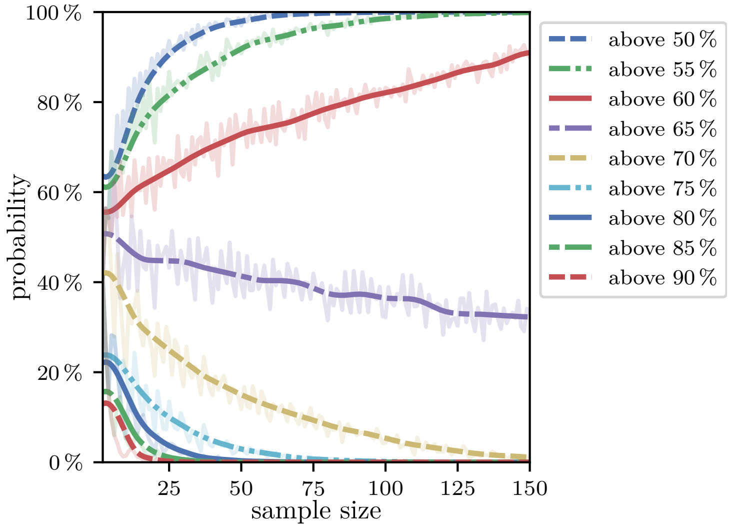

(a) Probabilities for SVM with an RBF kernel to yield an accuracy exceeding a certain threshold as a function of test sample size.

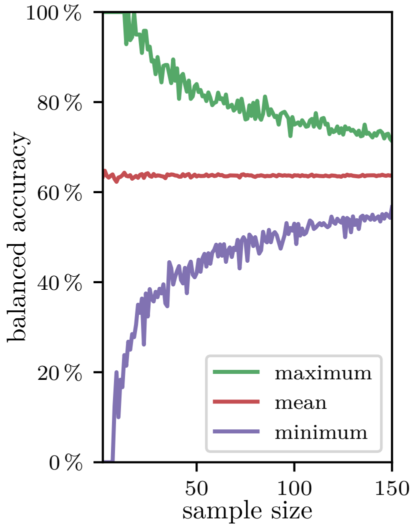

(b) Minimum, maximum and mean results for the SVM with an RBF kernel as a function of test sample size.

Figure F.10: Results as a function of variable test set sizes with a fixed classifier. For **feature selection** an ANOVA was computed inside the the pipeline and the top 100 **features** were taken based on the ANOVA F-values. Following, an **SVM** with an **RBF kernel** was trained with default parameters. ($C = 1.0$; $\gamma = {}^{1}/{n_{\text{feature}}}$)

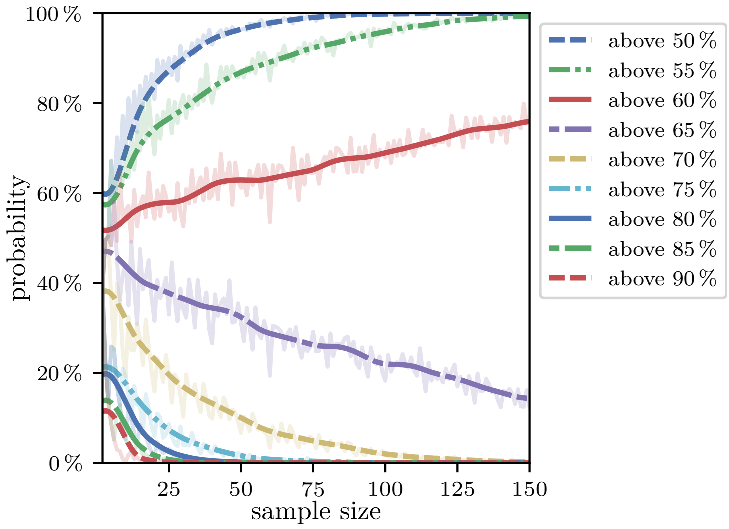

(a) Probabilities for linear SVM to yield an accuracy exceeding a certain threshold as a function of test sample size.

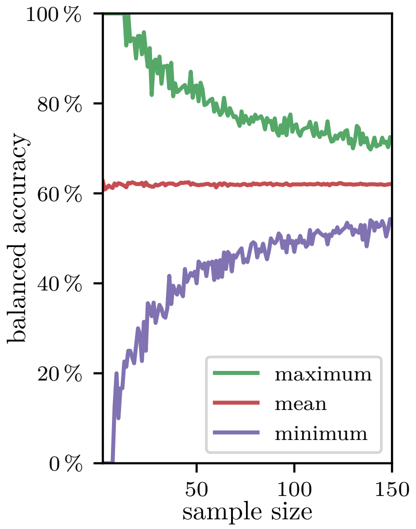

(b) Minimum, maximum and mean results for the linear SVM as a function of test sample size.

Figure F.11: Results as a function of variable test set sizes with a fixed classifier. For **feature selection** an ANOVA was computed inside the the pipeline and the top 100 **features** were taken based on the ANOVA F-values. Following, a **linear SVM** was trained with default parameters. ($C = 1.0$)

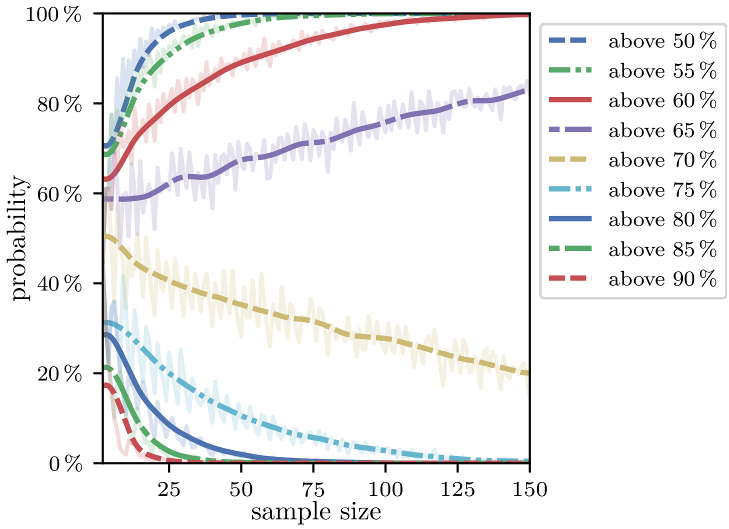

(a) Probabilities for Random Forest to yield an accuracy exceeding a certain threshold as a function of test sample size.

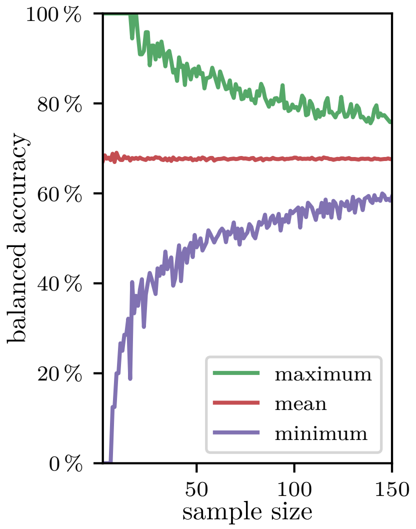

(b) Minimum, maximum and mean results for the Random Forest as a function of test sample size.

Figure F.12: Results as a function of variable test set sizes with a fixed classifier. For **feature selection** an ANOVA was computed inside the the pipeline and the top 1,000 **features** were taken based on the ANOVA F-values. Following, a **Random Forest** was trained with default parameters. ($n_{\text{estimators}} = 100$)

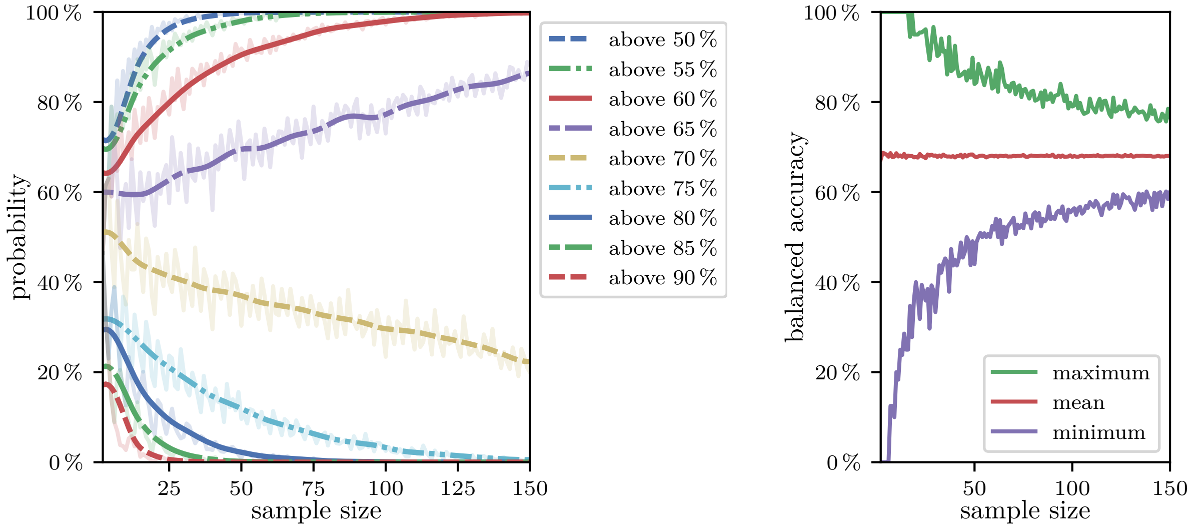

(a) Probabilities for SVM with an RBF kernel to yield an accuracy exceeding a certain threshold as a function of test sample size.

(b) Minimum, maximum and mean results for the SVM with an RBF kernel as a function of test sample size.

Figure F.13: Results as a function of variable test set sizes with a fixed classifier. For **feature selection** an ANOVA was computed inside the the pipeline and the top 1,000 **features** were taken based on the ANOVA F-values. Following, an **SVM** with an **RBF kernel** was trained with default parameters. ($C = 1.0$; $\gamma = {}^{1}/{n_{\text{feature}}}$)

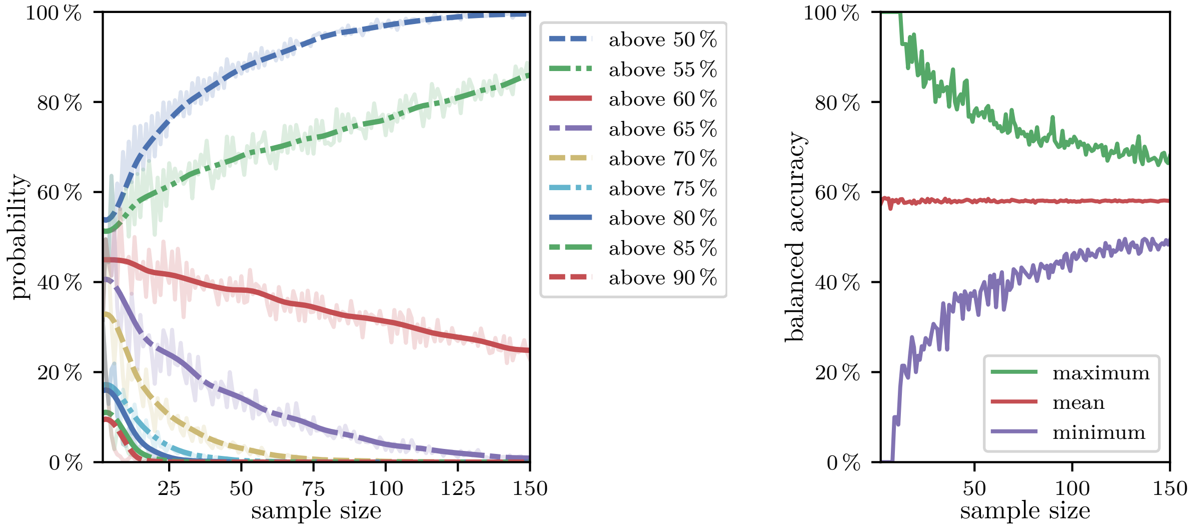

(a) Probabilities for linear SVM to yield an accuracy exceeding a certain threshold as a function of test sample size.

(b) Minimum, maximum and mean results for the linear SVM as a function of test sample size.

Figure F.14: Results as a function of variable test set sizes with a fixed classifier. For **feature selection** an ANOVA was computed inside the the pipeline and the top 1,000 **features** were taken based on the ANOVA F-values. Following, a **linear SVM** was trained with default parameters. ($C = 1.0$)

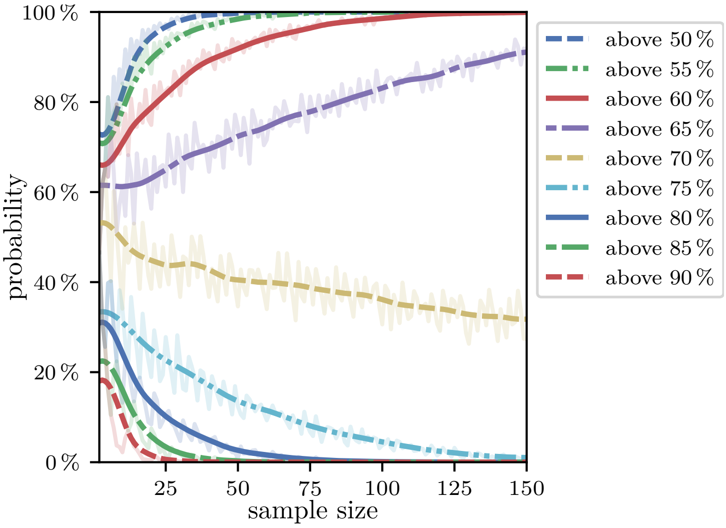

(a) Probabilities for Random Forest to yield an accuracy exceeding a certain threshold as a function of test sample size.

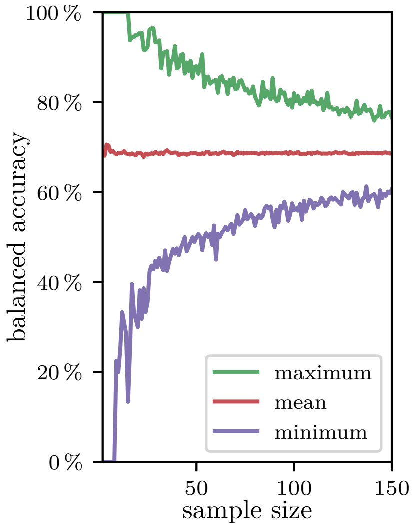

(b) Minimum, maximum and mean results for the Random Forest as a function of test sample size.

Figure F.15: Results as a function of variable test set sizes with a fixed classifier. For **feature selection** an ANOVA was computed inside the the pipeline and the top 10,000 **features** were taken based on the ANOVA F-values. Following, a **Random Forest** was trained with default parameters. ($n_{\text{estimators}} = 100$)

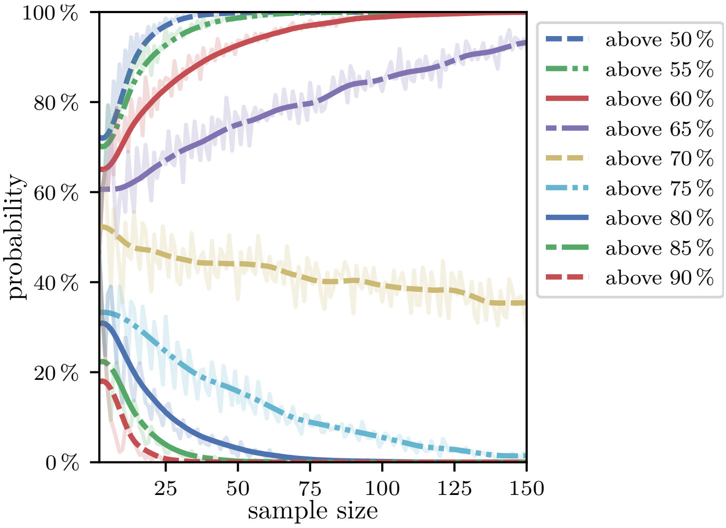

(a) Probabilities for SVM with an RBF kernel to yield an accuracy exceeding a certain threshold as a function of test sample size.

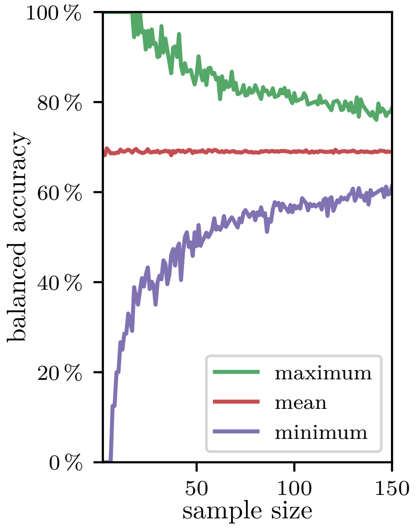

(b) Minimum, maximum and mean results for the SVM with an RBF kernel as a function of test sample size.

Figure F.16: Results as a function of variable test set sizes with a fixed classifier. For **feature selection** an ANOVA was computed inside the the pipeline and the top 10,000 **features** were taken based on the ANOVA F-values. Following, an **SVM** with an **RBF kernel** was trained with default parameters. ($C = 1.0$; $\gamma = {}^{1}/{n_{\text{feature}}}$)

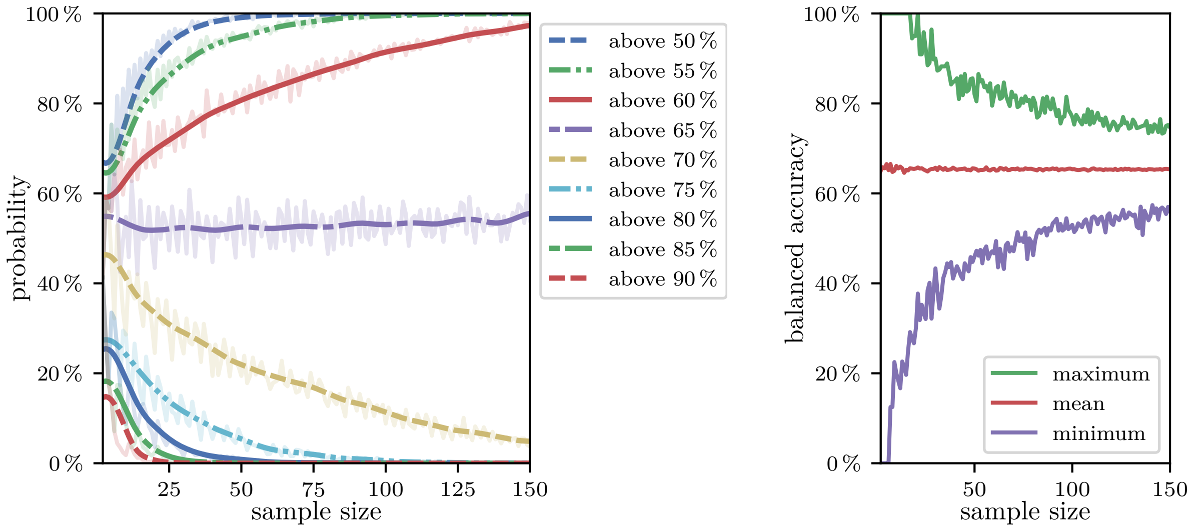

(a) Probabilities for linear SVM to yield an accuracy exceeding a certain threshold as a function of test sample size.

(b) Minimum, maximum and mean results for the linear SVM as a function of test sample size.

Figure F.17: Results as a function of variable test set sizes with a fixed classifier. For **feature selection** an ANOVA was computed inside the the pipeline and the top 10,000 **features** were taken based on the ANOVA F-values. Following, a **linear SVM** was trained with default parameters. ($C = 1.0$)

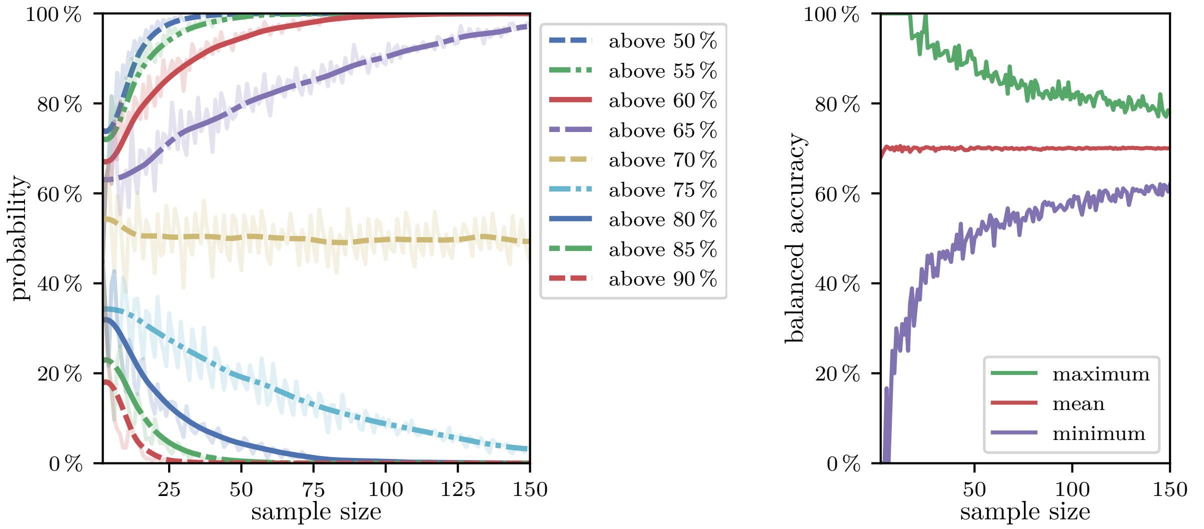

(a) Probabilities for Random Forest to yield an accuracy exceeding a certain threshold as a function of test sample size.

(b) Minimum, maximum and mean results for the Random Forest as a function of test sample size.

Figure F.18: Results as a function of variable test set sizes with a fixed classifier. For **feature selection** an ANOVA was computed inside the the pipeline and the top 50,000 **features** were taken based on the ANOVA F-values. Following, a **Random Forest** was trained with default parameters. ($n_{\text{estimators}} = 100$)

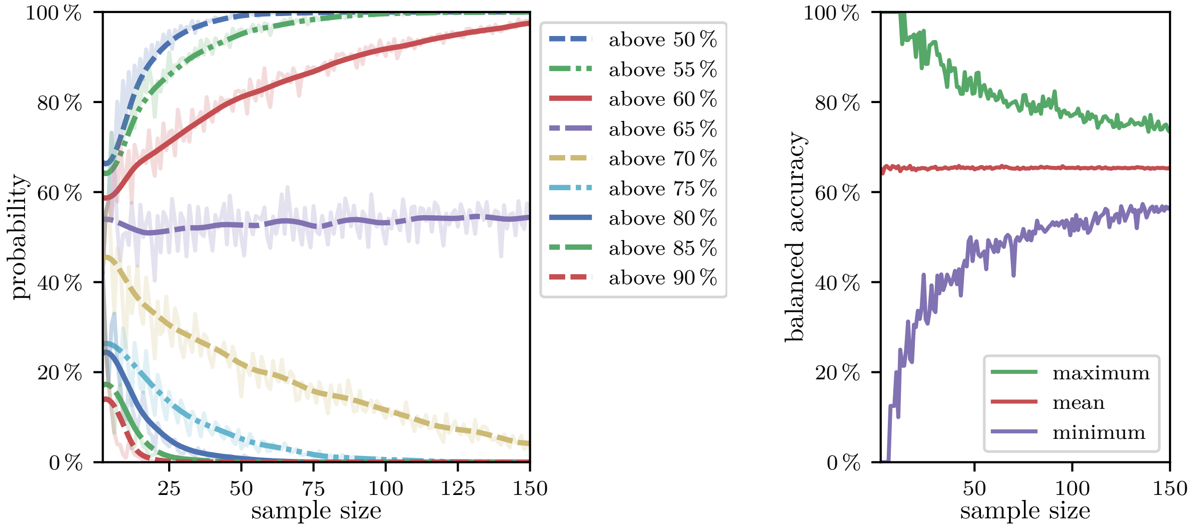

(a) Probabilities for SVM with an RBF kernel to yield an accuracy exceeding a certain threshold as a function of test sample size.

(b) Minimum, maximum and mean results for the SVM with an RBF kernel as a function of test sample size.

Figure F.19: Results as a function of variable test set sizes with a fixed classifier. For **feature selection** an ANOVA was computed inside the the pipeline and the top 50,000 **features** were taken based on the ANOVA F-values. Following, an **SVM** with an **RBF kernel** was trained with default parameters. ($C = 1.0$; $\gamma = {}^1/n_{\text{feature}}$)

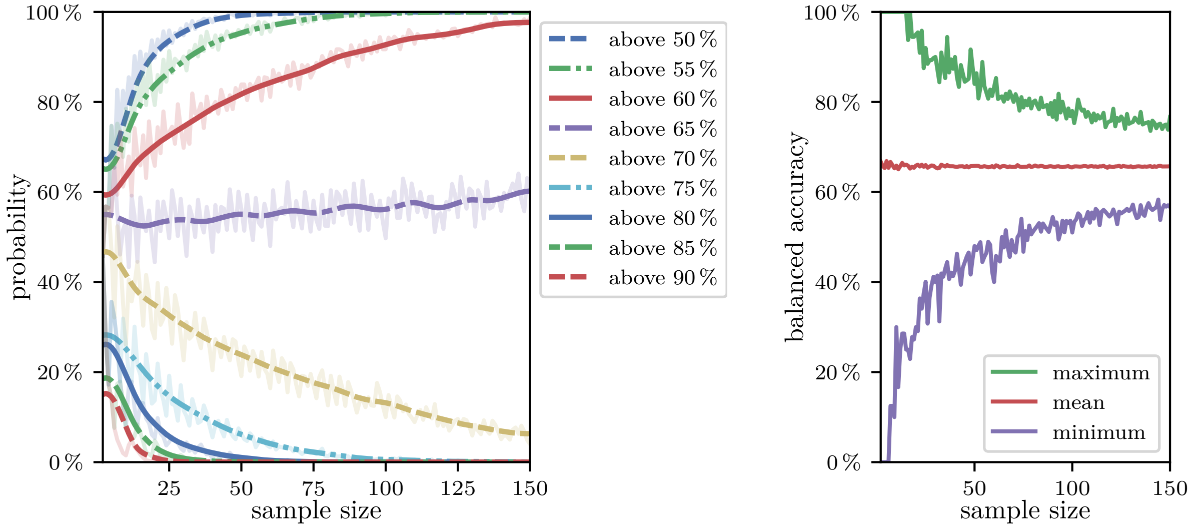

(a) Probabilities for linear SVM to yield an accuracy exceeding a certain threshold as a function of test sample size.

(b) Minimum, maximum and mean results for the linear SVM as a function of test sample size.

Figure F.20: Results as a function of variable test set sizes with a fixed classifier. For **feature selection** an ANOVA was computed inside the the pipeline and the top 50,000 **features** were taken based on the ANOVA F-values. Following, a **linear SVM** was trained with default parameters. ($C = 1.0$)

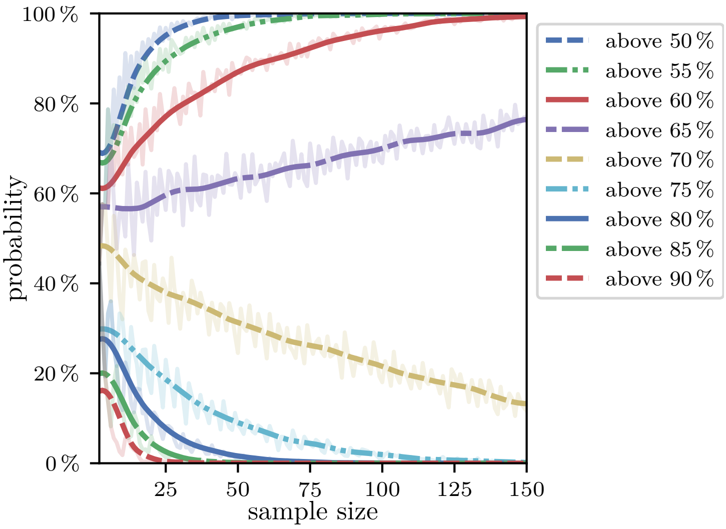

(a) Probabilities for Random Forest to yield an accuracy exceeding a certain threshold as a function of test sample size.

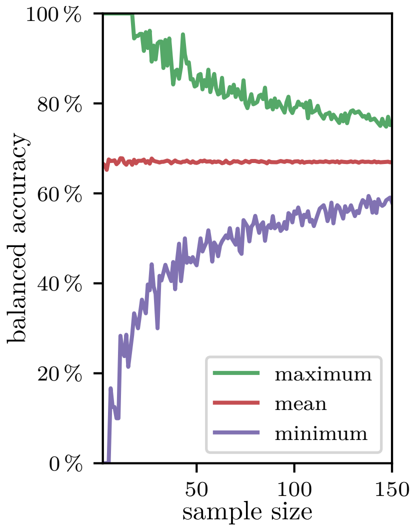

(b) Minimum, maximum and mean results for the Random Forest as a function of test sample size.

Figure F.21: Results as a function of variable test set sizes with a fixed classifier. To reduce the dimensionality of the feature space a **PCA** was performed and 10 **components** were retained. Following, a **Random Forest** was trained with default parameters. ($n_{\text{estimators}} = 100$)

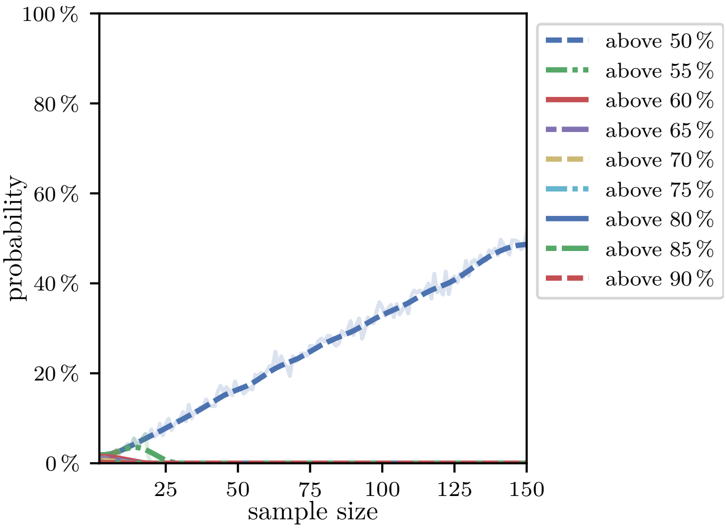

(a) Probabilities for SVM with an RBF kernel to yield an accuracy exceeding a certain threshold as a function of test sample size.

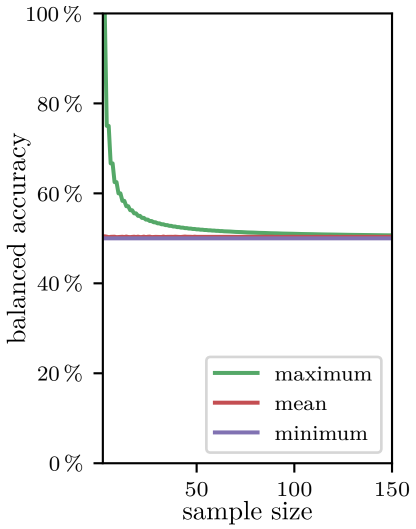

(b) Minimum, maximum and mean results for the SVM with an RBF kernel as a function of test sample size.

Figure F.22: Results as a function of variable test set sizes with a fixed classifier. To reduce the dimensionality of the feature space a **PCA** was performed and 10 **components** were retained. Following, an **SVM** with an **RBF kernel** was trained with default parameters. ($C = 1.0$; $\gamma = {}^{1}/_{n_{\text{feature}}}$)

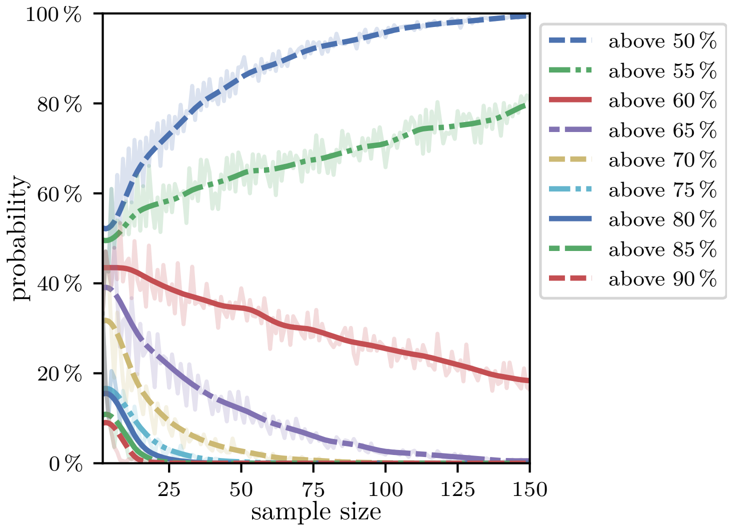

(a) Probabilities for linear SVM to yield an accuracy exceeding a certain threshold as a function of test sample size.

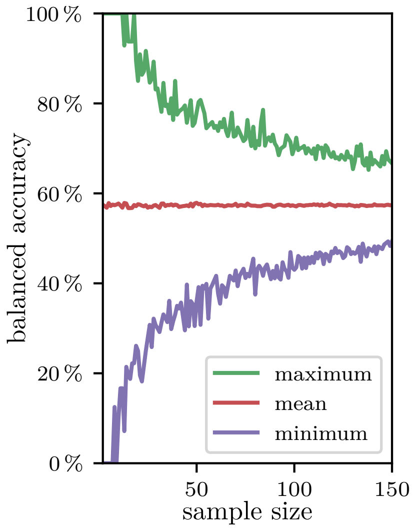

(b) Minimum, maximum and mean results for the linear SVM as a function of test sample size.

Figure F.23: Results as a function of variable test set sizes with a fixed classifier. To reduce the dimensionality of the feature space a **PCA** was performed and 10 **components** were retained. Following, a **linear SVM** was trained with default parameters. ($C = 1.0$)

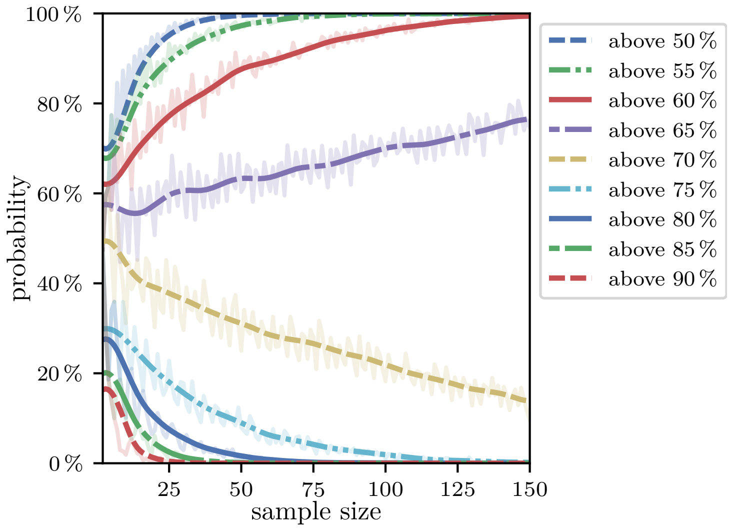

(a) Probabilities for Random Forest to yield an accuracy exceeding a certain threshold as a function of test sample size.

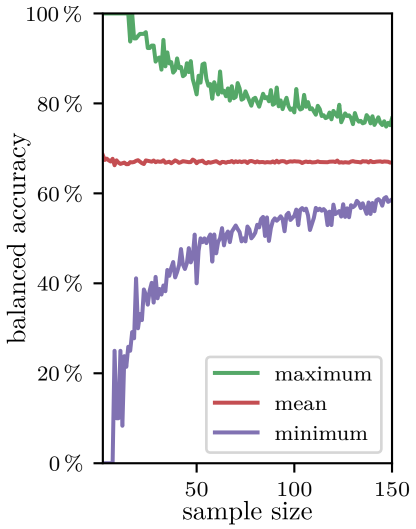

(b) Minimum, maximum and mean results for the Random Forest as a function of test sample size.

Figure F.24: Results as a function of variable test set sizes with a fixed classifier. To reduce the dimensionality of the feature space a **PCA** was performed and 50 **components** were retained. Following, a **Random Forest** was trained with default parameters. ($n_{\text{estimators}} = 100$)

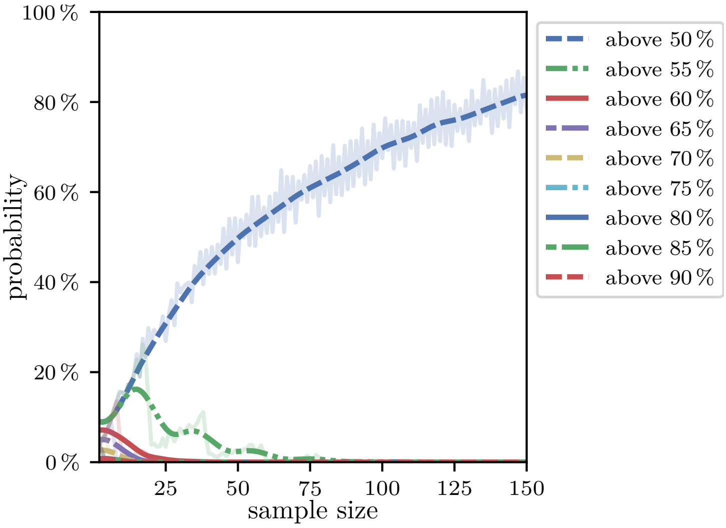

(a) Probabilities for SVM with an RBF kernel to yield an accuracy exceeding a certain threshold as a function of test sample size.

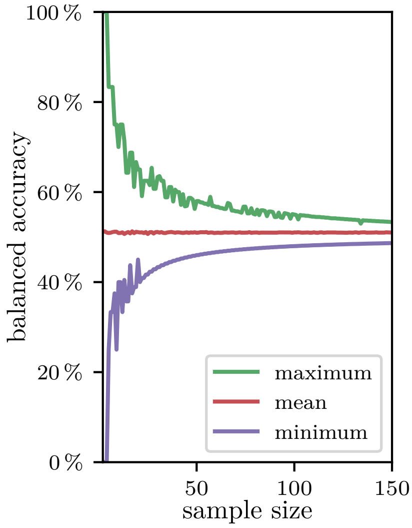

(b) Minimum, maximum and mean results for the SVM with an RBF kernel as a function of test sample size.

Figure F.25: Results as a function of variable test set sizes with a fixed classifier. To reduce the dimensionality of the feature space a **PCA** was performed and 50 **components** were retained. Following, an **SVM** with an **RBF kernel** was trained with default parameters. ($C = 1.0$; $\gamma = {}^{1}/{n_{\text{feature}}}$)

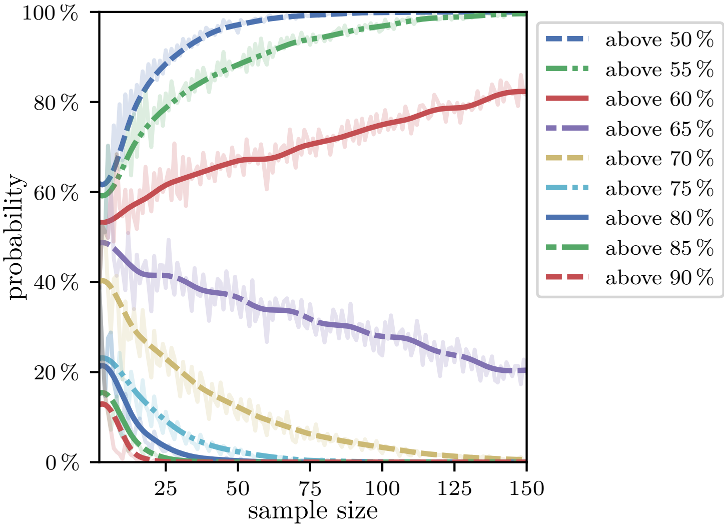

(a) Probabilities for linear SVM to yield an accuracy exceeding a certain threshold as a function of test sample size.

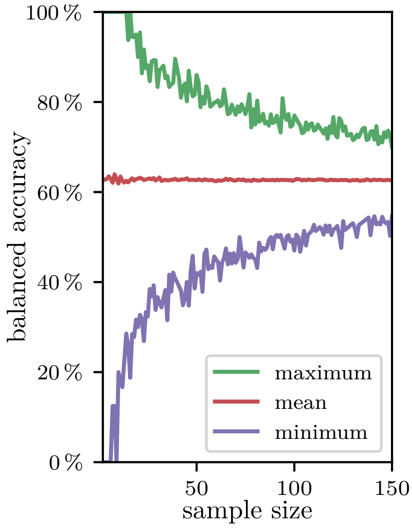

(b) Minimum, maximum and mean results for the linear SVM as a function of test sample size.

Figure F.26: Results as a function of variable test set sizes with a fixed classifier. To reduce the dimensionality of the feature space a **PCA** was performed and 50 **components** were retained. Following, a **linear SVM** was trained with default parameters. ($C = 1.0$)

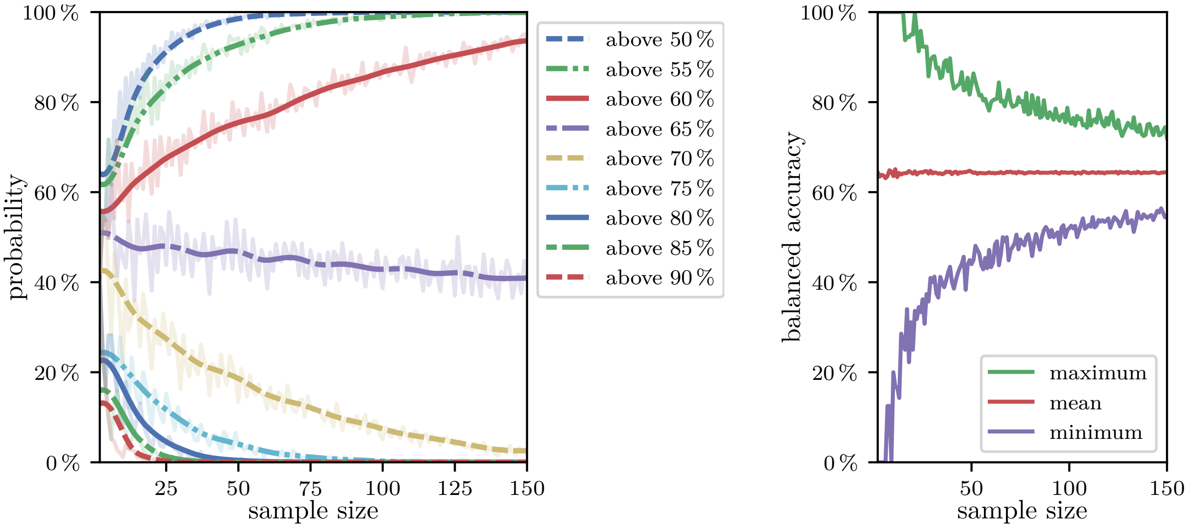

(a) Probabilities for Random Forest to yield an accuracy exceeding a certain threshold as a function of test sample size.

(b) Minimum, maximum and mean results for the Random Forest as a function of test sample size.

Figure F.27: Results as a function of variable test set sizes with a fixed classifier. To reduce the dimensionality of the feature space a **PCA** was performed and 50 **components** were retained. For **feature selection** an ANOVA was computed inside the the pipeline and the top 10 **features** were taken based on the ANOVA F-values. Following, a **Random Forest** was trained with default parameters. ($n_{\text{estimators}} = 100$)

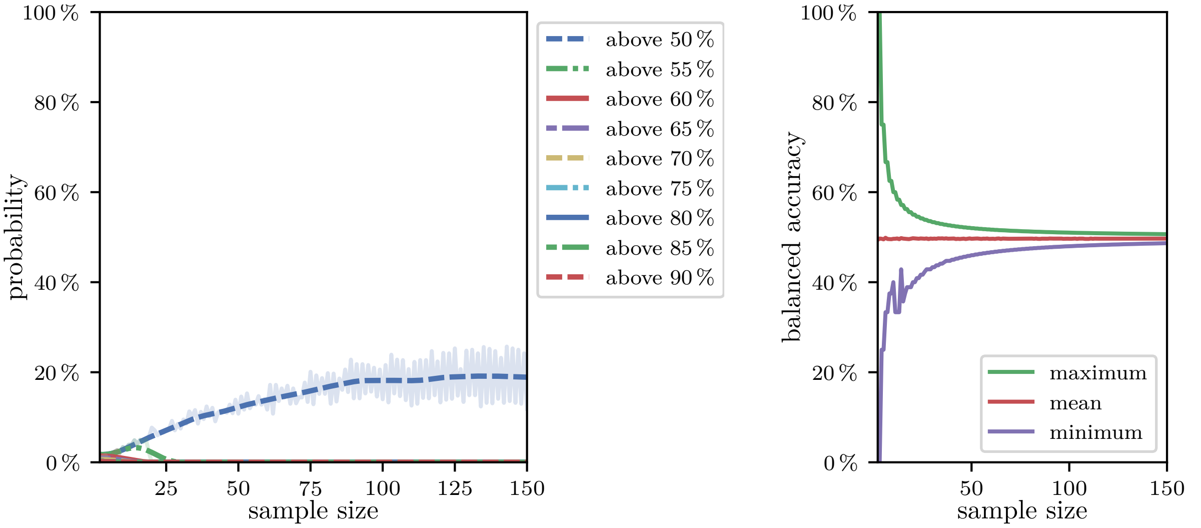

(a) Probabilities for SVM with an RBF kernel to yield an accuracy exceeding a certain threshold as a function of test sample size.

(b) Minimum, maximum and mean results for the SVM with an RBF kernel as a function of test sample size.

Figure F.28: Results as a function of variable test set sizes with a fixed classifier. To reduce the dimensionality of the feature space a **PCA** was performed and 50 **components** were retained. For **feature selection** an ANOVA was computed inside the the pipeline and the top 10 **features** were taken based on the ANOVA F-values. Following, an **SVM** with an **RBF kernel** was trained with default parameters. ($C = 1.0$; $\gamma = {}^{1}/{n_{\text{feature}}}$)

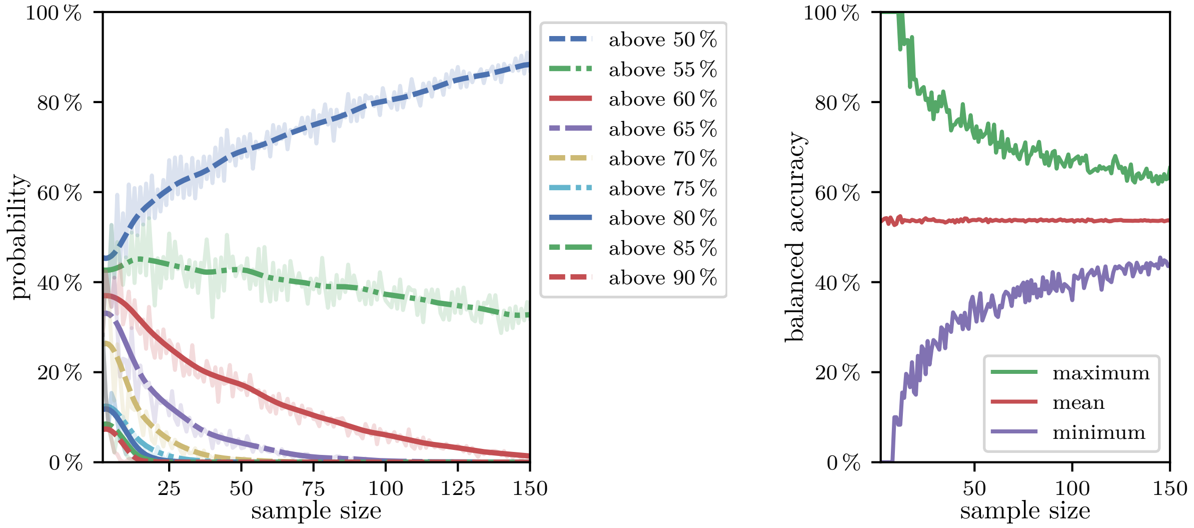

(a) Probabilities for linear SVM to yield an accuracy exceeding a certain threshold as a function of test sample size.

(b) Minimum, maximum and mean results for the linear SVM as a function of test sample size.

Figure F.29: Results as a function of variable test set sizes with a fixed classifier. To reduce the dimensionality of the feature space a **PCA** was performed and 50 **components** were retained. For **feature selection** an ANOVA was computed inside the the pipeline and the top 10 **features** were taken based on the ANOVA F-values. Following, a **linear SVM** was trained with default parameters. $(C = 1.0)$

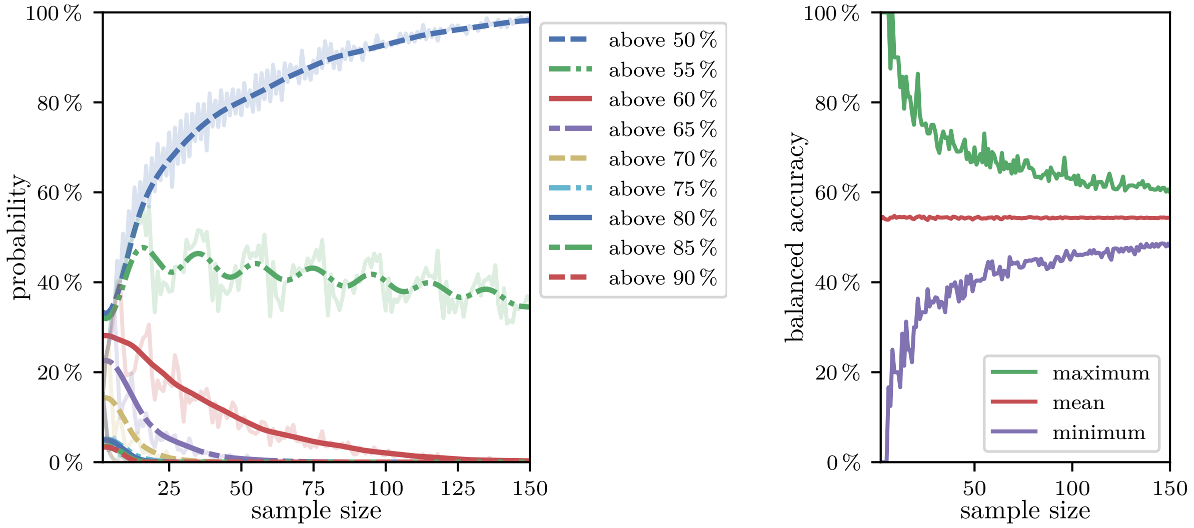

(a) Probabilities for Random Forest to yield an accuracy exceeding a certain threshold as a function of test sample size.

(b) Minimum, maximum and mean results for the Random Forest as a function of test sample size.

Figure F.30: Results as a function of variable test set sizes with a fixed classifier. To reduce the dimensionality of the feature space a **PCA** was performed and 500 **components** were retained. Following, a **Random Forest** was trained with default parameters. $(n_{\text{estimators}} = 100)$

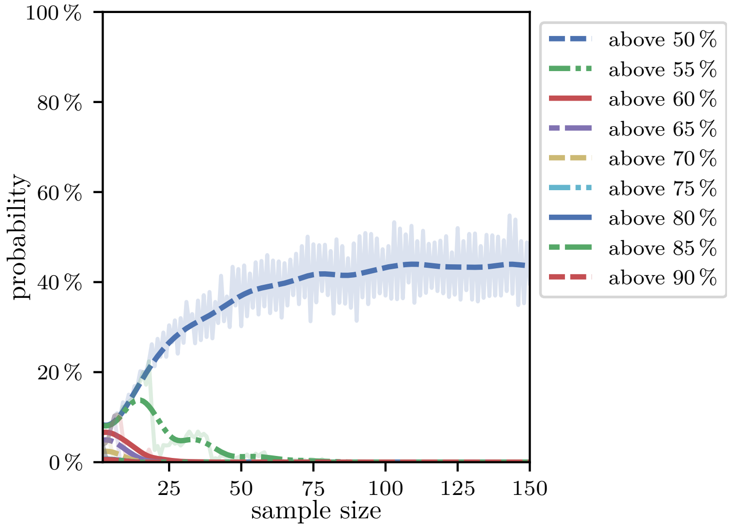

(a) Probabilities for SVM with an RBF kernel to yield an accuracy exceeding a certain threshold as a function of test sample size.

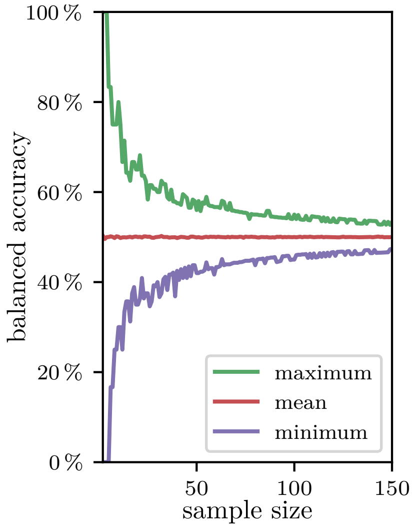

(b) Minimum, maximum and mean results for the SVM with an RBF kernel as a function of test sample size.

Figure F.31: Results as a function of variable test set sizes with a fixed classifier. To reduce the dimensionality of the feature space a **PCA** was performed and 500 **components** were retained. Following, an **SVM** with an **RBF kernel** was trained with default parameters. ($C = 1.0$; $\gamma = {}^{1}/_{n_{\text{feature}}}$)

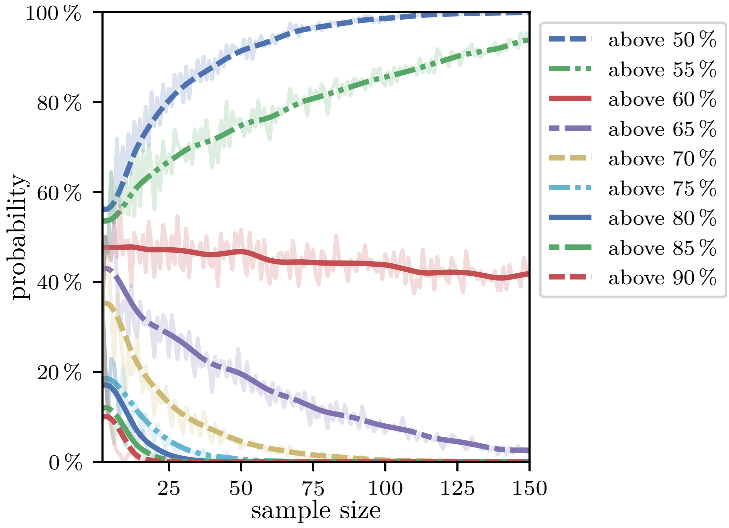

(a) Probabilities for linear SVM to yield an accuracy exceeding a certain threshold as a function of test sample size.

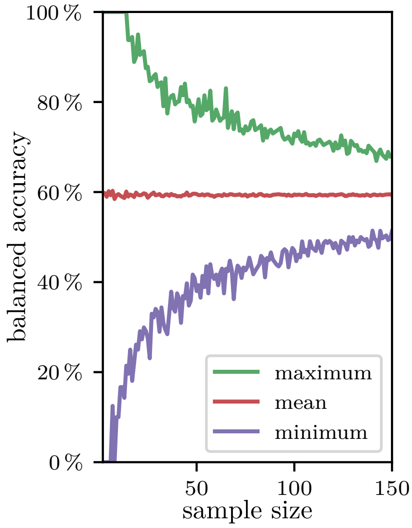

(b) Minimum, maximum and mean results for the linear SVM as a function of test sample size.

Figure F.32: Results as a function of variable test set sizes with a fixed classifier. To reduce the dimensionality of the feature space a **PCA** was performed and 500 **components** were retained. Following, a **linear SVM** was trained with default parameters. ($C = 1.0$)

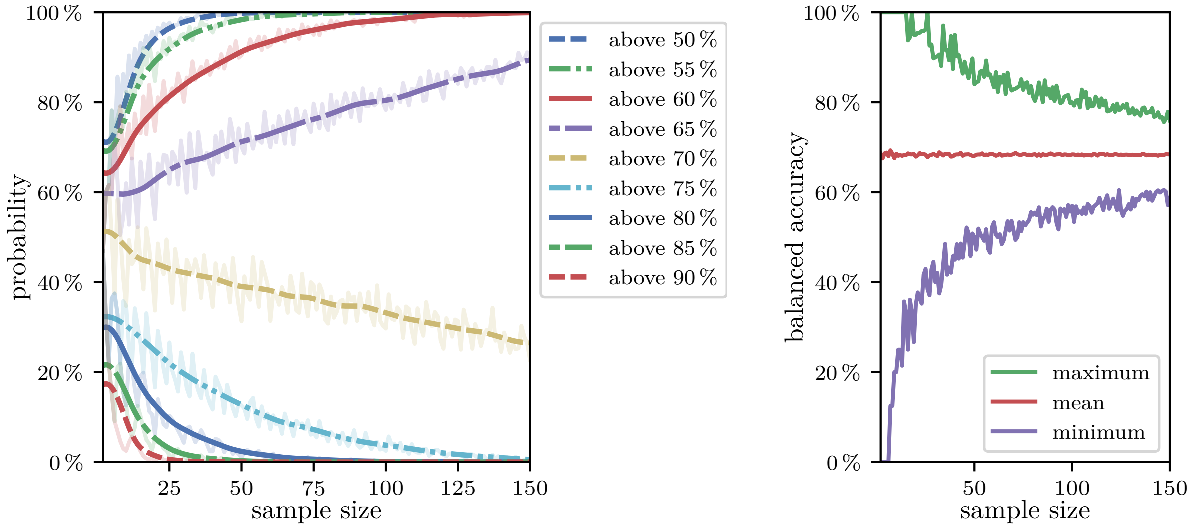

(a) Probabilities for Random Forest to yield an accuracy exceeding a certain threshold as a function of test sample size.

(b) Minimum, maximum and mean results for the Random Forest as a function of test sample size.

Figure F.33: Results as a function of variable test set sizes with a fixed classifier. To reduce the dimensionality of the feature space a **PCA** was performed and 500 **components** were retained. For **feature selection** an ANOVA was computed inside the the pipeline and the top 10 **features** were taken based on the ANOVA F-values. Following, a **Random Forest** was trained with default parameters. ($n_{\text{estimators}} = 100$)

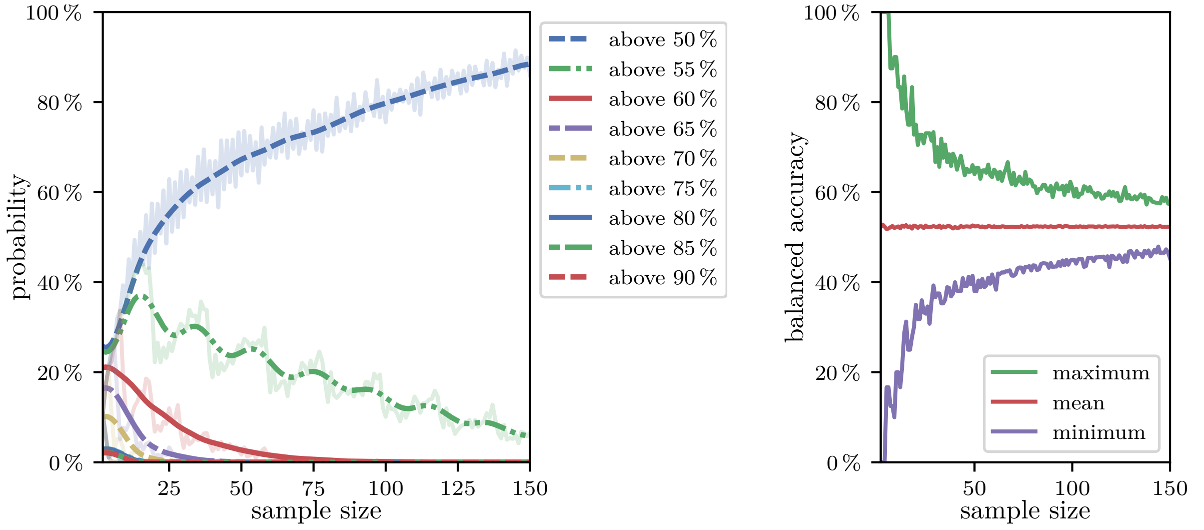

(a) Probabilities for SVM with an RBF kernel to yield an accuracy exceeding a certain threshold as a function of test sample size.

(b) Minimum, maximum and mean results for the SVM with an RBF kernel as a function of test sample size.

Figure F.34: Results as a function of variable test set sizes with a fixed classifier. To reduce the dimensionality of the feature space a **PCA** was performed and 500 **components** were retained. For **feature selection** an ANOVA was computed inside the the pipeline and the top 10 **features** were taken based on the ANOVA F-values. Following, an **SVM** with an **RBF kernel** was trained with default parameters. ($C = 1.0$; $\gamma = {}^{1}/{n_{\text{feature}}}$)

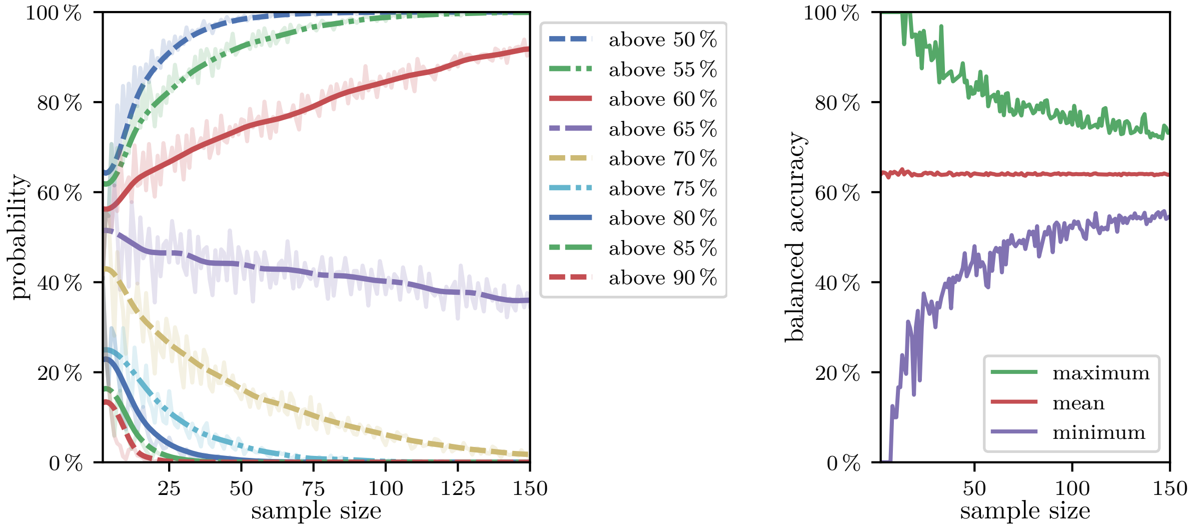

(a) Probabilities for linear SVM to yield an accuracy exceeding a certain threshold as a function of test sample size.

(b) Minimum, maximum and mean results for the linear SVM as a function of test sample size.

Figure F.35: Results as a function of variable test set sizes with a fixed classifier. To reduce the dimensionality of the feature space a **PCA** was performed and 500 **components** were retained. For **feature selection** an ANOVA was computed inside the the pipeline and the top 10 **features** were taken based on the ANOVA F-values. Following, a **linear SVM** was trained with default parameters. ($C = 1.0$)

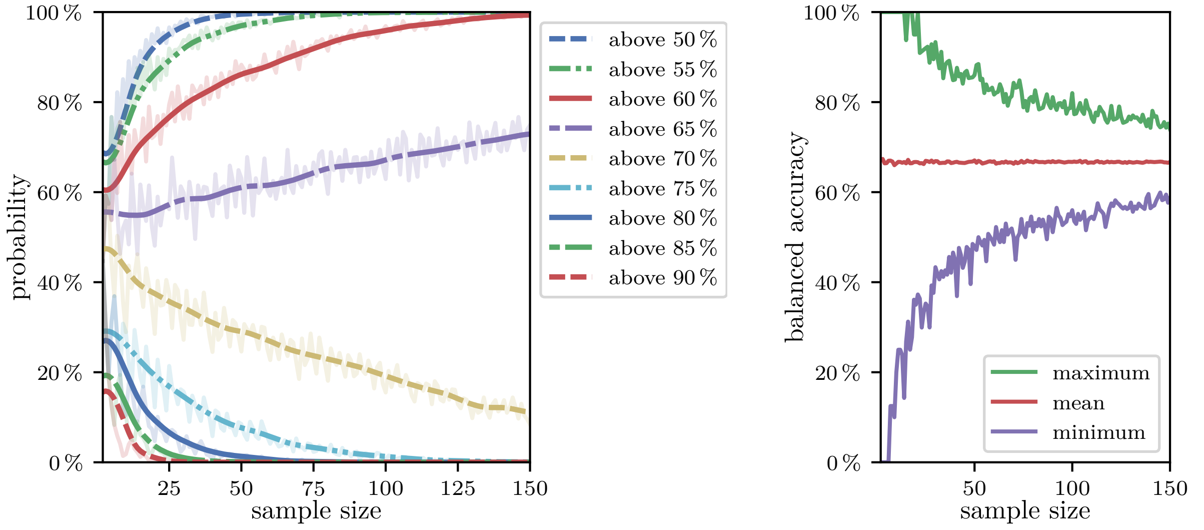

(a) Probabilities for Random Forest to yield an accuracy exceeding a certain threshold as a function of test sample size.

(b) Minimum, maximum and mean results for the Random Forest as a function of test sample size.

Figure F.36: Results as a function of variable test set sizes with a fixed classifier. To reduce the dimensionality of the feature space a **PCA** was performed and 500 **components** were retained. For **feature selection** an ANOVA was computed inside the the pipeline and the top 100 **features** were taken based on the ANOVA F-values. Following, a **Random Forest** was trained with default parameters. ($n_{\text{estimators}} = 100$)

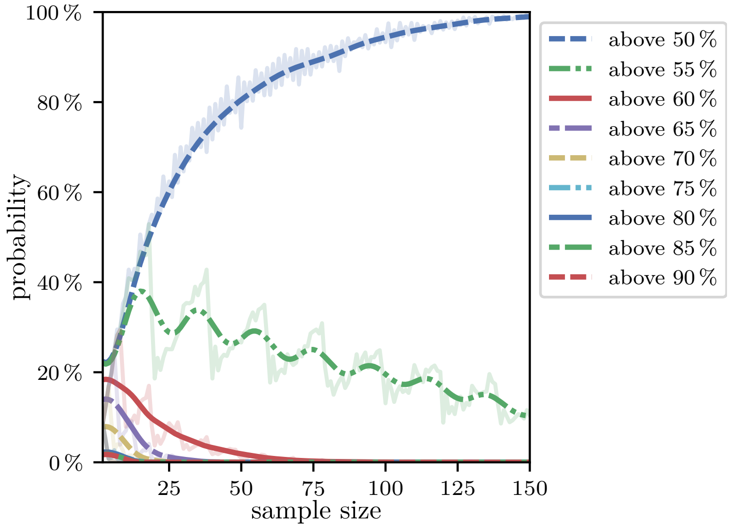

(a) Probabilities for SVM with an RBF kernel to yield an accuracy exceeding a certain threshold as a function of test sample size.

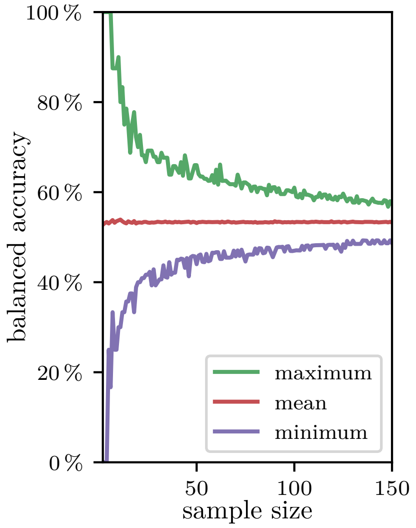

(b) Minimum, maximum and mean results for the SVM with an RBF kernel as a function of test sample size.

Figure F.37: Results as a function of variable test set sizes with a fixed classifier. To reduce the dimensionality of the feature space a **PCA** was performed and 500 **components** were retained. For **feature selection** an ANOVA was computed inside the the pipeline and the top 100 **features** were taken based on the ANOVA F-values. Following, an **SVM** with an **RBF kernel** was trained with default parameters. ($C = 1.0$; $\gamma = {}^{1}/{n_{\text{feature}}}$)

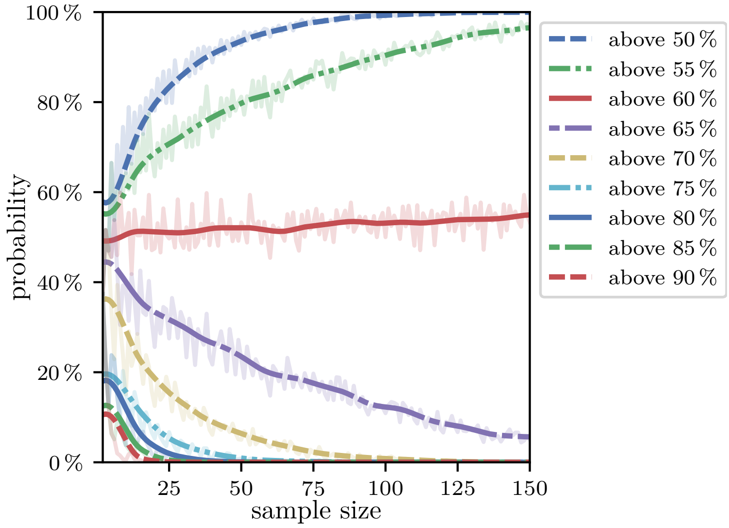

(a) Probabilities for linear SVM to yield an accuracy exceeding a certain threshold as a function of test sample size.

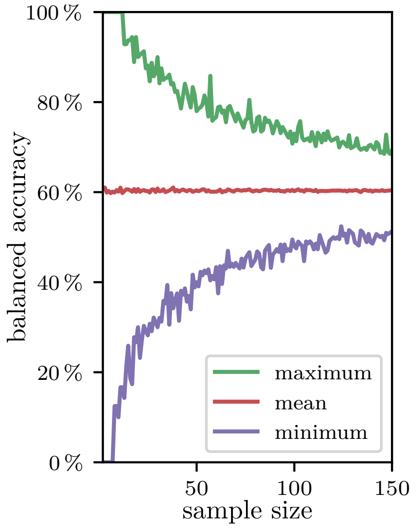

(b) Minimum, maximum and mean results for the linear SVM as a function of test sample size.

Figure F.38: Results as a function of variable test set sizes with a fixed classifier. To reduce the dimensionality of the feature space a **PCA** was performed and 500 **components** were retained. For **feature selection** an ANOVA was computed inside the the pipeline and the top 100 **features** were taken based on the ANOVA F-values. Following, a **linear SVM** was trained with default parameters. ($C = 1.0$)

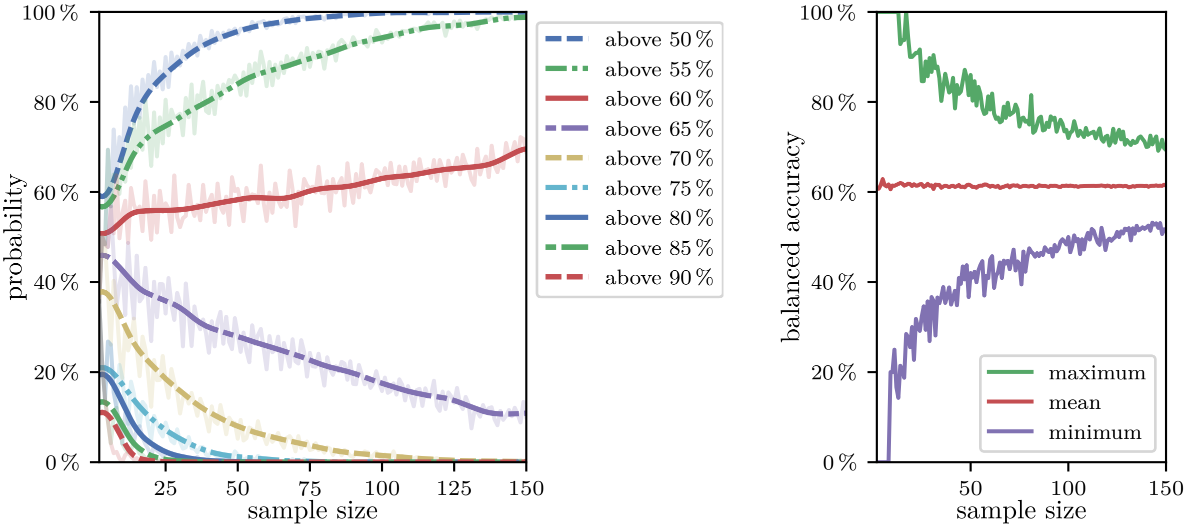

(a) Probabilities for Random Forest to yield an accuracy exceeding a certain threshold as a function of test sample size.

(b) Minimum, maximum and mean results for the Random Forest as a function of test sample size.

Figure F.39: Results as a function of variable test set sizes with a fixed classifier. To reduce the dimensionality of the feature space a **PCA** was performed and **all components** were retained. Following, a **Random Forest** was trained with default parameters. ($n_{\text{estimators}} = 100$)

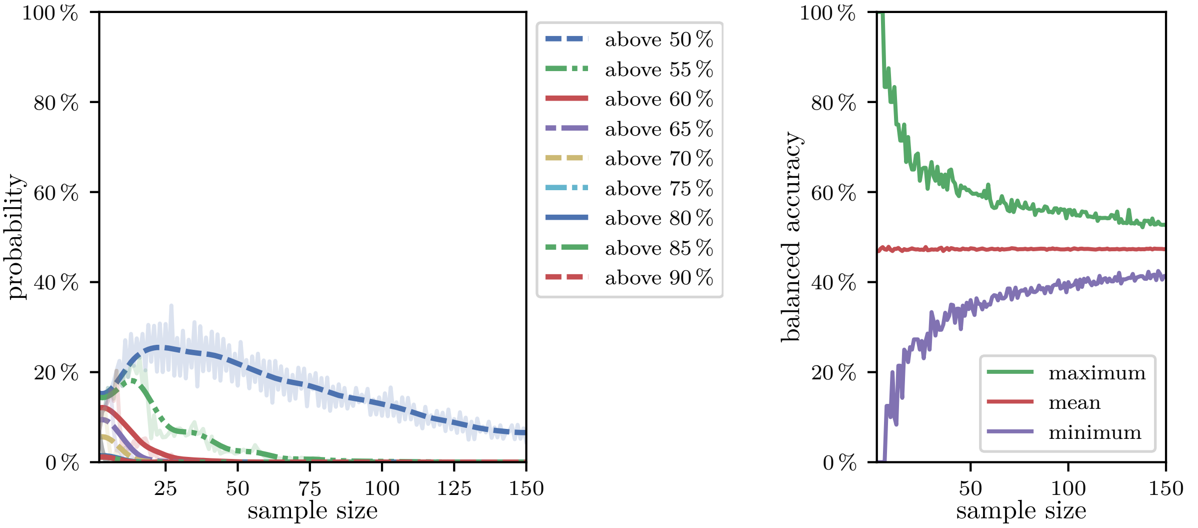

(a) Probabilities for SVM with an RBF kernel to yield an accuracy exceeding a certain threshold as a function of test sample size.

(b) Minimum, maximum and mean results for the SVM with an RBF kernel as a function of test sample size.

Figure F.40: Results as a function of variable test set sizes with a fixed classifier. To reduce the dimensionality of the feature space a **PCA** was performed and **all components** were retained. Following, an **SVM** with an **RBF kernel** was trained with default parameters. ($C = 1.0$; $\gamma = {}^1/_{n_{\text{feature}}}$)

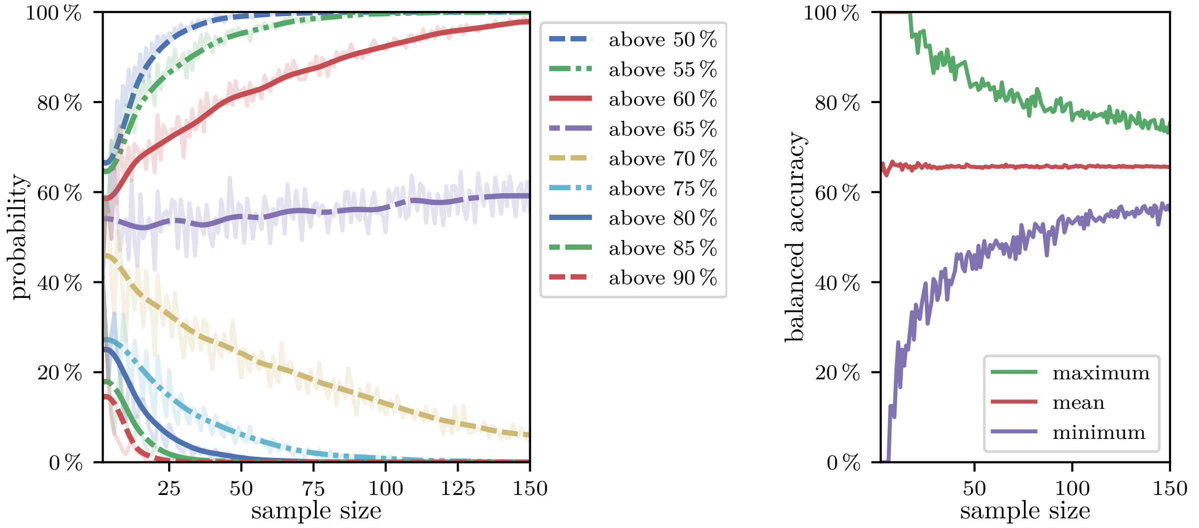

(a) Probabilities for linear SVM to yield an accuracy exceeding a certain threshold as a function of test sample size.

(b) Minimum, maximum and mean results for the linear SVM as a function of test sample size.

Figure F.41: Results as a function of variable test set sizes with a fixed classifier. To reduce the dimensionality of the feature space a **PCA** was performed and **all components** were retained. Following, a **linear SVM** was trained with default parameters. ($C = 1.0$)

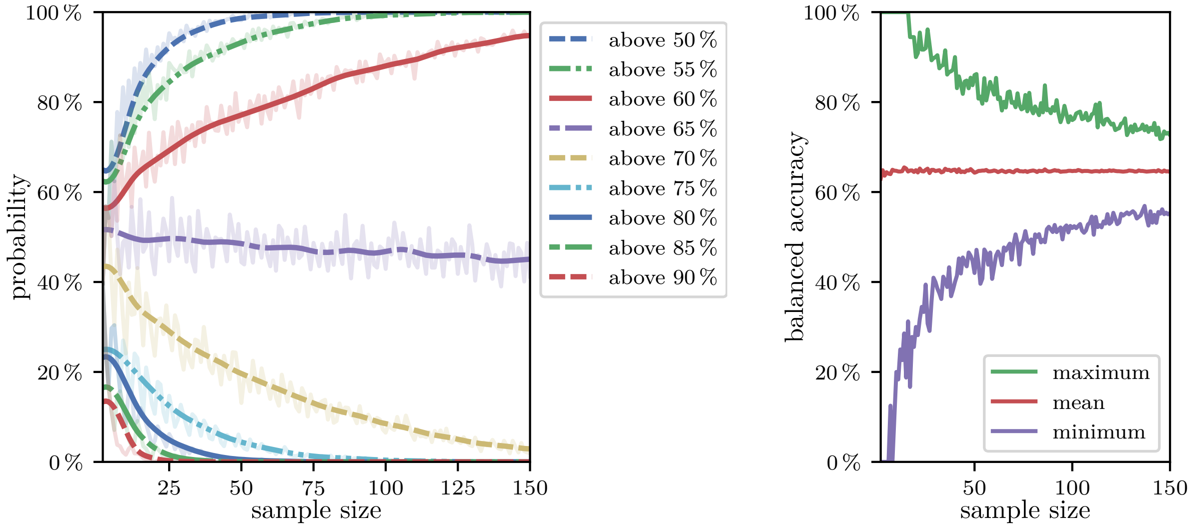

(a) Probabilities for Random Forest to yield an accuracy exceeding a certain threshold as a function of test sample size.

(b) Minimum, maximum and mean results for the Random Forest as a function of test sample size.

Figure F.42: Results as a function of variable test set sizes with a fixed classifier. To reduce the dimensionality of the feature space a **PCA** was performed and **all components** were retained. For **feature selection** an ANOVA was computed inside the the pipeline and the top 10 **features** were taken based on the ANOVA F-values. Following, a **Random Forest** was trained with default parameters. ($n_{\text{estimators}} = 100$)

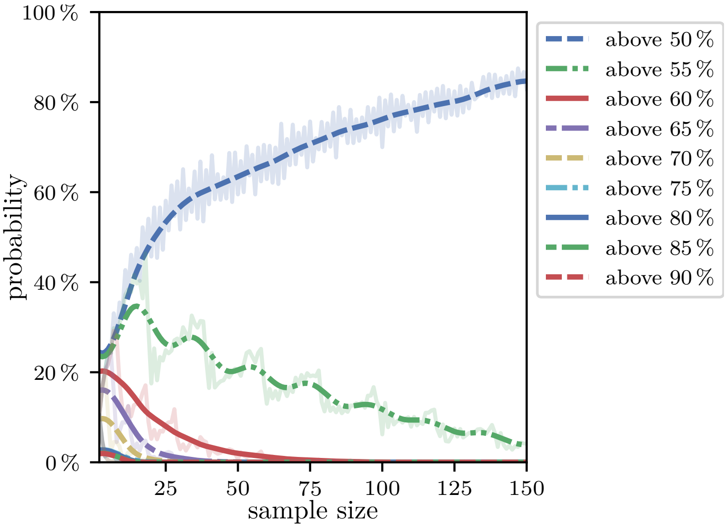

(a) Probabilities for SVM with an RBF kernel to yield an accuracy exceeding a certain threshold as a function of test sample size.

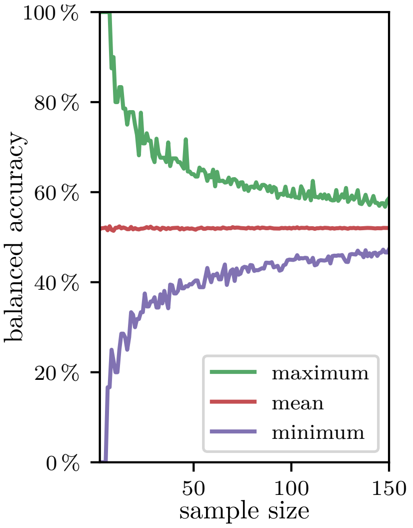

(b) Minimum, maximum and mean results for the SVM with an RBF kernel as a function of test sample size.

Figure F.43: Results as a function of variable test set sizes with a fixed classifier. To reduce the dimensionality of the feature space a **PCA** was performed and **all components** were retained. For **feature selection** an ANOVA was computed inside the the pipeline and the top 10 **features** were taken based on the ANOVA F-values. Following, an **SVM** with an **RBF kernel** was trained with default parameters. ($C = 1.0$; $\gamma = {}^{1}/{n_{\text{feature}}}$)

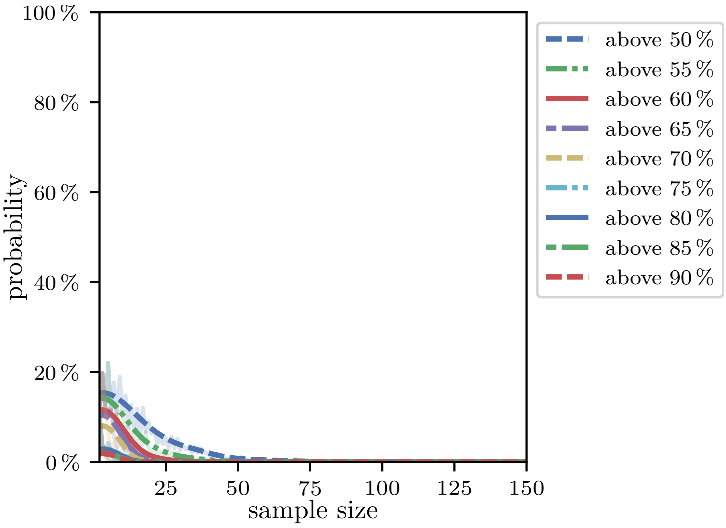

(a) Probabilities for linear SVM to yield an accuracy exceeding a certain threshold as a function of test sample size.

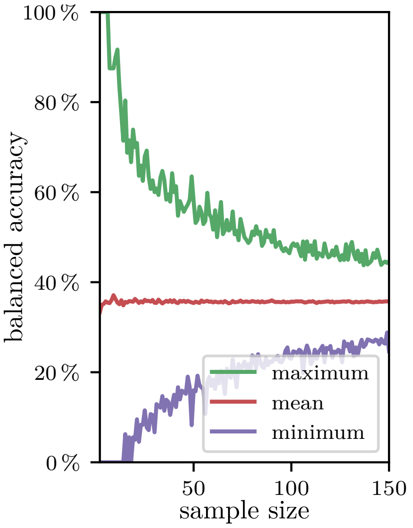

(b) Minimum, maximum and mean results for the linear SVM as a function of test sample size.

Figure F.44: Results as a function of variable test set sizes with a fixed classifier. To reduce the dimensionality of the feature space a **PCA** was performed and **all components** were retained. For **feature selection** an ANOVA was computed inside the the pipeline and the top 10 **features** were taken based on the ANOVA F-values. Following, a **linear SVM** was trained with default parameters. ($C = 1.0$)

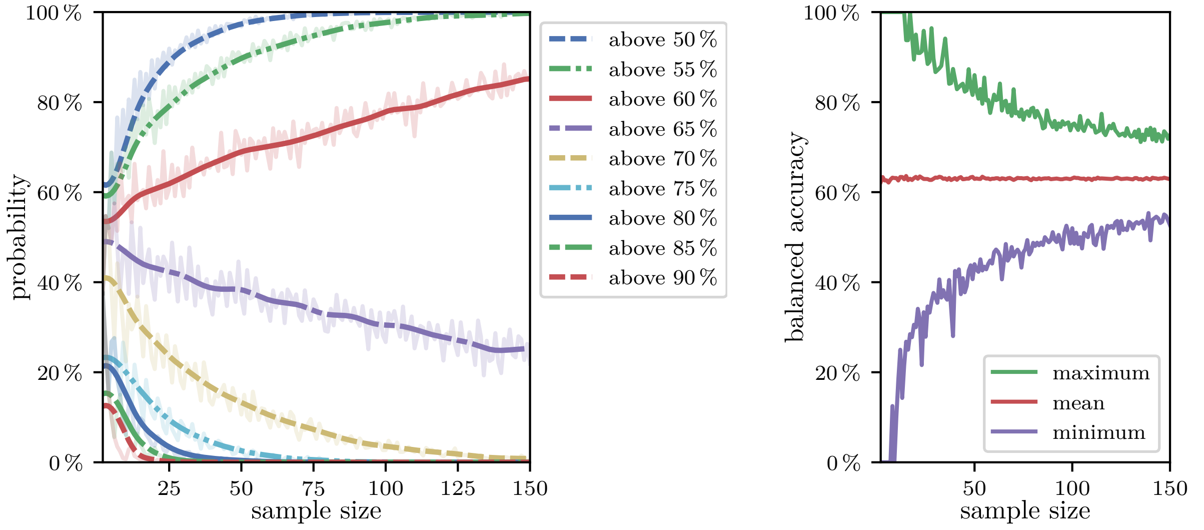

(a) Probabilities for Random Forest to yield an accuracy exceeding a certain threshold as a function of test sample size.

(b) Minimum, maximum and mean results for the Random Forest as a function of test sample size.

Figure F.45: Results as a function of variable test set sizes with a fixed classifier. To reduce the dimensionality of the feature space a **PCA** was performed and **all components** were retained. For **feature selection** an ANOVA was computed inside the the pipeline and the top 100 **features** were taken based on the ANOVA F-values. Following, a **Random Forest** was trained with default parameters. ($n_{\text{estimators}} = 100$)

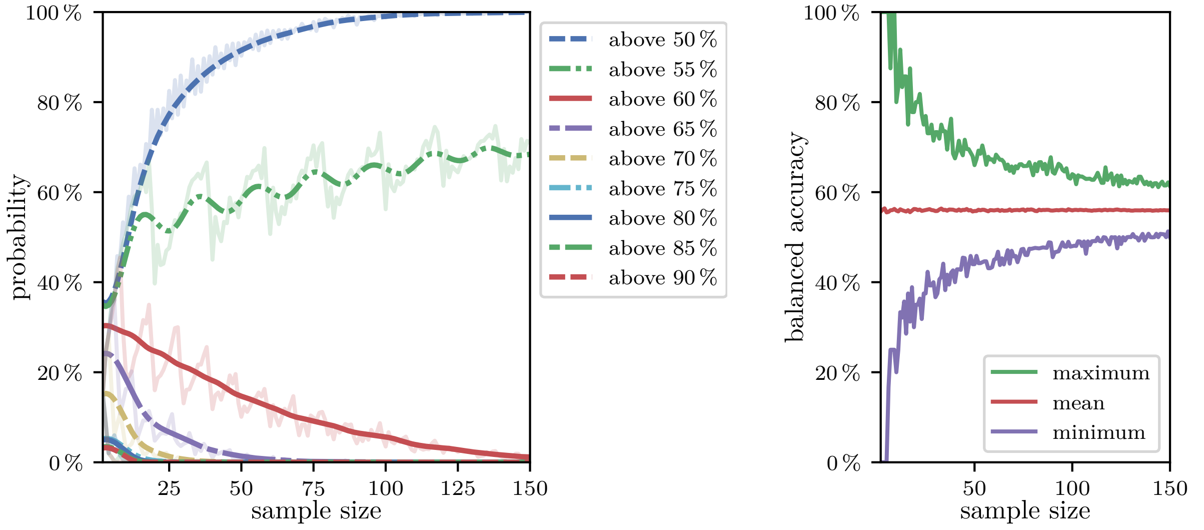

(a) Probabilities for SVM with an RBF kernel to yield an accuracy exceeding a certain threshold as a function of test sample size.

(b) Minimum, maximum and mean results for the SVM with an RBF kernel as a function of test sample size.

Figure F.46: Results as a function of variable test set sizes with a fixed classifier. To reduce the dimensionality of the feature space a **PCA** was performed and **all components** were retained. For **feature selection** an ANOVA was computed inside the the pipeline and the top 100 **features** were taken based on the ANOVA F-values. Following, an **SVM** with an **RBF kernel** was trained with default parameters. ($C = 1.0$; $\gamma = {}^{1}/_{n_{\text{feature}}}$)

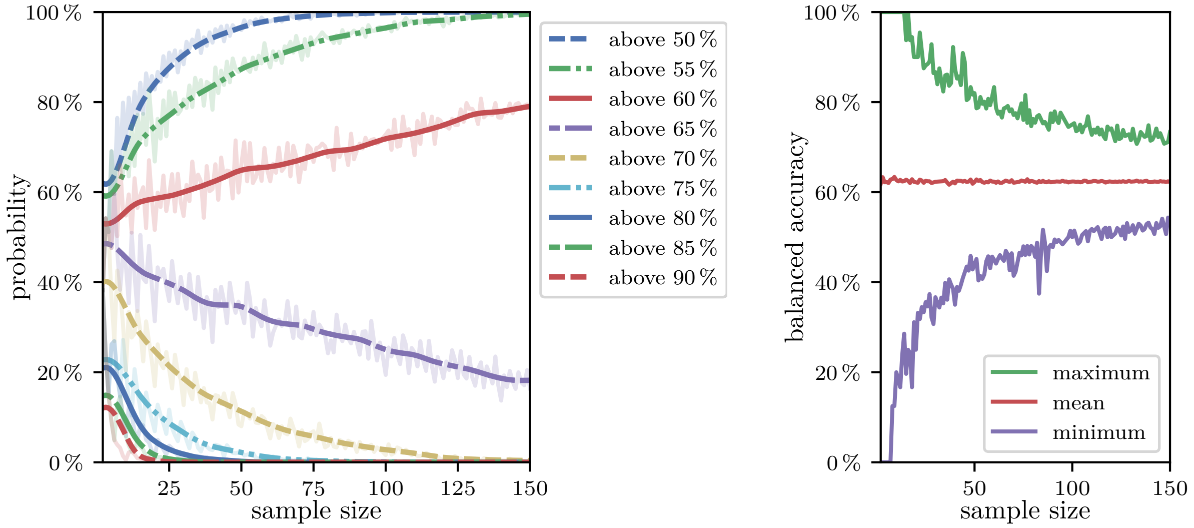

(a) Probabilities for linear SVM to yield an accuracy exceeding a certain threshold as a function of test sample size.

(b) Minimum, maximum and mean results for the linear SVM as a function of test sample size.

Figure F.47: Results as a function of variable test set sizes with a fixed classifier. To reduce the dimensionality of the feature space a **PCA** was performed and **all components** were retained. For **feature selection** an ANOVA was computed inside the the pipeline and the top 100 **features** were taken based on the ANOVA F-values. Following, a **linear SVM** was trained with default parameters. ($C = 1.0$)

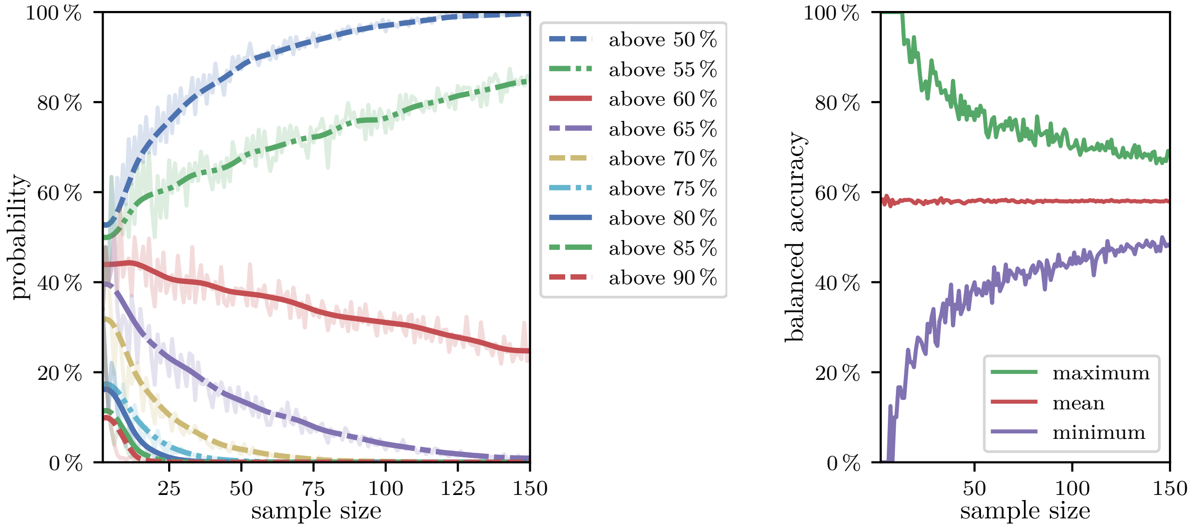

(a) Probabilities for Random Forest to yield an accuracy exceeding a certain threshold as a function of test sample size.

(b) Minimum, maximum and mean results for the Random Forest as a function of test sample size.

Figure F.48: Results as a function of variable test set sizes with a fixed classifier. To reduce the dimensionality of the feature space a **PCA** was performed and **all components** were retained. For **feature selection** an ANOVA was computed inside the the pipeline and the top 1,000 **features** were taken based on the ANOVA F-values. Following, a **Random Forest** was trained with default parameters. ($n_{\text{estimators}} = 100$)

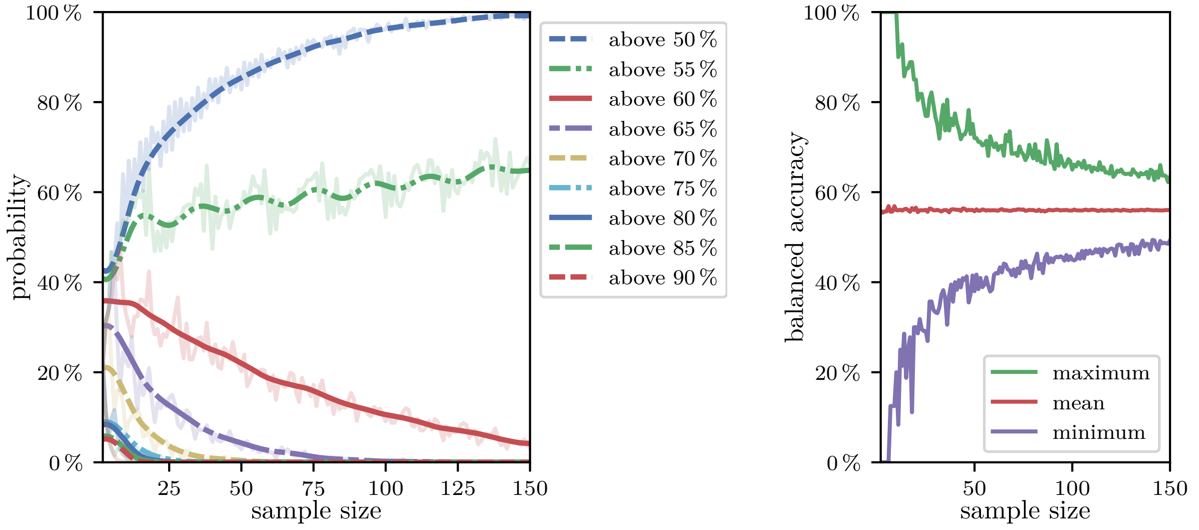

(a) Probabilities for SVM with an RBF kernel to yield an accuracy exceeding a certain threshold as a function of test sample size.

(b) Minimum, maximum and mean results for the SVM with an RBF kernel as a function of test sample size.

Figure F.49: Results as a function of variable test set sizes with a fixed classifier. To reduce the dimensionality of the feature space a **PCA** was performed and **all components** were retained. For **feature selection** an ANOVA was computed inside the the pipeline and the top 1,000 **features** were taken based on the ANOVA F-values. Following, an **SVM** with an **RBF kernel** was trained with default parameters. ($C = 1.0$; $\gamma = {}^{1}/{n_{\text{feature}}}$)

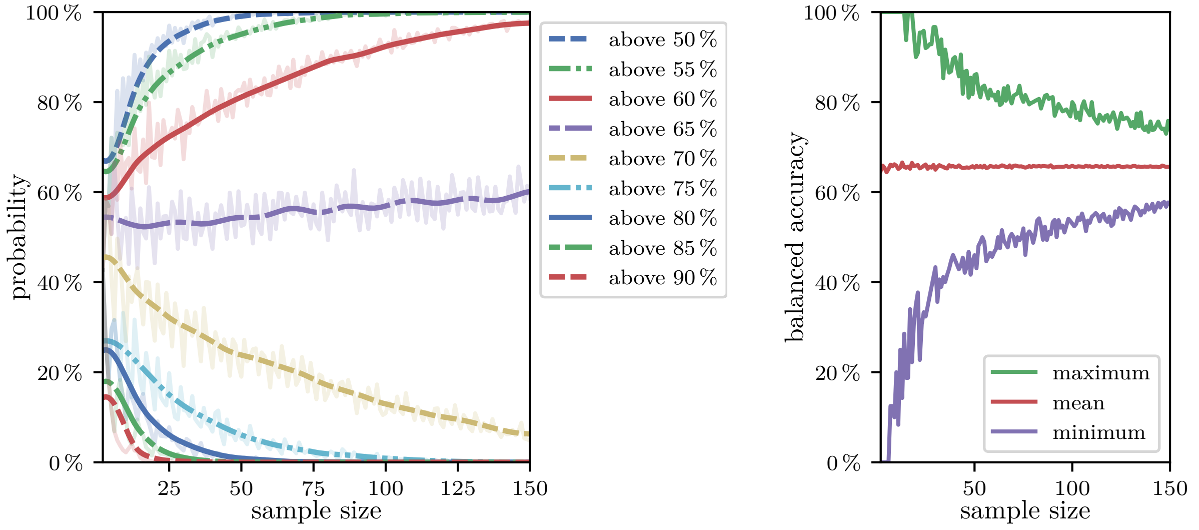

(a) Probabilities for linear SVM to yield an accuracy exceeding a certain threshold as a function of test sample size.

(b) Minimum, maximum and mean results for the linear SVM as a function of test sample size.

Figure F.50: Results as a function of variable test set sizes with a fixed classifier. To reduce the dimensionality of the feature space a **PCA** was performed and **all components** were retained. For **feature selection** an ANOVA was computed inside the the pipeline and the top 1,000 **features** were taken based on the ANOVA F-values. Following, a **linear SVM** was trained with default parameters. ($C = 1.0$)

Appendix F.1.2. Tabular results representation

	n	≥ **accuracy** 50	55	60	65	70	75	80	85	90
**test sample size**	10	0.79	0.79	0.65	0.54	0.28	0.28	0.08	0.08	0.01
	20	0.92	0.81	0.67	0.47	0.29	0.15	0.07	0.06	0.00
	30	0.96	0.91	0.74	0.58	0.29	0.15	0.03	0.01	0.00
	40	0.98	0.93	0.80	0.50	0.24	0.07	0.01	0.00	0.00
	50	0.99	0.96	0.86	0.59	0.23	0.08	0.00	0.00	0.00
	60	1.00	0.98	0.87	0.55	0.23	0.03	0.00	0.00	0.00
	70	1.00	0.99	0.89	0.61	0.21	0.03	0.00	0.00	0.00
	80	1.00	0.99	0.90	0.54	0.16	0.02	0.00	0.00	0.00
	90	1.00	1.00	0.93	0.61	0.14	0.02	0.00	0.00	0.00
	100	1.00	1.00	0.94	0.59	0.14	0.01	0.00	0.00	0.00
	125	1.00	1.00	0.96	0.64	0.12	0.01	0.00	0.00	0.00
	150	1.00	1.00	0.99	0.66	0.07	0.00	0.00	0.00	0.00

(a) Probabilities to yield an accuracy exceeding a certain threshold related to the test sample size.

	n	**mean**	**median**	**min**	**max**	**std**
**test sample size**	10	0.66	0.70	0.20	1.00	0.14
	20	0.67	0.65	0.35	0.95	0.10
	30	0.67	0.67	0.37	0.90	0.08
	40	0.66	0.65	0.42	0.85	0.07
	50	0.66	0.66	0.46	0.84	0.06
	60	0.67	0.67	0.50	0.80	0.05
	70	0.67	0.67	0.51	0.81	0.05
	80	0.66	0.66	0.53	0.81	0.05
	90	0.66	0.67	0.51	0.78	0.04
	100	0.66	0.66	0.55	0.77	0.04
	125	0.66	0.66	0.54	0.76	0.03
	150	0.66	0.66	0.57	0.75	0.03

(b) Summary of the accuracy value distributions for certain sample sizes.

Table F.11: Results as a function of variable test set sizes with a fixed classifier. A **Random Forest** was trained with default parameters. ($n_{\text{estimators}} = 100$)

test sample size: n	≥ accuracy: 50	55	60	65	70	75	80	85	90
10	0.79	0.79	0.65	0.55	0.29	0.29	0.10	0.10	0.01
20	0.91	0.80	0.65	0.45	0.25	0.12	0.04	0.03	0.00
30	0.96	0.91	0.71	0.56	0.26	0.14	0.03	0.01	0.00
40	0.98	0.91	0.79	0.48	0.21	0.04	0.01	0.00	0.00
50	0.99	0.96	0.84	0.55	0.20	0.07	0.00	0.00	0.00
60	0.99	0.97	0.83	0.49	0.19	0.04	0.00	0.00	0.00
70	1.00	0.99	0.86	0.60	0.16	0.03	0.00	0.00	0.00
80	1.00	0.99	0.91	0.53	0.15	0.02	0.00	0.00	0.00
90	1.00	0.99	0.92	0.62	0.14	0.02	0.00	0.00	0.00
100	1.00	1.00	0.95	0.57	0.13	0.00	0.00	0.00	0.00
125	1.00	1.00	0.96	0.63	0.12	0.01	0.00	0.00	0.00
150	1.00	1.00	0.98	0.64	0.05	0.00	0.00	0.00	0.00

(a) Probabilities to yield an accuracy exceeding a certain threshold related to the test sample size.

test sample size: n	**mean**	**median**	**min**	**max**	**std**
10	0.66	0.70	0.20	1.00	0.15
20	0.66	0.65	0.30	0.95	0.10
30	0.66	0.67	0.40	0.90	0.08
40	0.66	0.65	0.45	0.93	0.07
50	0.66	0.66	0.46	0.86	0.06
60	0.66	0.65	0.50	0.82	0.05
70	0.66	0.66	0.50	0.79	0.05
80	0.66	0.66	0.53	0.84	0.04
90	0.66	0.67	0.50	0.80	0.04
100	0.66	0.66	0.54	0.77	0.04
125	0.66	0.66	0.56	0.78	0.03
150	0.66	0.66	0.59	0.75	0.03

(b) Summary of the accuracy value distributions for certain sample sizes.

Table F.12: Results as a function of variable test set sizes with a fixed classifier. An **SVM** with an **RBF kernel** was trained with default parameters. ($C = 1.0$; $\gamma = {}^{1}/{n_{\text{feature}}}$)

		≥ **accuracy**								
	n	50	55	60	65	70	75	80	85	90
**test sample size**	10	0.77	0.77	0.63	0.53	0.27	0.27	0.08	0.08	0.01
	20	0.90	0.78	0.65	0.46	0.27	0.14	0.05	0.04	0.00
	30	0.95	0.89	0.69	0.54	0.23	0.12	0.02	0.01	0.00
	40	0.98	0.90	0.78	0.45	0.19	0.06	0.01	0.00	0.00
	50	1.00	0.97	0.83	0.55	0.18	0.06	0.01	0.00	0.00
	60	1.00	0.96	0.82	0.51	0.18	0.02	0.00	0.00	0.00
	70	1.00	0.98	0.84	0.55	0.16	0.03	0.00	0.00	0.00
	80	1.00	0.99	0.89	0.52	0.16	0.01	0.00	0.00	0.00
	90	1.00	1.00	0.89	0.57	0.12	0.01	0.00	0.00	0.00
	100	1.00	1.00	0.93	0.52	0.10	0.00	0.00	0.00	0.00
	125	1.00	1.00	0.96	0.58	0.10	0.00	0.00	0.00	0.00
	150	1.00	1.00	0.98	0.61	0.04	0.00	0.00	0.00	0.00

(a) Probabilities to yield an accuracy exceeding a certain threshold related to the test sample size.

	n	**mean**	**median**	**min**	**max**	**std**
**test sample size**	10	0.65	0.70	0.20	1.00	0.15
	20	0.66	0.65	0.30	0.95	0.11
	30	0.66	0.67	0.43	1.00	0.08
	40	0.65	0.65	0.38	0.90	0.07
	50	0.66	0.66	0.46	0.86	0.06
	60	0.66	0.67	0.48	0.82	0.05
	70	0.66	0.66	0.50	0.80	0.05
	80	0.66	0.66	0.50	0.80	0.05
	90	0.66	0.66	0.53	0.78	0.04
	100	0.66	0.66	0.52	0.79	0.04
	125	0.66	0.66	0.54	0.76	0.03
	150	0.66	0.66	0.57	0.75	0.03

(b) Summary of the accuracy value distributions for certain sample sizes.

Table F.13: Results as a function of variable test set sizes with a fixed classifier. A **linear SVM** was trained with default parameters. ($C = 1.0$)

test sample size n	≥ accuracy 50	55	60	65	70	75	80	85	90
10	0.67	0.67	0.52	0.41	0.18	0.18	0.06	0.06	0.01
20	0.81	0.64	0.49	0.28	0.13	0.05	0.01	0.01	0.00
30	0.87	0.77	0.49	0.34	0.12	0.05	0.01	0.00	0.00
40	0.93	0.78	0.60	0.25	0.07	0.02	0.00	0.00	0.00
50	0.95	0.84	0.60	0.28	0.06	0.01	0.00	0.00	0.00
60	0.97	0.84	0.51	0.20	0.05	0.00	0.00	0.00	0.00
70	0.98	0.88	0.50	0.23	0.04	0.00	0.00	0.00	0.00
80	0.99	0.87	0.58	0.17	0.02	0.00	0.00	0.00	0.00
90	0.99	0.93	0.62	0.21	0.02	0.00	0.00	0.00	0.00
100	0.99	0.93	0.63	0.15	0.01	0.00	0.00	0.00	0.00
125	1.00	0.98	0.64	0.14	0.01	0.00	0.00	0.00	0.00
150	1.00	0.98	0.68	0.09	0.00	0.00	0.00	0.00	0.00

(a) Probabilities to yield an accuracy exceeding a certain threshold related to the test sample size.

test sample size n	**mean**	**median**	**min**	**max**	**std**
10	0.61	0.60	0.10	1.00	0.15
20	0.61	0.60	0.20	0.90	0.10
30	0.61	0.60	0.33	0.87	0.09
40	0.62	0.62	0.40	0.80	0.07
50	0.61	0.62	0.40	0.84	0.06
60	0.61	0.62	0.43	0.78	0.06
70	0.61	0.60	0.43	0.80	0.05
80	0.61	0.61	0.42	0.77	0.05
90	0.61	0.61	0.48	0.76	0.04
100	0.61	0.61	0.49	0.74	0.04
125	0.61	0.62	0.50	0.72	0.03
150	0.61	0.61	0.52	0.70	0.03

(b) Summary of the accuracy value distributions for certain sample sizes.

Table F.14: Results as a function of variable test set sizes with a fixed classifier. For **feature selection** an ANOVA was computed inside the the pipeline and the top 10 **features** were taken based on the ANOVA F-values. Following, a **Random Forest** was trained with default parameters. ($n_{\text{estimators}} = 100$)

test sample size n	≥ accuracy 50	55	60	65	70	75	80	85	90
10	0.77	0.77	0.64	0.54	0.28	0.28	0.09	0.09	0.01
20	0.90	0.80	0.66	0.45	0.26	0.12	0.05	0.04	0.00
30	0.94	0.90	0.67	0.53	0.24	0.13	0.03	0.01	0.00
40	0.98	0.91	0.75	0.45	0.21	0.05	0.01	0.00	0.00
50	0.99	0.96	0.85	0.57	0.21	0.08	0.01	0.00	0.00
60	1.00	0.97	0.82	0.48	0.17	0.03	0.00	0.00	0.00
70	1.00	0.98	0.83	0.55	0.16	0.03	0.00	0.00	0.00
80	1.00	0.98	0.89	0.52	0.13	0.01	0.00	0.00	0.00
90	1.00	0.99	0.90	0.57	0.14	0.01	0.00	0.00	0.00
100	1.00	1.00	0.94	0.51	0.10	0.01	0.00	0.00	0.00
125	1.00	1.00	0.95	0.55	0.09	0.00	0.00	0.00	0.00
150	1.00	1.00	0.98	0.59	0.05	0.00	0.00	0.00	0.00

(a) Probabilities to yield an accuracy exceeding a certain threshold related to the test sample size.

test sample size n	**mean**	**median**	**min**	**max**	**std**
10	0.66	0.70	0.20	1.00	0.15
20	0.66	0.65	0.30	0.95	0.10
30	0.66	0.67	0.37	0.90	0.08
40	0.65	0.65	0.42	0.85	0.07
50	0.66	0.66	0.48	0.84	0.06
60	0.66	0.65	0.50	0.82	0.05
70	0.66	0.66	0.49	0.83	0.05
80	0.66	0.66	0.53	0.80	0.05
90	0.66	0.66	0.52	0.78	0.04
100	0.66	0.66	0.53	0.79	0.04
125	0.66	0.66	0.56	0.76	0.03
150	0.66	0.65	0.57	0.73	0.03

(b) Summary of the accuracy value distributions for certain sample sizes.

Table F.15: Results as a function of variable test set sizes with a fixed classifier. For **feature selection** an ANOVA was computed inside the the pipeline and the top 10 **features** were taken based on the ANOVA F-values. Following, an **SVM** with an **RBF kernel** was trained with default parameters. ($C = 1.0$; $\gamma = {}^{1}/{n_{\text{feature}}}$)

	n	≥ **accuracy** 50	55	60	65	70	75	80	85	90
**test sample size**	10	0.80	0.80	0.69	0.57	0.30	0.30	0.11	0.11	0.02
	20	0.93	0.84	0.72	0.51	0.32	0.15	0.07	0.05	0.01
	30	0.97	0.94	0.78	0.63	0.32	0.18	0.04	0.01	0.00
	40	0.99	0.94	0.86	0.60	0.30	0.10	0.02	0.00	0.00
	50	1.00	0.99	0.92	0.69	0.30	0.10	0.01	0.00	0.00
	60	1.00	0.99	0.90	0.61	0.27	0.05	0.01	0.00	0.00
	70	1.00	1.00	0.92	0.70	0.28	0.07	0.00	0.00	0.00
	80	1.00	0.99	0.95	0.67	0.23	0.03	0.00	0.00	0.00
	90	1.00	1.00	0.97	0.78	0.23	0.03	0.00	0.00	0.00
	100	1.00	1.00	0.97	0.70	0.20	0.01	0.00	0.00	0.00
	125	1.00	1.00	0.99	0.77	0.23	0.01	0.00	0.00	0.00
	150	1.00	1.00	1.00	0.83	0.15	0.00	0.00	0.00	0.00

(a) Probabilities to yield an accuracy exceeding a certain threshold related to the test sample size.

	n	**mean**	**median**	**min**	**max**	**std**
**test sample size**	10	0.67	0.70	0.20	1.00	0.15
	20	0.67	0.70	0.35	1.00	0.10
	30	0.68	0.67	0.40	0.93	0.08
	40	0.68	0.68	0.42	0.88	0.07
	50	0.68	0.68	0.48	0.86	0.06
	60	0.68	0.67	0.52	0.83	0.05
	70	0.68	0.67	0.54	0.83	0.05
	80	0.67	0.68	0.54	0.82	0.05
	90	0.68	0.68	0.56	0.80	0.04
	100	0.67	0.67	0.56	0.80	0.04
	125	0.68	0.67	0.57	0.80	0.03
	150	0.68	0.67	0.60	0.76	0.03

(b) Summary of the accuracy value distributions for certain sample sizes.

Table F.16: Results as a function of variable test set sizes with a fixed classifier. For **feature selection** an ANOVA was computed inside the the pipeline and the top 10 **features** were taken based on the ANOVA F-values. Following, a **linear SVM** was trained with default parameters. ($C = 1.0$)

test sample size n	≥ accuracy 50	55	60	65	70	75	80	85	90
10	0.73	0.73	0.62	0.49	0.24	0.24	0.09	0.09	0.01
20	0.86	0.73	0.58	0.37	0.19	0.09	0.03	0.03	0.00
30	0.92	0.85	0.63	0.47	0.15	0.08	0.01	0.00	0.00
40	0.96	0.84	0.70	0.37	0.15	0.04	0.01	0.00	0.00
50	0.98	0.94	0.80	0.47	0.15	0.04	0.00	0.00	0.00
60	0.99	0.91	0.72	0.38	0.11	0.02	0.00	0.00	0.00
70	1.00	0.96	0.75	0.43	0.09	0.01	0.00	0.00	0.00
80	1.00	0.97	0.81	0.39	0.08	0.01	0.00	0.00	0.00
90	1.00	0.98	0.81	0.43	0.06	0.00	0.00	0.00	0.00
100	1.00	0.99	0.86	0.36	0.05	0.00	0.00	0.00	0.00
125	1.00	1.00	0.89	0.38	0.03	0.00	0.00	0.00	0.00
150	1.00	1.00	0.93	0.36	0.01	0.00	0.00	0.00	0.00

(a) Probabilities to yield an accuracy exceeding a certain threshold related to the test sample size.

test sample size n	**mean**	**median**	**min**	**max**	**std**
10	0.64	0.60	0.20	1.00	0.15
20	0.64	0.65	0.25	0.95	0.10
30	0.64	0.63	0.40	0.87	0.08
40	0.64	0.65	0.40	0.88	0.07
50	0.65	0.64	0.44	0.82	0.06
60	0.64	0.63	0.47	0.85	0.06
70	0.64	0.64	0.49	0.80	0.05
80	0.64	0.65	0.50	0.81	0.05
90	0.64	0.64	0.51	0.78	0.04
100	0.64	0.64	0.52	0.81	0.04
125	0.64	0.64	0.53	0.75	0.03
150	0.64	0.64	0.55	0.72	0.03

(b) Summary of the accuracy value distributions for certain sample sizes.

Table F.17: Results as a function of variable test set sizes with a fixed classifier. For **feature selection** an ANOVA was computed inside the the pipeline and the top 100 **features** were taken based on the ANOVA F-values. Following, a **Random Forest** was trained with default parameters. ($n_{\text{estimators}} = 100$)

test sample size	n	≥ accuracy 50	55	60	65	70	75	80	85	90
	10	0.73	0.73	0.60	0.47	0.20	0.20	0.05	0.05	0.01
	20	0.86	0.71	0.56	0.34	0.19	0.07	0.02	0.01	0.00
	30	0.92	0.85	0.63	0.44	0.17	0.09	0.02	0.01	0.00
	40	0.96	0.85	0.67	0.35	0.15	0.04	0.01	0.00	0.00
	50	0.97	0.91	0.73	0.40	0.12	0.03	0.00	0.00	0.00
	60	0.99	0.92	0.71	0.34	0.09	0.01	0.00	0.00	0.00
	70	1.00	0.96	0.72	0.39	0.09	0.01	0.00	0.00	0.00
	80	1.00	0.95	0.74	0.32	0.06	0.00	0.00	0.00	0.00
	90	1.00	0.99	0.78	0.37	0.06	0.00	0.00	0.00	0.00
	100	1.00	0.99	0.83	0.32	0.04	0.00	0.00	0.00	0.00
	125	1.00	1.00	0.88	0.30	0.03	0.00	0.00	0.00	0.00
	150	1.00	1.00	0.90	0.33	0.01	0.00	0.00	0.00	0.00

(a) Probabilities to yield an accuracy exceeding a certain threshold related to the test sample size.

test sample size	n	**mean**	**median**	**min**	**max**	**std**
	10	0.63	0.60	0.10	1.00	0.14
	20	0.63	0.65	0.35	0.95	0.10
	30	0.64	0.63	0.37	0.90	0.09
	40	0.64	0.62	0.42	0.88	0.07
	50	0.63	0.64	0.42	0.84	0.06
	60	0.64	0.63	0.48	0.80	0.05
	70	0.64	0.64	0.47	0.79	0.05
	80	0.63	0.64	0.50	0.76	0.05
	90	0.64	0.63	0.51	0.77	0.04
	100	0.64	0.64	0.53	0.75	0.04
	125	0.64	0.64	0.54	0.73	0.03
	150	0.64	0.64	0.57	0.71	0.03

(b) Summary of the accuracy value distributions for certain sample sizes.

Table F.18: Results as a function of variable test set sizes with a fixed classifier. For **feature selection** an ANOVA was computed inside the the pipeline and the top 100 **features** were taken based on the ANOVA F-values. Following, an **SVM** with an **RBF kernel** was trained with default parameters. ($C = 1.0$; $\gamma = {}^{1}/_{n_{\text{feature}}}$)

test sample size n	≥ accuracy 50	55	60	65	70	75	80	85	90
10	0.69	0.69	0.56	0.44	0.19	0.19	0.05	0.05	0.00
20	0.81	0.69	0.54	0.32	0.16	0.06	0.02	0.02	0.00
30	0.89	0.82	0.53	0.34	0.10	0.04	0.00	0.00	0.00
40	0.93	0.80	0.59	0.27	0.10	0.02	0.00	0.00	0.00
50	0.96	0.87	0.61	0.31	0.07	0.01	0.00	0.00	0.00
60	0.97	0.85	0.54	0.19	0.03	0.00	0.00	0.00	0.00
70	0.99	0.92	0.58	0.26	0.04	0.00	0.00	0.00	0.00
80	0.99	0.92	0.67	0.21	0.03	0.00	0.00	0.00	0.00
90	1.00	0.95	0.65	0.22	0.02	0.00	0.00	0.00	0.00
100	1.00	0.96	0.68	0.18	0.02	0.00	0.00	0.00	0.00
125	1.00	0.99	0.74	0.17	0.01	0.00	0.00	0.00	0.00
150	1.00	1.00	0.76	0.12	0.00	0.00	0.00	0.00	0.00

(a) Probabilities to yield an accuracy exceeding a certain threshold related to the test sample size.

test sample size n	**mean**	**median**	**min**	**max**	**std**
10	0.62	0.60	0.10	1.00	0.15
20	0.62	0.60	0.30	0.95	0.10
30	0.62	0.63	0.33	0.87	0.08
40	0.62	0.62	0.38	0.82	0.07
50	0.62	0.62	0.40	0.84	0.06
60	0.61	0.62	0.45	0.80	0.05
70	0.62	0.61	0.46	0.77	0.05
80	0.62	0.62	0.49	0.77	0.05
90	0.62	0.62	0.49	0.77	0.04
100	0.62	0.62	0.47	0.75	0.04
125	0.62	0.62	0.52	0.72	0.03
150	0.62	0.62	0.54	0.71	0.03

(b) Summary of the accuracy value distributions for certain sample sizes.

Table F.19: Results as a function of variable test set sizes with a fixed classifier. For **feature selection** an ANOVA was computed inside the the pipeline and the top 100 **features** were taken based on the ANOVA F-values. Following, a **linear SVM** was trained with default parameters. ($C = 1.0$)

test sample size n	≥ accuracy 50	55	60	65	70	75	80	85	90
10	0.81	0.81	0.71	0.60	0.35	0.35	0.11	0.11	0.02
20	0.94	0.84	0.72	0.52	0.33	0.16	0.06	0.05	0.01
30	0.97	0.95	0.78	0.65	0.33	0.20	0.04	0.01	0.00
40	0.99	0.95	0.86	0.57	0.30	0.09	0.02	0.00	0.00
50	0.99	0.98	0.89	0.67	0.28	0.10	0.01	0.00	0.00
60	1.00	0.99	0.88	0.64	0.26	0.05	0.01	0.00	0.00
70	1.00	0.99	0.92	0.71	0.27	0.06	0.01	0.00	0.00
80	1.00	0.99	0.95	0.65	0.24	0.03	0.00	0.00	0.00
90	1.00	1.00	0.97	0.74	0.24	0.04	0.00	0.00	0.00
100	1.00	1.00	0.98	0.74	0.25	0.03	0.00	0.00	0.00
125	1.00	1.00	0.99	0.81	0.24	0.01	0.00	0.00	0.00
150	1.00	1.00	1.00	0.82	0.15	0.00	0.00	0.00	0.00

(a) Probabilities to yield an accuracy exceeding a certain threshold related to the test sample size.

test sample size n	**mean**	**median**	**min**	**max**	**std**
10	0.68	0.70	0.20	1.00	0.15
20	0.68	0.70	0.35	0.95	0.10
30	0.68	0.67	0.43	0.93	0.08
40	0.67	0.68	0.45	0.85	0.07
50	0.67	0.68	0.48	0.86	0.06
60	0.68	0.68	0.50	0.85	0.05
70	0.68	0.67	0.50	0.84	0.05
80	0.67	0.68	0.50	0.80	0.05
90	0.68	0.68	0.53	0.81	0.04
100	0.68	0.68	0.56	0.79	0.04
125	0.68	0.68	0.58	0.78	0.03
150	0.68	0.68	0.59	0.76	0.03

(b) Summary of the accuracy value distributions for certain sample sizes.

Table F.20: Results as a function of variable test set sizes with a fixed classifier. For **feature selection** an ANOVA was computed inside the the pipeline and the top 1,000 **features** were taken based on the ANOVA F-values. Following, a **Random Forest** was trained with default parameters. ($n_{\text{estimators}} = 100$)

		**≥ accuracy**								
	n	50	55	60	65	70	75	80	85	90
**test sample size**	10	0.82	0.82	0.69	0.62	0.35	0.35	0.13	0.13	0.03
	20	0.93	0.84	0.72	0.53	0.33	0.17	0.06	0.06	0.00
	30	0.98	0.95	0.78	0.64	0.34	0.20	0.06	0.01	0.00
	40	0.99	0.95	0.87	0.56	0.29	0.12	0.03	0.01	0.00
	50	1.00	0.98	0.92	0.71	0.30	0.10	0.01	0.00	0.00
	60	1.00	0.98	0.91	0.66	0.29	0.06	0.01	0.00	0.00
	70	1.00	1.00	0.94	0.73	0.30	0.08	0.01	0.00	0.00
	80	1.00	1.00	0.97	0.71	0.28	0.04	0.00	0.00	0.00
	90	1.00	1.00	0.98	0.81	0.30	0.04	0.00	0.00	0.00
	100	1.00	1.00	0.97	0.73	0.23	0.03	0.00	0.00	0.00
	125	1.00	1.00	0.99	0.81	0.26	0.02	0.00	0.00	0.00
	150	1.00	1.00	1.00	0.88	0.20	0.01	0.00	0.00	0.00

(a) Probabilities to yield an accuracy exceeding a certain threshold related to the test sample size.

	n	**mean**	**median**	**min**	**max**	**std**
**test sample size**	10	0.69	0.70	0.20	1.00	0.15
	20	0.68	0.70	0.40	0.95	0.10
	30	0.68	0.67	0.33	0.93	0.08
	40	0.68	0.68	0.45	0.93	0.07
	50	0.68	0.68	0.44	0.84	0.06
	60	0.68	0.68	0.52	0.83	0.05
	70	0.68	0.69	0.54	0.84	0.05
	80	0.68	0.68	0.55	0.82	0.04
	90	0.68	0.68	0.54	0.80	0.04
	100	0.68	0.68	0.56	0.79	0.04
	125	0.68	0.68	0.58	0.78	0.03
	150	0.68	0.68	0.60	0.77	0.03

(b) Summary of the accuracy value distributions for certain sample sizes.

Table F.21: Results as a function of variable test set sizes with a fixed classifier. For **feature selection** an ANOVA was computed inside the the pipeline and the top 1,000 **features** were taken based on the ANOVA F-values. Following, an **SVM** with an **RBF kernel** was trained with default parameters. ($C = 1.0$; $\gamma = {}^{1}/{n_{\text{feature}}}$)

test sample size n	≥ accuracy 50	55	60	65	70	75	80	85	90
10	0.61	0.61	0.44	0.33	0.13	0.13	0.04	0.04	0.00
20	0.68	0.51	0.35	0.17	0.07	0.03	0.01	0.01	0.00
30	0.77	0.62	0.32	0.20	0.06	0.02	0.00	0.00	0.00
40	0.81	0.61	0.40	0.13	0.03	0.00	0.00	0.00	0.00
50	0.86	0.68	0.41	0.15	0.03	0.00	0.00	0.00	0.00
60	0.90	0.66	0.33	0.09	0.01	0.00	0.00	0.00	0.00
70	0.93	0.72	0.32	0.10	0.01	0.00	0.00	0.00	0.00
80	0.95	0.71	0.33	0.06	0.00	0.00	0.00	0.00	0.00
90	0.96	0.74	0.30	0.05	0.00	0.00	0.00	0.00	0.00
100	0.97	0.73	0.31	0.03	0.00	0.00	0.00	0.00	0.00
125	0.99	0.83	0.27	0.01	0.00	0.00	0.00	0.00	0.00
150	1.00	0.86	0.23	0.01	0.00	0.00	0.00	0.00	0.00

(a) Probabilities to yield an accuracy exceeding a certain threshold related to the test sample size.

test sample size n	**mean**	**median**	**min**	**max**	**std**
10	0.58	0.60	0.10	1.00	0.15
20	0.57	0.60	0.20	0.90	0.11
30	0.58	0.57	0.30	0.80	0.08
40	0.58	0.57	0.38	0.80	0.07
50	0.58	0.58	0.38	0.78	0.07
60	0.58	0.58	0.42	0.75	0.06
70	0.58	0.59	0.43	0.76	0.05
80	0.58	0.57	0.44	0.71	0.05
90	0.58	0.58	0.42	0.77	0.04
100	0.58	0.58	0.46	0.71	0.04
125	0.58	0.58	0.47	0.68	0.03
150	0.58	0.58	0.49	0.68	0.03

(b) Summary of the accuracy value distributions for certain sample sizes.

Table F.22: Results as a function of variable test set sizes with a fixed classifier. For **feature selection** an ANOVA was computed inside the the pipeline and the top 1,000 **features** were taken based on the ANOVA F-values. Following, a **linear SVM** was trained with default parameters. ($C = 1.0$)

test sample size n	≥ accuracy 50	55	60	65	70	75	80	85	90
10	0.84	0.84	0.73	0.61	0.34	0.34	0.13	0.13	0.02
20	0.94	0.86	0.75	0.56	0.39	0.19	0.08	0.07	0.01
30	0.98	0.95	0.81	0.69	0.37	0.22	0.05	0.02	0.00
40	1.00	0.97	0.91	0.65	0.37	0.12	0.03	0.00	0.00
50	1.00	0.99	0.93	0.74	0.35	0.12	0.01	0.00	0.00
60	1.00	0.99	0.92	0.72	0.34	0.09	0.01	0.00	0.00
70	1.00	1.00	0.94	0.76	0.32	0.09	0.01	0.00	0.00
80	1.00	1.00	0.97	0.77	0.33	0.07	0.00	0.00	0.00
90	1.00	1.00	0.98	0.81	0.32	0.06	0.00	0.00	0.00
100	1.00	1.00	0.99	0.80	0.31	0.04	0.00	0.00	0.00
125	1.00	1.00	1.00	0.87	0.36	0.03	0.00	0.00	0.00
150	1.00	1.00	1.00	0.92	0.27	0.01	0.00	0.00	0.00

(a) Probabilities to yield an accuracy exceeding a certain threshold related to the test sample size.

test sample size n	**mean**	**median**	**min**	**max**	**std**
10	0.69	0.70	0.20	1.00	0.15
20	0.69	0.70	0.30	0.95	0.10
30	0.69	0.70	0.43	0.93	0.08
40	0.69	0.68	0.47	0.88	0.07
50	0.69	0.68	0.50	0.88	0.06
60	0.69	0.68	0.45	0.85	0.05
70	0.68	0.69	0.53	0.83	0.05
80	0.69	0.69	0.53	0.81	0.04
90	0.69	0.69	0.52	0.81	0.04
100	0.69	0.69	0.56	0.82	0.04
125	0.69	0.69	0.60	0.78	0.03
150	0.69	0.69	0.61	0.77	0.03

(b) Summary of the accuracy value distributions for certain sample sizes.

Table F.23: Results as a function of variable test set sizes with a fixed classifier. For **feature selection** an ANOVA was computed inside the the pipeline and the top 10,000 **features** were taken based on the ANOVA F-values. Following, a **Random Forest** was trained with default parameters. ($n_{\text{estimators}} = 100$)

test sample size	$\geq$ **accuracy**								
n	50	55	60	65	70	75	80	85	90
10	0.82	0.82	0.71	0.61	0.35	0.35	0.13	0.13	0.03
20	0.94	0.86	0.77	0.58	0.38	0.21	0.10	0.08	0.01
30	0.98	0.96	0.83	0.71	0.40	0.24	0.05	0.02	0.00
40	1.00	0.97	0.90	0.67	0.39	0.15	0.04	0.01	0.00
50	1.00	0.99	0.94	0.77	0.36	0.13	0.01	0.00	0.00
60	1.00	0.99	0.94	0.76	0.40	0.10	0.01	0.00	0.00
70	1.00	1.00	0.96	0.79	0.34	0.09	0.01	0.00	0.00
80	1.00	1.00	0.97	0.79	0.34	0.07	0.00	0.00	0.00
90	1.00	1.00	0.98	0.86	0.33	0.07	0.00	0.00	0.00
100	1.00	1.00	0.99	0.82	0.36	0.04	0.00	0.00	0.00
125	1.00	1.00	1.00	0.89	0.40	0.03	0.00	0.00	0.00
150	1.00	1.00	1.00	0.92	0.30	0.01	0.00	0.00	0.00

(a) Probabilities to yield an accuracy exceeding a certain threshold related to the test sample size.

test sample size n	**mean**	**median**	**min**	**max**	**std**
10	0.69	0.70	0.20	1.00	0.15
20	0.69	0.70	0.35	0.95	0.10
30	0.69	0.70	0.40	0.90	0.08
40	0.69	0.70	0.47	0.90	0.07
50	0.69	0.70	0.48	0.86	0.06
60	0.69	0.70	0.52	0.85	0.05
70	0.69	0.69	0.53	0.84	0.05
80	0.69	0.69	0.54	0.84	0.05
90	0.69	0.69	0.58	0.81	0.04
100	0.69	0.69	0.57	0.82	0.04
125	0.69	0.70	0.58	0.78	0.03
150	0.69	0.69	0.61	0.79	0.03

(b) Summary of the accuracy value distributions for certain sample sizes.

Table F.24: Results as a function of variable test set sizes with a fixed classifier. For **feature selection** an ANOVA was computed inside the the pipeline and the top 10,000 **features** were taken based on the ANOVA F-values. Following, an **SVM** with an **RBF kernel** was trained with default parameters. ($C = 1.0$; $\gamma = {}^1/n_{\text{feature}}$)

test sample size	≥ accuracy								
n	50	55	60	65	70	75	80	85	90
10	0.77	0.77	0.66	0.55	0.29	0.29	0.09	0.09	0.02
20	0.89	0.79	0.66	0.43	0.24	0.12	0.04	0.02	0.00
30	0.95	0.89	0.70	0.54	0.24	0.13	0.02	0.01	0.00
40	0.98	0.92	0.78	0.45	0.19	0.05	0.01	0.00	0.00
50	0.99	0.95	0.81	0.54	0.19	0.06	0.00	0.00	0.00
60	0.99	0.95	0.81	0.48	0.17	0.02	0.00	0.00	0.00
70	1.00	0.98	0.82	0.51	0.16	0.03	0.00	0.00	0.00
80	1.00	0.98	0.88	0.47	0.13	0.01	0.00	0.00	0.00
90	1.00	0.99	0.88	0.56	0.12	0.01	0.00	0.00	0.00
100	1.00	1.00	0.92	0.49	0.09	0.00	0.00	0.00	0.00
125	1.00	1.00	0.95	0.53	0.08	0.00	0.00	0.00	0.00
150	1.00	1.00	0.98	0.54	0.04	0.00	0.00	0.00	0.00

(a) Probabilities to yield an accuracy exceeding a certain threshold related to the test sample size.

test sample size n	**mean**	**median**	**min**	**max**	**std**
10	0.66	0.70	0.20	1.00	0.15
20	0.66	0.65	0.30	0.95	0.10
30	0.66	0.67	0.43	0.90	0.08
40	0.65	0.65	0.42	0.85	0.07
50	0.66	0.66	0.46	0.82	0.06
60	0.65	0.65	0.47	0.82	0.05
70	0.65	0.66	0.47	0.81	0.05
80	0.65	0.65	0.46	0.81	0.05
90	0.66	0.66	0.53	0.81	0.04
100	0.65	0.65	0.53	0.77	0.04
125	0.65	0.66	0.55	0.75	0.03
150	0.65	0.65	0.56	0.75	0.03

(b) Summary of the accuracy value distributions for certain sample sizes.

Table F.25: Results as a function of variable test set sizes with a fixed classifier. For **feature selection** an ANOVA was computed inside the the pipeline and the top 10,000 **features** were taken based on the ANOVA F-values. Following, a **linear SVM** was trained with default parameters. ($C = 1.0$)

test sample size n	≥ accuracy 50	55	60	65	70	75	80	85	90
10	0.86	0.86	0.75	0.65	0.38	0.38	0.16	0.16	0.03
20	0.96	0.89	0.80	0.61	0.42	0.24	0.11	0.10	0.01
30	0.98	0.96	0.83	0.71	0.39	0.26	0.06	0.02	0.00
40	0.99	0.97	0.91	0.69	0.41	0.17	0.04	0.02	0.00
50	1.00	0.99	0.95	0.80	0.43	0.20	0.03	0.01	0.00
60	1.00	0.99	0.95	0.77	0.44	0.14	0.02	0.00	0.00
70	1.00	1.00	0.96	0.83	0.44	0.14	0.01	0.00	0.00
80	1.00	1.00	0.99	0.84	0.44	0.07	0.00	0.00	0.00
90	1.00	1.00	0.99	0.90	0.46	0.11	0.01	0.00	0.00
100	1.00	1.00	0.99	0.89	0.45	0.08	0.00	0.00	0.00
125	1.00	1.00	1.00	0.94	0.49	0.06	0.00	0.00	0.00
150	1.00	1.00	1.00	0.97	0.45	0.04	0.00	0.00	0.00

(a) Probabilities to yield an accuracy exceeding a certain threshold related to the test sample size.

test sample size n	**mean**	**median**	**min**	**max**	**std**
10	0.70	0.70	0.30	1.00	0.14
20	0.70	0.70	0.35	0.95	0.10
30	0.69	0.70	0.43	0.90	0.08
40	0.70	0.70	0.47	0.93	0.07
50	0.70	0.70	0.50	0.90	0.06
60	0.70	0.70	0.48	0.87	0.05
70	0.70	0.70	0.56	0.86	0.05
80	0.70	0.70	0.54	0.81	0.04
90	0.70	0.70	0.58	0.83	0.04
100	0.70	0.70	0.57	0.82	0.04
125	0.70	0.70	0.59	0.79	0.03
150	0.70	0.70	0.62	0.78	0.03

(b) Summary of the accuracy value distributions for certain sample sizes.

Table F.26: Results as a function of variable test set sizes with a fixed classifier. For **feature selection** an ANOVA was computed inside the the pipeline and the top 50,000 **features** were taken based on the ANOVA F-values. Following, a **Random Forest** was trained with default parameters. ($n_{\text{estimators}} = 100$)

test sample size n	≥ accuracy 50	55	60	65	70	75	80	85	90
10	0.77	0.77	0.64	0.51	0.26	0.26	0.09	0.09	0.01
20	0.89	0.77	0.64	0.40	0.23	0.10	0.03	0.02	0.00
30	0.96	0.90	0.71	0.54	0.23	0.12	0.02	0.01	0.00
40	0.98	0.91	0.76	0.47	0.21	0.06	0.01	0.00	0.00
50	0.99	0.96	0.82	0.50	0.15	0.03	0.00	0.00	0.00
60	1.00	0.95	0.79	0.48	0.16	0.03	0.00	0.00	0.00
70	1.00	0.98	0.81	0.52	0.14	0.02	0.00	0.00	0.00
80	1.00	0.98	0.86	0.48	0.11	0.01	0.00	0.00	0.00
90	1.00	0.99	0.88	0.52	0.12	0.01	0.00	0.00	0.00
100	1.00	1.00	0.90	0.48	0.10	0.01	0.00	0.00	0.00
125	1.00	1.00	0.95	0.52	0.07	0.00	0.00	0.00	0.00
150	1.00	1.00	0.97	0.56	0.04	0.00	0.00	0.00	0.00

(a) Probabilities to yield an accuracy exceeding a certain threshold related to the test sample size.

test sample size n	**mean**	**median**	**min**	**max**	**std**
10	0.65	0.70	0.20	1.00	0.15
20	0.65	0.65	0.30	0.90	0.10
30	0.66	0.67	0.33	0.93	0.08
40	0.66	0.65	0.42	0.88	0.07
50	0.65	0.64	0.48	0.82	0.06
60	0.65	0.65	0.47	0.82	0.06
70	0.65	0.66	0.41	0.80	0.05
80	0.65	0.65	0.51	0.80	0.05
90	0.65	0.66	0.53	0.80	0.04
100	0.65	0.65	0.54	0.77	0.04
125	0.65	0.66	0.57	0.75	0.03
150	0.65	0.65	0.57	0.73	0.03

(b) Summary of the accuracy value distributions for certain sample sizes.

Table F.27: Results as a function of variable test set sizes with a fixed classifier. For **feature selection** an ANOVA was computed inside the the pipeline and the top 50,000 **features** were taken based on the ANOVA F-values. Following, an **SVM** with an **RBF kernel** was trained with default parameters. ($C = 1.0$; $\gamma = {}^{1}/{n_{\text{feature}}}$)

test sample size	n	≥ accuracy 50	55	60	65	70	75	80	85	90
	10	0.79	0.79	0.64	0.53	0.26	0.26	0.08	0.08	0.01
	20	0.90	0.79	0.66	0.44	0.28	0.14	0.05	0.05	0.00
	30	0.95	0.90	0.70	0.52	0.23	0.12	0.03	0.01	0.00
	40	0.98	0.92	0.79	0.47	0.20	0.06	0.01	0.00	0.00
	50	0.99	0.95	0.84	0.56	0.19	0.06	0.00	0.00	0.00
	60	0.99	0.96	0.81	0.48	0.16	0.03	0.00	0.00	0.00
	70	1.00	0.99	0.83	0.55	0.15	0.02	0.00	0.00	0.00
	80	1.00	0.98	0.89	0.52	0.13	0.01	0.00	0.00	0.00
	90	1.00	1.00	0.90	0.56	0.11	0.01	0.00	0.00	0.00
	100	1.00	0.99	0.92	0.50	0.10	0.00	0.00	0.00	0.00
	125	1.00	1.00	0.95	0.54	0.10	0.00	0.00	0.00	0.00
	150	1.00	1.00	0.99	0.61	0.04	0.00	0.00	0.00	0.00

(a) Probabilities to yield an accuracy exceeding a certain threshold related to the test sample size.

test sample size	n	**mean**	**median**	**min**	**max**	**std**
	10	0.66	0.70	0.30	1.00	0.14
	20	0.66	0.65	0.30	0.95	0.11
	30	0.65	0.67	0.40	0.90	0.08
	40	0.66	0.65	0.42	0.90	0.07
	50	0.66	0.66	0.48	0.84	0.06
	60	0.65	0.65	0.40	0.83	0.06
	70	0.66	0.66	0.47	0.80	0.05
	80	0.66	0.66	0.51	0.80	0.05
	90	0.66	0.66	0.54	0.77	0.04
	100	0.65	0.65	0.51	0.78	0.04
	125	0.66	0.66	0.55	0.77	0.03
	150	0.66	0.66	0.57	0.77	0.03

(b) Summary of the accuracy value distributions for certain sample sizes.

Table F.28: Results as a function of variable test set sizes with a fixed classifier. For **feature selection** an ANOVA was computed inside the the pipeline and the top 50,000 **features** were taken based on the ANOVA F-values. Following, a **linear SVM** was trained with default parameters. ($C = 1.0$)

test sample size n	≥ accuracy 50	55	60	65	70	75	80	85	90
10	0.78	0.78	0.64	0.56	0.32	0.32	0.12	0.12	0.02
20	0.93	0.83	0.70	0.49	0.32	0.16	0.07	0.06	0.00
30	0.96	0.91	0.75	0.63	0.30	0.16	0.03	0.01	0.00
40	0.99	0.93	0.83	0.55	0.29	0.09	0.02	0.00	0.00
50	0.99	0.97	0.87	0.63	0.24	0.09	0.01	0.00	0.00
60	1.00	0.98	0.87	0.58	0.23	0.05	0.00	0.00	0.00
70	1.00	0.99	0.90	0.67	0.23	0.05	0.00	0.00	0.00
80	1.00	1.00	0.94	0.64	0.24	0.02	0.00	0.00	0.00
90	1.00	0.99	0.93	0.70	0.19	0.03	0.00	0.00	0.00
100	1.00	1.00	0.96	0.67	0.18	0.01	0.00	0.00	0.00
125	1.00	1.00	0.98	0.71	0.20	0.01	0.00	0.00	0.00
150	1.00	1.00	0.99	0.77	0.12	0.00	0.00	0.00	0.00

(a) Probabilities to yield an accuracy exceeding a certain threshold related to the test sample size.

test sample size n	**mean**	**median**	**min**	**max**	**std**
10	0.67	0.70	0.10	1.00	0.15
20	0.67	0.65	0.30	0.95	0.10
30	0.67	0.67	0.30	0.93	0.08
40	0.67	0.68	0.45	0.88	0.07
50	0.67	0.67	0.44	0.84	0.06
60	0.67	0.67	0.48	0.83	0.05
70	0.67	0.67	0.49	0.81	0.05
80	0.67	0.68	0.55	0.80	0.04
90	0.67	0.67	0.52	0.81	0.04
100	0.67	0.67	0.56	0.79	0.04
125	0.67	0.67	0.54	0.76	0.03
150	0.67	0.67	0.58	0.75	0.03

(b) Summary of the accuracy value distributions for certain sample sizes.

Table F.29: Results as a function of variable test set sizes with a fixed classifier. To reduce the dimensionality of the feature space a **PCA** was performed and 10 **components** were retained. Following, a **Random Forest** was trained with default parameters. ($n_{\text{estimators}} = 100$)

		≥ **accuracy**								
	n	50	55	60	65	70	75	80	85	90
**test sample size**	10	0.03	0.03	0.00	0.00	0.00	0.00	0.00	0.00	0.00
	20	0.07	0.00	0.00	0.00	0.00	0.00	0.00	0.00	0.00
	30	0.10	0.00	0.00	0.00	0.00	0.00	0.00	0.00	0.00
	40	0.13	0.00	0.00	0.00	0.00	0.00	0.00	0.00	0.00
	50	0.16	0.00	0.00	0.00	0.00	0.00	0.00	0.00	0.00
	60	0.20	0.00	0.00	0.00	0.00	0.00	0.00	0.00	0.00
	70	0.23	0.00	0.00	0.00	0.00	0.00	0.00	0.00	0.00
	80	0.27	0.00	0.00	0.00	0.00	0.00	0.00	0.00	0.00
	90	0.28	0.00	0.00	0.00	0.00	0.00	0.00	0.00	0.00
	100	0.35	0.00	0.00	0.00	0.00	0.00	0.00	0.00	0.00
	125	0.41	0.00	0.00	0.00	0.00	0.00	0.00	0.00	0.00
	150	0.51	0.00	0.00	0.00	0.00	0.00	0.00	0.00	0.00

(a) Probabilities to yield an accuracy exceeding a certain threshold related to the test sample size.

	n	**mean**	**median**	**min**	**max**	**std**
**test sample size**	10	0.50	0.50	0.50	0.60	0.02
	20	0.50	0.50	0.50	0.55	0.01
	30	0.50	0.50	0.50	0.53	0.01
	40	0.50	0.50	0.50	0.53	0.01
	50	0.50	0.50	0.50	0.52	0.01
	60	0.50	0.50	0.50	0.52	0.01
	70	0.50	0.50	0.50	0.51	0.01
	80	0.50	0.50	0.50	0.51	0.01
	90	0.50	0.50	0.50	0.51	0.01
	100	0.50	0.50	0.50	0.51	0.00
	125	0.50	0.50	0.50	0.51	0.00
	150	0.50	0.51	0.50	0.51	0.00

(b) Summary of the accuracy value distributions for certain sample sizes.

Table F.30: Results as a function of variable test set sizes with a fixed classifier. To reduce the dimensionality of the feature space a **PCA** was performed and 10 **components** were retained. Following, an **SVM** with an **RBF kernel** was trained with default parameters. ($C = 1.0$; $\gamma = {}^{1}/{n_{\text{feature}}}$)

test sample size	n	≥ accuracy 50	55	60	65	70	75	80	85	90
	10	0.58	0.58	0.45	0.32	0.13	0.13	0.04	0.04	0.00
	20	0.70	0.52	0.37	0.17	0.07	0.02	0.00	0.00	0.00
	30	0.74	0.60	0.31	0.18	0.04	0.02	0.00	0.00	0.00
	40	0.81	0.57	0.36	0.11	0.03	0.00	0.00	0.00	0.00
	50	0.88	0.67	0.39	0.12	0.02	0.00	0.00	0.00	0.00
	60	0.88	0.61	0.25	0.07	0.01	0.00	0.00	0.00	0.00
	70	0.92	0.67	0.30	0.07	0.00	0.00	0.00	0.00	0.00
	80	0.92	0.65	0.28	0.03	0.00	0.00	0.00	0.00	0.00
	90	0.92	0.69	0.22	0.04	0.00	0.00	0.00	0.00	0.00
	100	0.95	0.66	0.25	0.02	0.00	0.00	0.00	0.00	0.00
	125	0.99	0.78	0.21	0.01	0.00	0.00	0.00	0.00	0.00
	150	0.99	0.81	0.16	0.00	0.00	0.00	0.00	0.00	0.00

(a) Probabilities to yield an accuracy exceeding a certain threshold related to the test sample size.

test sample size	n	**mean**	**median**	**min**	**max**	**std**
	10	0.58	0.60	0.10	1.00	0.16
	20	0.58	0.60	0.25	0.85	0.10
	30	0.57	0.57	0.30	0.83	0.08
	40	0.57	0.57	0.35	0.78	0.07
	50	0.58	0.58	0.36	0.78	0.06
	60	0.57	0.57	0.42	0.75	0.06
	70	0.58	0.57	0.43	0.73	0.05
	80	0.57	0.57	0.38	0.70	0.05
	90	0.57	0.57	0.41	0.71	0.05
	100	0.57	0.57	0.43	0.70	0.04
	125	0.57	0.58	0.46	0.68	0.03
	150	0.57	0.57	0.49	0.67	0.03

(b) Summary of the accuracy value distributions for certain sample sizes.

Table F.31: Results as a function of variable test set sizes with a fixed classifier. To reduce the dimensionality of the feature space a **PCA** was performed and 10 **components** were retained. Following, a **linear SVM** was trained with default parameters. ($C = 1.0$)

test sample size	n	≥ accuracy 50	55	60	65	70	75	80	85	90
	10	0.79	0.79	0.67	0.56	0.31	0.31	0.10	0.10	0.02
	20	0.92	0.84	0.72	0.51	0.30	0.17	0.07	0.06	0.01
	30	0.97	0.92	0.74	0.60	0.30	0.18	0.03	0.01	0.00
	40	0.99	0.94	0.82	0.57	0.28	0.09	0.02	0.00	0.00
	50	0.99	0.98	0.88	0.60	0.25	0.08	0.00	0.00	0.00
	60	0.99	0.98	0.88	0.60	0.25	0.04	0.01	0.00	0.00
	70	1.00	1.00	0.90	0.66	0.23	0.06	0.00	0.00	0.00
	80	1.00	0.99	0.92	0.61	0.21	0.02	0.00	0.00	0.00
	90	1.00	1.00	0.94	0.67	0.20	0.02	0.00	0.00	0.00
	100	1.00	1.00	0.97	0.68	0.18	0.01	0.00	0.00	0.00
	125	1.00	1.00	0.98	0.69	0.17	0.01	0.00	0.00	0.00
	150	1.00	1.00	0.99	0.75	0.10	0.00	0.00	0.00	0.00

(a) Probabilities to yield an accuracy exceeding a certain threshold related to the test sample size.

test sample size	n	**mean**	**median**	**min**	**max**	**std**
	10	0.67	0.70	0.10	1.00	0.15
	20	0.67	0.70	0.30	0.95	0.11
	30	0.67	0.67	0.37	0.90	0.08
	40	0.67	0.68	0.42	0.90	0.07
	50	0.67	0.66	0.40	0.82	0.06
	60	0.67	0.67	0.47	0.83	0.05
	70	0.67	0.67	0.53	0.81	0.05
	80	0.67	0.68	0.51	0.81	0.05
	90	0.67	0.67	0.54	0.80	0.04
	100	0.67	0.67	0.55	0.78	0.04
	125	0.67	0.67	0.58	0.77	0.03
	150	0.67	0.67	0.59	0.77	0.03

(b) Summary of the accuracy value distributions for certain sample sizes.

Table F.32: Results as a function of variable test set sizes with a fixed classifier. To reduce the dimensionality of the feature space a **PCA** was performed and 50 **components** were retained. Following, a **Random Forest** was trained with default parameters. ($n_{\text{estimators}} = 100$)

		≥ **accuracy**								
	n	50	55	60	65	70	75	80	85	90
**test sample size**	10	0.15	0.15	0.01	0.01	0.00	0.00	0.00	0.00	0.00
	20	0.27	0.04	0.01	0.00	0.00	0.00	0.00	0.00	0.00
	30	0.38	0.07	0.00	0.00	0.00	0.00	0.00	0.00	0.00
	40	0.42	0.02	0.00	0.00	0.00	0.00	0.00	0.00	0.00
	50	0.44	0.03	0.00	0.00	0.00	0.00	0.00	0.00	0.00
	60	0.51	0.00	0.00	0.00	0.00	0.00	0.00	0.00	0.00
	70	0.53	0.00	0.00	0.00	0.00	0.00	0.00	0.00	0.00
	80	0.59	0.00	0.00	0.00	0.00	0.00	0.00	0.00	0.00
	90	0.61	0.00	0.00	0.00	0.00	0.00	0.00	0.00	0.00
	100	0.68	0.00	0.00	0.00	0.00	0.00	0.00	0.00	0.00
	125	0.79	0.00	0.00	0.00	0.00	0.00	0.00	0.00	0.00
	150	0.79	0.00	0.00	0.00	0.00	0.00	0.00	0.00	0.00

(a) Probabilities to yield an accuracy exceeding a certain threshold related to the test sample size.

	n	**mean**	**median**	**min**	**max**	**std**
**test sample size**	10	0.51	0.50	0.40	0.70	0.05
	20	0.51	0.50	0.45	0.65	0.03
	30	0.51	0.50	0.43	0.63	0.03
	40	0.51	0.50	0.45	0.60	0.02
	50	0.51	0.50	0.46	0.58	0.02
	60	0.51	0.52	0.47	0.57	0.02
	70	0.51	0.51	0.47	0.56	0.02
	80	0.51	0.51	0.47	0.55	0.01
	90	0.51	0.51	0.48	0.54	0.01
	100	0.51	0.51	0.48	0.55	0.01
	125	0.51	0.51	0.48	0.54	0.01
	150	0.51	0.51	0.49	0.53	0.01

(b) Summary of the accuracy value distributions for certain sample sizes.

Table F.33: Results as a function of variable test set sizes with a fixed classifier. To reduce the dimensionality of the feature space a **PCA** was performed and 50 **components** were retained. Following, an **SVM** with an **RBF kernel** was trained with default parameters. ($C = 1.0$; $\gamma = {}^{1}/{n_{\text{feature}}}$)

test sample size	n	≥ accuracy 50	55	60	65	70	75	80	85	90
	10	0.67	0.67	0.55	0.43	0.18	0.18	0.06	0.06	0.01
	20	0.85	0.70	0.56	0.34	0.19	0.08	0.02	0.02	0.00
	30	0.90	0.82	0.58	0.41	0.15	0.07	0.01	0.00	0.00
	40	0.96	0.82	0.63	0.31	0.10	0.03	0.00	0.00	0.00
	50	0.95	0.88	0.68	0.35	0.10	0.02	0.00	0.00	0.00
	60	0.99	0.88	0.61	0.27	0.06	0.01	0.00	0.00	0.00
	70	0.98	0.93	0.65	0.32	0.04	0.01	0.00	0.00	0.00
	80	0.99	0.94	0.71	0.27	0.04	0.00	0.00	0.00	0.00
	90	1.00	0.96	0.71	0.28	0.04	0.00	0.00	0.00	0.00
	100	1.00	0.96	0.75	0.24	0.02	0.00	0.00	0.00	0.00
	125	1.00	0.99	0.78	0.22	0.01	0.00	0.00	0.00	0.00
	150	1.00	1.00	0.82	0.20	0.00	0.00	0.00	0.00	0.00

(a) Probabilities to yield an accuracy exceeding a certain threshold related to the test sample size.

test sample size	n	**mean**	**median**	**min**	**max**	**std**
	10	0.62	0.60	0.20	1.00	0.15
	20	0.63	0.65	0.30	0.90	0.10
	30	0.63	0.63	0.37	0.83	0.09
	40	0.63	0.62	0.40	0.88	0.07
	50	0.63	0.62	0.42	0.86	0.06
	60	0.62	0.62	0.45	0.80	0.05
	70	0.63	0.63	0.44	0.77	0.05
	80	0.63	0.62	0.45	0.75	0.05
	90	0.63	0.62	0.50	0.76	0.04
	100	0.63	0.63	0.47	0.77	0.04
	125	0.63	0.62	0.54	0.74	0.03
	150	0.63	0.63	0.55	0.70	0.03

(b) Summary of the accuracy value distributions for certain sample sizes.

Table F.34: Results as a function of variable test set sizes with a fixed classifier. To reduce the dimensionality of the feature space a **PCA** was performed and 50 **components** were retained. Following, a **linear SVM** was trained with default parameters. ($C = 1.0$)

		≥ **accuracy**								
	n	50	55	60	65	70	75	80	85	90
**test sample size**	10	0.72	0.72	0.58	0.45	0.22	0.22	0.08	0.08	0.01
	20	0.88	0.75	0.60	0.38	0.21	0.08	0.02	0.02	0.00
	30	0.94	0.88	0.64	0.46	0.16	0.09	0.01	0.00	0.00
	40	0.97	0.87	0.70	0.37	0.15	0.04	0.01	0.00	0.00
	50	0.98	0.94	0.78	0.47	0.13	0.04	0.00	0.00	0.00
	60	0.99	0.93	0.73	0.39	0.12	0.03	0.00	0.00	0.00
	70	0.99	0.97	0.78	0.47	0.11	0.02	0.00	0.00	0.00
	80	1.00	0.98	0.82	0.39	0.09	0.01	0.00	0.00	0.00
	90	1.00	0.99	0.84	0.43	0.08	0.01	0.00	0.00	0.00
	100	1.00	0.98	0.88	0.39	0.05	0.00	0.00	0.00	0.00
	125	1.00	1.00	0.89	0.37	0.04	0.00	0.00	0.00	0.00
	150	1.00	1.00	0.94	0.41	0.02	0.00	0.00	0.00	0.00

(a) Probabilities to yield an accuracy exceeding a certain threshold related to the test sample size.

	n	**mean**	**median**	**min**	**max**	**std**
**test sample size**	10	0.64	0.60	0.20	1.00	0.15
	20	0.64	0.65	0.25	0.95	0.10
	30	0.64	0.63	0.40	0.90	0.08
	40	0.64	0.65	0.40	0.85	0.07
	50	0.65	0.64	0.44	0.84	0.06
	60	0.64	0.65	0.43	0.80	0.06
	70	0.65	0.64	0.47	0.80	0.05
	80	0.64	0.65	0.49	0.78	0.05
	90	0.64	0.64	0.50	0.78	0.04
	100	0.64	0.64	0.52	0.76	0.04
	125	0.64	0.64	0.54	0.76	0.03
	150	0.64	0.65	0.55	0.72	0.03

(b) Summary of the accuracy value distributions for certain sample sizes.

Table F.35: Results as a function of variable test set sizes with a fixed classifier. To reduce the dimensionality of the feature space a **PCA** was performed and 50 **components** were retained. For **feature selection** an ANOVA was computed inside the the pipeline and the top 10 **features** were taken based on the ANOVA F-values. Following, a **Random Forest** was trained with default parameters. ($n_{\text{estimators}} = 100$)

test sample size n	≥ accuracy 50	55	60	65	70	75	80	85	90
10	0.03	0.03	0.00	0.00	0.00	0.00	0.00	0.00	0.00
20	0.08	0.00	0.00	0.00	0.00	0.00	0.00	0.00	0.00
30	0.07	0.00	0.00	0.00	0.00	0.00	0.00	0.00	0.00
40	0.10	0.00	0.00	0.00	0.00	0.00	0.00	0.00	0.00
50	0.11	0.00	0.00	0.00	0.00	0.00	0.00	0.00	0.00
60	0.12	0.00	0.00	0.00	0.00	0.00	0.00	0.00	0.00
70	0.13	0.00	0.00	0.00	0.00	0.00	0.00	0.00	0.00
80	0.15	0.00	0.00	0.00	0.00	0.00	0.00	0.00	0.00
90	0.14	0.00	0.00	0.00	0.00	0.00	0.00	0.00	0.00
100	0.14	0.00	0.00	0.00	0.00	0.00	0.00	0.00	0.00
125	0.25	0.00	0.00	0.00	0.00	0.00	0.00	0.00	0.00
150	0.12	0.00	0.00	0.00	0.00	0.00	0.00	0.00	0.00

(a) Probabilities to yield an accuracy exceeding a certain threshold related to the test sample size.

test sample size n	**mean**	**median**	**min**	**max**	**std**
10	0.50	0.50	0.40	0.60	0.03
20	0.50	0.50	0.40	0.55	0.02
30	0.50	0.50	0.43	0.53	0.02
40	0.50	0.50	0.45	0.53	0.01
50	0.50	0.50	0.46	0.52	0.01
60	0.50	0.50	0.47	0.52	0.01
70	0.50	0.50	0.47	0.51	0.01
80	0.50	0.50	0.47	0.51	0.01
90	0.50	0.50	0.48	0.51	0.01
100	0.50	0.50	0.48	0.51	0.01
125	0.50	0.50	0.48	0.51	0.01
150	0.50	0.50	0.49	0.51	0.01

(b) Summary of the accuracy value distributions for certain sample sizes.

Table F.36: Results as a function of variable test set sizes with a fixed classifier. To reduce the dimensionality of the feature space a **PCA** was performed and 50 **components** were retained. For **feature selection** an ANOVA was computed inside the the pipeline and the top 10 **features** were taken based on the ANOVA F-values. Following, an **SVM** with an **RBF kernel** was trained with default parameters. ($C = 1.0$; $\gamma = 1/n_{\text{feature}}$)

test sample size: n	≥ accuracy: 50	55	60	65	70	75	80	85	90
10	0.46	0.46	0.34	0.23	0.06	0.06	0.02	0.02	0.00
20	0.54	0.35	0.23	0.09	0.04	0.01	0.00	0.00	0.00
30	0.59	0.42	0.19	0.09	0.01	0.01	0.00	0.00	0.00
40	0.62	0.32	0.17	0.03	0.01	0.00	0.00	0.00	0.00
50	0.68	0.44	0.16	0.03	0.00	0.00	0.00	0.00	0.00
60	0.68	0.36	0.11	0.02	0.00	0.00	0.00	0.00	0.00
70	0.74	0.42	0.09	0.01	0.00	0.00	0.00	0.00	0.00
80	0.75	0.34	0.09	0.01	0.00	0.00	0.00	0.00	0.00
90	0.76	0.39	0.06	0.01	0.00	0.00	0.00	0.00	0.00
100	0.80	0.36	0.07	0.00	0.00	0.00	0.00	0.00	0.00
125	0.88	0.38	0.04	0.00	0.00	0.00	0.00	0.00	0.00
150	0.87	0.32	0.01	0.00	0.00	0.00	0.00	0.00	0.00

(a) Probabilities to yield an accuracy exceeding a certain threshold related to the test sample size.

test sample size: n	**mean**	**median**	**min**	**max**	**std**
10	0.53	0.50	0.10	1.00	0.16
20	0.54	0.55	0.15	0.85	0.11
30	0.54	0.53	0.27	0.80	0.09
40	0.53	0.53	0.33	0.75	0.07
50	0.54	0.54	0.34	0.72	0.06
60	0.54	0.53	0.35	0.75	0.06
70	0.54	0.54	0.39	0.69	0.05
80	0.54	0.54	0.38	0.70	0.05
90	0.54	0.53	0.42	0.69	0.04
100	0.54	0.54	0.36	0.67	0.04
125	0.54	0.54	0.43	0.66	0.03
150	0.54	0.53	0.44	0.65	0.03

(b) Summary of the accuracy value distributions for certain sample sizes.

Table F.37: Results as a function of variable test set sizes with a fixed classifier. To reduce the dimensionality of the feature space a **PCA** was performed and 50 **components** were retained. For **feature selection** an ANOVA was computed inside the the pipeline and the top 10 **features** were taken based on the ANOVA F-values. Following, a **linear SVM** was trained with default parameters. ($C = 1.0$)

test sample size n	≥ accuracy 50	55	60	65	70	75	80	85	90
10	0.44	0.44	0.19	0.14	0.03	0.03	0.01	0.01	0.00
20	0.60	0.33	0.14	0.03	0.00	0.00	0.00	0.00	0.00
30	0.67	0.42	0.09	0.02	0.00	0.00	0.00	0.00	0.00
40	0.75	0.36	0.09	0.01	0.00	0.00	0.00	0.00	0.00
50	0.77	0.43	0.08	0.01	0.00	0.00	0.00	0.00	0.00
60	0.80	0.32	0.04	0.00	0.00	0.00	0.00	0.00	0.00
70	0.84	0.43	0.05	0.00	0.00	0.00	0.00	0.00	0.00
80	0.89	0.33	0.04	0.00	0.00	0.00	0.00	0.00	0.00
90	0.91	0.41	0.02	0.00	0.00	0.00	0.00	0.00	0.00
100	0.92	0.34	0.02	0.00	0.00	0.00	0.00	0.00	0.00
125	0.97	0.36	0.01	0.00	0.00	0.00	0.00	0.00	0.00
150	0.97	0.36	0.00	0.00	0.00	0.00	0.00	0.00	0.00

(a) Probabilities to yield an accuracy exceeding a certain threshold related to the test sample size.

test sample size n	**mean**	**median**	**min**	**max**	**std**
10	0.54	0.50	0.20	0.90	0.10
20	0.54	0.55	0.30	0.80	0.07
30	0.54	0.53	0.37	0.73	0.05
40	0.55	0.55	0.40	0.70	0.05
50	0.54	0.54	0.40	0.70	0.05
60	0.54	0.53	0.42	0.68	0.04
70	0.54	0.54	0.44	0.67	0.04
80	0.54	0.54	0.45	0.65	0.03
90	0.54	0.54	0.43	0.64	0.03
100	0.54	0.54	0.46	0.63	0.03
125	0.54	0.55	0.47	0.62	0.02
150	0.54	0.54	0.48	0.60	0.02

(b) Summary of the accuracy value distributions for certain sample sizes.

Table F.38: Results as a function of variable test set sizes with a fixed classifier. To reduce the dimensionality of the feature space a **PCA** was performed and 500 **components** were retained. Following, a **Random Forest** was trained with default parameters. ($n_{\text{estimators}} = 100$)

test sample size	$\geq$ **accuracy**								
n	50	55	60	65	70	75	80	85	90
10	0.12	0.12	0.01	0.01	0.00	0.00	0.00	0.00	0.00
20	0.21	0.03	0.00	0.00	0.00	0.00	0.00	0.00	0.00
30	0.27	0.04	0.00	0.00	0.00	0.00	0.00	0.00	0.00
40	0.28	0.00	0.00	0.00	0.00	0.00	0.00	0.00	0.00
50	0.30	0.01	0.00	0.00	0.00	0.00	0.00	0.00	0.00
60	0.33	0.00	0.00	0.00	0.00	0.00	0.00	0.00	0.00
70	0.35	0.00	0.00	0.00	0.00	0.00	0.00	0.00	0.00
80	0.37	0.00	0.00	0.00	0.00	0.00	0.00	0.00	0.00
90	0.31	0.00	0.00	0.00	0.00	0.00	0.00	0.00	0.00
100	0.37	0.00	0.00	0.00	0.00	0.00	0.00	0.00	0.00
125	0.52	0.00	0.00	0.00	0.00	0.00	0.00	0.00	0.00
150	0.40	0.00	0.00	0.00	0.00	0.00	0.00	0.00	0.00

(a) Probabilities to yield an accuracy exceeding a certain threshold related to the test sample size.

test sample size n	**mean**	**median**	**min**	**max**	**std**
10	0.50	0.50	0.30	0.80	0.05
20	0.50	0.50	0.35	0.65	0.04
30	0.50	0.50	0.40	0.60	0.03
40	0.50	0.50	0.42	0.57	0.03
50	0.50	0.50	0.42	0.56	0.02
60	0.50	0.50	0.43	0.57	0.02
70	0.50	0.50	0.44	0.56	0.02
80	0.50	0.50	0.44	0.55	0.02
90	0.50	0.50	0.46	0.54	0.02
100	0.50	0.50	0.45	0.55	0.01
125	0.50	0.50	0.47	0.54	0.01
150	0.50	0.50	0.47	0.53	0.01

(b) Summary of the accuracy value distributions for certain sample sizes.

Table F.39: Results as a function of variable test set sizes with a fixed classifier. To reduce the dimensionality of the feature space a **PCA** was performed and 500 **components** were retained. Following, an **SVM** with an **RBF kernel** was trained with default parameters. ($C = 1.0$; $\gamma = {}^{1}/{n_{\text{feature}}}$)

test sample size: n	$\geq$ **accuracy** 50	55	60	65	70	75	80	85	90
10	0.62	0.62	0.49	0.36	0.15	0.15	0.04	0.04	0.01
20	0.75	0.56	0.41	0.23	0.11	0.05	0.01	0.01	0.00
30	0.80	0.70	0.43	0.28	0.06	0.03	0.00	0.00	0.00
40	0.85	0.66	0.43	0.16	0.04	0.01	0.00	0.00	0.00
50	0.91	0.76	0.52	0.19	0.03	0.01	0.00	0.00	0.00
60	0.93	0.72	0.40	0.13	0.02	0.00	0.00	0.00	0.00
70	0.95	0.80	0.39	0.15	0.01	0.00	0.00	0.00	0.00
80	0.97	0.77	0.41	0.07	0.01	0.00	0.00	0.00	0.00
90	0.98	0.85	0.42	0.09	0.00	0.00	0.00	0.00	0.00
100	0.98	0.84	0.46	0.07	0.00	0.00	0.00	0.00	0.00
125	1.00	0.91	0.42	0.05	0.00	0.00	0.00	0.00	0.00
150	1.00	0.94	0.41	0.02	0.00	0.00	0.00	0.00	0.00

(a) Probabilities to yield an accuracy exceeding a certain threshold related to the test sample size.

test sample size: n	**mean**	**median**	**min**	**max**	**std**
10	0.60	0.60	0.10	1.00	0.15
20	0.59	0.60	0.25	0.95	0.11
30	0.59	0.60	0.33	0.83	0.09
40	0.59	0.60	0.38	0.80	0.07
50	0.60	0.60	0.38	0.78	0.06
60	0.59	0.58	0.42	0.78	0.06
70	0.59	0.59	0.41	0.77	0.05
80	0.59	0.59	0.42	0.74	0.05
90	0.59	0.60	0.44	0.73	0.04
100	0.59	0.60	0.47	0.73	0.04
125	0.59	0.59	0.49	0.70	0.03
150	0.59	0.59	0.51	0.68	0.03

(b) Summary of the accuracy value distributions for certain sample sizes.

Table F.40: Results as a function of variable test set sizes with a fixed classifier. To reduce the dimensionality of the feature space a **PCA** was performed and 500 **components** were retained. Following, a **linear SVM** was trained with default parameters. ($C = 1.0$)

		≥ **accuracy**								
	n	50	55	60	65	70	75	80	85	90
**test sample size**	10	0.82	0.82	0.71	0.62	0.35	0.35	0.13	0.13	0.03
	20	0.94	0.85	0.74	0.54	0.34	0.17	0.06	0.05	0.00
	30	0.98	0.95	0.80	0.66	0.34	0.21	0.04	0.01	0.00
	40	0.99	0.95	0.88	0.64	0.34	0.12	0.02	0.01	0.00
	50	1.00	0.98	0.91	0.70	0.31	0.13	0.01	0.00	0.00
	60	1.00	1.00	0.93	0.70	0.31	0.07	0.01	0.00	0.00
	70	1.00	0.99	0.94	0.75	0.33	0.08	0.01	0.00	0.00
	80	1.00	1.00	0.97	0.74	0.31	0.05	0.00	0.00	0.00
	90	1.00	1.00	0.98	0.80	0.31	0.04	0.00	0.00	0.00
	100	1.00	1.00	0.98	0.78	0.30	0.03	0.00	0.00	0.00
	125	1.00	1.00	0.99	0.84	0.31	0.02	0.00	0.00	0.00
	150	1.00	1.00	1.00	0.90	0.23	0.01	0.00	0.00	0.00

(a) Probabilities to yield an accuracy exceeding a certain threshold related to the test sample size.

	n	**mean**	**median**	**min**	**max**	**std**
**test sample size**	10	0.69	0.70	0.20	1.00	0.15
	20	0.68	0.70	0.35	0.95	0.10
	30	0.68	0.70	0.40	0.90	0.08
	40	0.68	0.68	0.45	0.95	0.07
	50	0.68	0.68	0.50	0.84	0.06
	60	0.68	0.68	0.53	0.83	0.05
	70	0.68	0.69	0.51	0.83	0.05
	80	0.68	0.69	0.55	0.81	0.04
	90	0.68	0.69	0.57	0.83	0.04
	100	0.68	0.68	0.57	0.80	0.04
	125	0.68	0.68	0.56	0.78	0.03
	150	0.68	0.69	0.59	0.76	0.03

(b) Summary of the accuracy value distributions for certain sample sizes.

Table F.41: Results as a function of variable test set sizes with a fixed classifier. To reduce the dimensionality of the feature space a **PCA** was performed and 500 **components** were retained. For **feature selection** an ANOVA was computed inside the the pipeline and the top 10 **features** were taken based on the ANOVA F-values. Following, a **Random Forest** was trained with default parameters. ($n_{\text{estimators}} = 100$)

test sample size n	≥ accuracy 50	55	60	65	70	75	80	85	90
10	0.36	0.36	0.11	0.07	0.01	0.01	0.00	0.00	0.00
20	0.47	0.22	0.07	0.01	0.00	0.00	0.00	0.00	0.00
30	0.54	0.30	0.03	0.01	0.00	0.00	0.00	0.00	0.00
40	0.59	0.19	0.03	0.00	0.00	0.00	0.00	0.00	0.00
50	0.64	0.26	0.02	0.00	0.00	0.00	0.00	0.00	0.00
60	0.68	0.15	0.01	0.00	0.00	0.00	0.00	0.00	0.00
70	0.69	0.19	0.01	0.00	0.00	0.00	0.00	0.00	0.00
80	0.72	0.13	0.00	0.00	0.00	0.00	0.00	0.00	0.00
90	0.75	0.16	0.00	0.00	0.00	0.00	0.00	0.00	0.00
100	0.76	0.10	0.00	0.00	0.00	0.00	0.00	0.00	0.00
125	0.86	0.07	0.00	0.00	0.00	0.00	0.00	0.00	0.00
150	0.86	0.07	0.00	0.00	0.00	0.00	0.00	0.00	0.00

(a) Probabilities to yield an accuracy exceeding a certain threshold related to the test sample size.

test sample size n	**mean**	**median**	**min**	**max**	**std**
10	0.52	0.50	0.20	0.90	0.09
20	0.52	0.50	0.35	0.75	0.07
30	0.52	0.53	0.37	0.67	0.05
40	0.52	0.53	0.40	0.68	0.05
50	0.52	0.52	0.38	0.64	0.04
60	0.52	0.52	0.40	0.65	0.04
70	0.52	0.53	0.41	0.64	0.03
80	0.52	0.53	0.44	0.61	0.03
90	0.52	0.52	0.43	0.59	0.03
100	0.52	0.52	0.45	0.61	0.02
125	0.52	0.52	0.45	0.58	0.02
150	0.52	0.52	0.45	0.57	0.02

(b) Summary of the accuracy value distributions for certain sample sizes.

Table F.42: Results as a function of variable test set sizes with a fixed classifier. To reduce the dimensionality of the feature space a **PCA** was performed and 500 **components** were retained. For **feature selection** an ANOVA was computed inside the the pipeline and the top 10 **features** were taken based on the ANOVA F-values. Following, an **SVM** with an **RBF kernel** was trained with default parameters. ($C = 1.0$; $\gamma = {}^{1}/{n_{\text{feature}}}$)

test sample size	n	≥ accuracy 50	55	60	65	70	75	80	85	90
	10	0.74	0.74	0.61	0.50	0.24	0.24	0.07	0.07	0.01
	20	0.87	0.75	0.59	0.35	0.20	0.09	0.03	0.03	0.00
	30	0.93	0.86	0.63	0.43	0.17	0.09	0.01	0.01	0.00
	40	0.95	0.86	0.70	0.37	0.16	0.03	0.01	0.00	0.00
	50	0.98	0.93	0.76	0.45	0.14	0.04	0.00	0.00	0.00
	60	0.99	0.93	0.72	0.39	0.11	0.01	0.00	0.00	0.00
	70	1.00	0.97	0.74	0.39	0.08	0.01	0.00	0.00	0.00
	80	1.00	0.98	0.81	0.36	0.07	0.00	0.00	0.00	0.00
	90	1.00	0.98	0.81	0.42	0.06	0.01	0.00	0.00	0.00
	100	1.00	0.98	0.84	0.37	0.04	0.00	0.00	0.00	0.00
	125	1.00	1.00	0.88	0.32	0.03	0.00	0.00	0.00	0.00
	150	1.00	1.00	0.92	0.35	0.01	0.00	0.00	0.00	0.00

(a) Probabilities to yield an accuracy exceeding a certain threshold related to the test sample size.

test sample size	n	**mean**	**median**	**min**	**max**	**std**
	10	0.64	0.65	0.10	1.00	0.15
	20	0.64	0.65	0.30	0.95	0.10
	30	0.64	0.63	0.30	0.93	0.08
	40	0.64	0.65	0.40	0.85	0.07
	50	0.64	0.64	0.48	0.82	0.06
	60	0.64	0.63	0.47	0.82	0.06
	70	0.64	0.64	0.49	0.77	0.05
	80	0.64	0.64	0.51	0.78	0.04
	90	0.64	0.64	0.47	0.77	0.04
	100	0.64	0.64	0.52	0.75	0.04
	125	0.64	0.64	0.54	0.73	0.03
	150	0.64	0.64	0.55	0.73	0.03

(b) Summary of the accuracy value distributions for certain sample sizes.

Table F.43: Results as a function of variable test set sizes with a fixed classifier. To reduce the dimensionality of the feature space a **PCA** was performed and 500 **components** were retained. For **feature selection** an ANOVA was computed inside the the pipeline and the top 10 **features** were taken based on the ANOVA F-values. Following, a **linear SVM** was trained with default parameters. ($C = 1.0$)

	n	≥ **accuracy** 50	55	60	65	70	75	80	85	90
**test sample size**	10	0.79	0.79	0.66	0.54	0.29	0.29	0.10	0.10	0.02
	20	0.93	0.83	0.73	0.48	0.28	0.12	0.05	0.04	0.00
	30	0.97	0.94	0.76	0.60	0.27	0.16	0.03	0.01	0.00
	40	0.99	0.94	0.83	0.55	0.24	0.06	0.01	0.00	0.00
	50	1.00	0.98	0.88	0.63	0.25	0.09	0.01	0.00	0.00
	60	1.00	0.97	0.88	0.59	0.23	0.04	0.00	0.00	0.00
	70	1.00	0.99	0.87	0.62	0.18	0.03	0.00	0.00	0.00
	80	1.00	0.99	0.91	0.60	0.18	0.02	0.00	0.00	0.00
	90	1.00	1.00	0.95	0.68	0.20	0.02	0.00	0.00	0.00
	100	1.00	1.00	0.97	0.63	0.15	0.01	0.00	0.00	0.00
	125	1.00	1.00	0.99	0.69	0.13	0.00	0.00	0.00	0.00
	150	1.00	1.00	1.00	0.75	0.08	0.00	0.00	0.00	0.00

(a) Probabilities to yield an accuracy exceeding a certain threshold related to the test sample size.

	n	**mean**	**median**	**min**	**max**	**std**
**test sample size**	10	0.66	0.70	0.20	1.00	0.15
	20	0.67	0.65	0.35	0.95	0.10
	30	0.67	0.67	0.43	0.93	0.08
	40	0.67	0.68	0.45	0.85	0.06
	50	0.67	0.66	0.48	0.86	0.06
	60	0.67	0.67	0.48	0.82	0.05
	70	0.66	0.67	0.50	0.84	0.05
	80	0.67	0.66	0.51	0.80	0.05
	90	0.67	0.67	0.51	0.81	0.04
	100	0.67	0.67	0.56	0.82	0.04
	125	0.67	0.66	0.57	0.77	0.03
	150	0.67	0.67	0.59	0.74	0.03

(b) Summary of the accuracy value distributions for certain sample sizes.

Table F.44: Results as a function of variable test set sizes with a fixed classifier. To reduce the dimensionality of the feature space a **PCA** was performed and 500 **components** were retained. For **feature selection** an ANOVA was computed inside the the pipeline and the top 100 **features** were taken based on the ANOVA F-values. Following, a **Random Forest** was trained with default parameters. ($n_{\text{estimators}} = 100$)

test sample size	≥ accuracy								
n	50	55	60	65	70	75	80	85	90
10	0.37	0.37	0.08	0.07	0.01	0.01	0.00	0.00	0.00
20	0.53	0.19	0.04	0.01	0.00	0.00	0.00	0.00	0.00
30	0.65	0.35	0.03	0.01	0.00	0.00	0.00	0.00	0.00
40	0.72	0.19	0.02	0.00	0.00	0.00	0.00	0.00	0.00
50	0.74	0.27	0.01	0.00	0.00	0.00	0.00	0.00	0.00
60	0.82	0.18	0.01	0.00	0.00	0.00	0.00	0.00	0.00
70	0.84	0.25	0.00	0.00	0.00	0.00	0.00	0.00	0.00
80	0.89	0.17	0.00	0.00	0.00	0.00	0.00	0.00	0.00
90	0.93	0.24	0.00	0.00	0.00	0.00	0.00	0.00	0.00
100	0.93	0.13	0.00	0.00	0.00	0.00	0.00	0.00	0.00
125	0.98	0.14	0.00	0.00	0.00	0.00	0.00	0.00	0.00
150	0.99	0.10	0.00	0.00	0.00	0.00	0.00	0.00	0.00

(a) Probabilities to yield an accuracy exceeding a certain threshold related to the test sample size.

test sample size n	**mean**	**median**	**min**	**max**	**std**
10	0.54	0.50	0.30	0.90	0.07
20	0.53	0.55	0.40	0.70	0.05
30	0.53	0.53	0.40	0.67	0.04
40	0.53	0.53	0.45	0.65	0.03
50	0.53	0.54	0.44	0.66	0.03
60	0.53	0.53	0.47	0.65	0.03
70	0.53	0.53	0.46	0.61	0.03
80	0.53	0.54	0.47	0.61	0.02
90	0.54	0.53	0.47	0.61	0.02
100	0.53	0.53	0.47	0.61	0.02
125	0.53	0.53	0.48	0.60	0.02
150	0.53	0.53	0.49	0.57	0.01

(b) Summary of the accuracy value distributions for certain sample sizes.

Table F.45: Results as a function of variable test set sizes with a fixed classifier. To reduce the dimensionality of the feature space a **PCA** was performed and 500 **components** were retained. For **feature selection** an ANOVA was computed inside the the pipeline and the top 100 **features** were taken based on the ANOVA F-values. Following, an **SVM** with an **RBF kernel** was trained with default parameters. ($C = 1.0$; $\gamma = {}^{1}/{n_{\text{feature}}}$)

	n	≥ **accuracy** 50	55	60	65	70	75	80	85	90
**test sample size**	10	0.64	0.64	0.51	0.39	0.15	0.15	0.04	0.04	0.00
	20	0.78	0.64	0.47	0.26	0.12	0.05	0.01	0.01	0.00
	30	0.86	0.75	0.48	0.32	0.09	0.04	0.01	0.00	0.00
	40	0.89	0.72	0.53	0.22	0.06	0.01	0.00	0.00	0.00
	50	0.93	0.81	0.53	0.23	0.05	0.01	0.00	0.00	0.00
	60	0.94	0.77	0.45	0.15	0.03	0.00	0.00	0.00	0.00
	70	0.97	0.85	0.47	0.18	0.02	0.00	0.00	0.00	0.00
	80	0.98	0.83	0.52	0.14	0.01	0.00	0.00	0.00	0.00
	90	0.98	0.88	0.53	0.13	0.01	0.00	0.00	0.00	0.00
	100	0.99	0.88	0.50	0.11	0.01	0.00	0.00	0.00	0.00
	125	1.00	0.95	0.53	0.07	0.00	0.00	0.00	0.00	0.00
	150	1.00	0.97	0.53	0.05	0.00	0.00	0.00	0.00	0.00

(a) Probabilities to yield an accuracy exceeding a certain threshold related to the test sample size.

	n	**mean**	**median**	**min**	**max**	**std**
**test sample size**	10	0.60	0.60	0.10	1.00	0.15
	20	0.61	0.60	0.30	0.90	0.10
	30	0.61	0.60	0.30	0.90	0.09
	40	0.60	0.60	0.38	0.82	0.07
	50	0.60	0.60	0.40	0.78	0.06
	60	0.60	0.60	0.43	0.77	0.06
	70	0.60	0.60	0.44	0.76	0.05
	80	0.60	0.60	0.45	0.74	0.05
	90	0.60	0.60	0.47	0.74	0.04
	100	0.60	0.60	0.48	0.73	0.04
	125	0.60	0.60	0.50	0.71	0.03
	150	0.60	0.60	0.51	0.69	0.03

(b) Summary of the accuracy value distributions for certain sample sizes.

Table F.46: Results as a function of variable test set sizes with a fixed classifier. To reduce the dimensionality of the feature space a **PCA** was performed and 500 **components** were retained. For **feature selection** an ANOVA was computed inside the the pipeline and the top 100 **features** were taken based on the ANOVA F-values. Following, a **linear SVM** was trained with default parameters. ($C = 1.0$)

test sample size: n	≥ accuracy: 50	55	60	65	70	75	80	85	90
10	0.67	0.67	0.55	0.42	0.19	0.19	0.04	0.04	0.00
20	0.83	0.66	0.49	0.27	0.12	0.05	0.01	0.01	0.00
30	0.88	0.77	0.52	0.35	0.11	0.04	0.01	0.00	0.00
40	0.92	0.78	0.58	0.24	0.07	0.01	0.00	0.00	0.00
50	0.95	0.85	0.61	0.28	0.05	0.02	0.00	0.00	0.00
60	0.96	0.83	0.54	0.21	0.04	0.00	0.00	0.00	0.00
70	0.97	0.88	0.52	0.22	0.02	0.00	0.00	0.00	0.00
80	0.98	0.87	0.60	0.17	0.02	0.00	0.00	0.00	0.00
90	0.99	0.94	0.58	0.17	0.01	0.00	0.00	0.00	0.00
100	1.00	0.93	0.63	0.16	0.01	0.00	0.00	0.00	0.00
125	1.00	0.98	0.65	0.13	0.00	0.00	0.00	0.00	0.00
150	1.00	0.99	0.69	0.10	0.00	0.00	0.00	0.00	0.00

(a) Probabilities to yield an accuracy exceeding a certain threshold related to the test sample size.

test sample size: n	**mean**	**median**	**min**	**max**	**std**
10	0.61	0.60	0.20	1.00	0.15
20	0.61	0.60	0.30	0.90	0.10
30	0.61	0.60	0.37	0.87	0.08
40	0.61	0.62	0.35	0.82	0.07
50	0.61	0.62	0.42	0.86	0.06
60	0.61	0.62	0.45	0.78	0.06
70	0.61	0.61	0.43	0.77	0.05
80	0.61	0.61	0.47	0.75	0.05
90	0.61	0.61	0.47	0.74	0.04
100	0.61	0.61	0.50	0.72	0.04
125	0.61	0.62	0.51	0.73	0.03
150	0.61	0.61	0.51	0.69	0.03

(b) Summary of the accuracy value distributions for certain sample sizes.

Table F.47: Results as a function of variable test set sizes with a fixed classifier. To reduce the dimensionality of the feature space a **PCA** was performed and **all components** were retained. Following, a **Random Forest** was trained with default parameters. ($n_{\text{estimators}} = 100$)

		≥ accuracy								
	n	50	55	60	65	70	75	80	85	90
**test sample size**	10	0.17	0.17	0.03	0.01	0.00	0.00	0.00	0.00	0.00
	20	0.22	0.05	0.01	0.00	0.00	0.00	0.00	0.00	0.00
	30	0.19	0.07	0.00	0.00	0.00	0.00	0.00	0.00	0.00
	40	0.20	0.03	0.00	0.00	0.00	0.00	0.00	0.00	0.00
	50	0.17	0.02	0.00	0.00	0.00	0.00	0.00	0.00	0.00
	60	0.15	0.01	0.00	0.00	0.00	0.00	0.00	0.00	0.00
	70	0.14	0.01	0.00	0.00	0.00	0.00	0.00	0.00	0.00
	80	0.12	0.00	0.00	0.00	0.00	0.00	0.00	0.00	0.00
	90	0.12	0.00	0.00	0.00	0.00	0.00	0.00	0.00	0.00
	100	0.10	0.00	0.00	0.00	0.00	0.00	0.00	0.00	0.00
	125	0.10	0.00	0.00	0.00	0.00	0.00	0.00	0.00	0.00
	150	0.04	0.00	0.00	0.00	0.00	0.00	0.00	0.00	0.00

(a) Probabilities to yield an accuracy exceeding a certain threshold related to the test sample size.

	n	**mean**	**median**	**min**	**max**	**std**
**test sample size**	10	0.47	0.50	0.20	0.80	0.09
	20	0.47	0.50	0.25	0.65	0.07
	30	0.47	0.47	0.33	0.67	0.05
	40	0.48	0.47	0.35	0.65	0.05
	50	0.48	0.48	0.36	0.60	0.04
	60	0.47	0.47	0.35	0.58	0.03
	70	0.47	0.47	0.39	0.56	0.03
	80	0.47	0.47	0.38	0.56	0.03
	90	0.47	0.48	0.39	0.56	0.03
	100	0.47	0.47	0.40	0.56	0.03
	125	0.47	0.48	0.40	0.56	0.02
	150	0.47	0.47	0.41	0.53	0.02

(b) Summary of the accuracy value distributions for certain sample sizes.

Table F.48: Results as a function of variable test set sizes with a fixed classifier. To reduce the dimensionality of the feature space a **PCA** was performed and **all components** were retained. Following, an **SVM** with an **RBF kernel** was trained with default parameters. ($C = 1.0$; $\gamma = {}^{1}/_{n_{\text{feature}}}$)

		≥ **accuracy**								
	n	50	55	60	65	70	75	80	85	90
**test sample size**	10	0.79	0.79	0.66	0.54	0.27	0.27	0.09	0.09	0.02
	20	0.90	0.79	0.64	0.43	0.24	0.10	0.04	0.03	0.00
	30	0.95	0.91	0.68	0.54	0.23	0.12	0.02	0.01	0.00
	40	0.98	0.91	0.78	0.48	0.21	0.06	0.01	0.00	0.00
	50	0.99	0.95	0.82	0.57	0.19	0.06	0.00	0.00	0.00
	60	0.99	0.97	0.82	0.49	0.17	0.02	0.00	0.00	0.00
	70	0.99	0.98	0.85	0.56	0.16	0.03	0.00	0.00	0.00
	80	1.00	0.99	0.86	0.51	0.14	0.01	0.00	0.00	0.00
	90	1.00	0.99	0.90	0.55	0.11	0.01	0.00	0.00	0.00
	100	1.00	1.00	0.92	0.51	0.11	0.00	0.00	0.00	0.00
	125	1.00	1.00	0.95	0.55	0.07	0.00	0.00	0.00	0.00
	150	1.00	1.00	0.98	0.56	0.04	0.00	0.00	0.00	0.00

(a) Probabilities to yield an accuracy exceeding a certain threshold related to the test sample size.

	n	**mean**	**median**	**min**	**max**	**std**
**test sample size**	10	0.66	0.70	0.20	1.00	0.15
	20	0.65	0.65	0.35	0.95	0.10
	30	0.66	0.67	0.40	0.90	0.08
	40	0.66	0.65	0.42	0.85	0.07
	50	0.66	0.66	0.46	0.84	0.06
	60	0.66	0.65	0.48	0.83	0.05
	70	0.66	0.66	0.50	0.80	0.05
	80	0.66	0.66	0.50	0.81	0.05
	90	0.66	0.66	0.52	0.78	0.04
	100	0.66	0.66	0.53	0.76	0.04
	125	0.66	0.66	0.57	0.76	0.03
	150	0.66	0.65	0.56	0.75	0.03

(b) Summary of the accuracy value distributions for certain sample sizes.

Table F.49: Results as a function of variable test set sizes with a fixed classifier. To reduce the dimensionality of the feature space a **PCA** was performed and **all components** were retained. Following, a **linear SVM** was trained with default parameters. ($C = 1.0$)

test sample size n	≥ accuracy 50	55	60	65	70	75	80	85	90
10	0.75	0.75	0.62	0.50	0.24	0.24	0.09	0.09	0.02
20	0.88	0.76	0.62	0.42	0.24	0.11	0.05	0.04	0.00
30	0.94	0.87	0.67	0.50	0.20	0.10	0.01	0.00	0.00
40	0.97	0.89	0.75	0.43	0.20	0.05	0.01	0.00	0.00
50	0.98	0.94	0.78	0.44	0.13	0.04	0.00	0.00	0.00
60	0.99	0.94	0.76	0.43	0.14	0.02	0.00	0.00	0.00
70	1.00	0.97	0.77	0.45	0.11	0.02	0.00	0.00	0.00
80	1.00	0.97	0.86	0.42	0.09	0.01	0.00	0.00	0.00
90	1.00	0.98	0.84	0.49	0.09	0.01	0.00	0.00	0.00
100	1.00	0.99	0.89	0.43	0.07	0.00	0.00	0.00	0.00
125	1.00	1.00	0.93	0.42	0.05	0.00	0.00	0.00	0.00
150	1.00	1.00	0.95	0.41	0.02	0.00	0.00	0.00	0.00

(a) Probabilities to yield an accuracy exceeding a certain threshold related to the test sample size.

test sample size n	**mean**	**median**	**min**	**max**	**std**
10	0.65	0.60	0.20	1.00	0.15
20	0.65	0.65	0.30	0.90	0.11
30	0.65	0.63	0.40	0.90	0.08
40	0.65	0.65	0.40	0.90	0.07
50	0.64	0.64	0.42	0.86	0.06
60	0.65	0.65	0.43	0.82	0.06
70	0.64	0.64	0.49	0.80	0.05
80	0.65	0.65	0.46	0.78	0.05
90	0.65	0.64	0.51	0.79	0.04
100	0.65	0.65	0.52	0.76	0.04
125	0.65	0.65	0.55	0.75	0.03
150	0.64	0.65	0.55	0.73	0.03

(b) Summary of the accuracy value distributions for certain sample sizes.

Table F.50: Results as a function of variable test set sizes with a fixed classifier. To reduce the dimensionality of the feature space a **PCA** was performed and **all components** were retained. For **feature selection** an ANOVA was computed inside the the pipeline and the top 10 **features** were taken based on the ANOVA F-values. Following, a **Random Forest** was trained with default parameters. ($n_{\text{estimators}} = 100$)

		≥ accuracy								
	n	50	55	60	65	70	75	80	85	90
test sample size	10	0.34	0.34	0.12	0.09	0.01	0.01	0.00	0.00	0.00
	20	0.46	0.17	0.05	0.01	0.00	0.00	0.00	0.00	0.00
	30	0.52	0.26	0.03	0.01	0.00	0.00	0.00	0.00	0.00
	40	0.57	0.17	0.01	0.00	0.00	0.00	0.00	0.00	0.00
	50	0.59	0.20	0.01	0.00	0.00	0.00	0.00	0.00	0.00
	60	0.63	0.13	0.00	0.00	0.00	0.00	0.00	0.00	0.00
	70	0.66	0.19	0.00	0.00	0.00	0.00	0.00	0.00	0.00
	80	0.69	0.11	0.00	0.00	0.00	0.00	0.00	0.00	0.00
	90	0.71	0.13	0.00	0.00	0.00	0.00	0.00	0.00	0.00
	100	0.70	0.06	0.00	0.00	0.00	0.00	0.00	0.00	0.00
	125	0.82	0.07	0.00	0.00	0.00	0.00	0.00	0.00	0.00
	150	0.83	0.04	0.00	0.00	0.00	0.00	0.00	0.00	0.00

(a) Probabilities to yield an accuracy exceeding a certain threshold related to the test sample size.

	n	**mean**	**median**	**min**	**max**	**std**
test sample size	10	0.52	0.50	0.20	0.80	0.09
	20	0.52	0.50	0.30	0.75	0.06
	30	0.52	0.53	0.37	0.67	0.05
	40	0.52	0.53	0.38	0.68	0.04
	50	0.52	0.52	0.40	0.64	0.04
	60	0.52	0.52	0.42	0.65	0.03
	70	0.52	0.51	0.43	0.63	0.03
	80	0.52	0.53	0.44	0.61	0.03
	90	0.52	0.52	0.44	0.60	0.03
	100	0.52	0.52	0.45	0.59	0.02
	125	0.52	0.52	0.46	0.59	0.02
	150	0.52	0.52	0.47	0.59	0.02

(b) Summary of the accuracy value distributions for certain sample sizes.

Table F.51: Results as a function of variable test set sizes with a fixed classifier. To reduce the dimensionality of the feature space a **PCA** was performed and **all components** were retained. For **feature selection** an ANOVA was computed inside the the pipeline and the top 10 **features** were taken based on the ANOVA F-values. Following, an **SVM** with an **RBF kernel** was trained with default parameters. ($C = 1.0$; $\gamma = {}^{1}/{n_{\text{feature}}}$)

	n	≥ accuracy 50	55	60	65	70	75	80	85	90
**test sample size**	10	0.11	0.11	0.07	0.03	0.01	0.01	0.00	0.00	0.00
	20	0.06	0.02	0.01	0.00	0.00	0.00	0.00	0.00	0.00
	30	0.03	0.01	0.00	0.00	0.00	0.00	0.00	0.00	0.00
	40	0.02	0.00	0.00	0.00	0.00	0.00	0.00	0.00	0.00
	50	0.00	0.00	0.00	0.00	0.00	0.00	0.00	0.00	0.00
	60	0.00	0.00	0.00	0.00	0.00	0.00	0.00	0.00	0.00
	70	0.01	0.00	0.00	0.00	0.00	0.00	0.00	0.00	0.00
	80	0.00	0.00	0.00	0.00	0.00	0.00	0.00	0.00	0.00
	90	0.00	0.00	0.00	0.00	0.00	0.00	0.00	0.00	0.00
	100	0.00	0.00	0.00	0.00	0.00	0.00	0.00	0.00	0.00
	125	0.00	0.00	0.00	0.00	0.00	0.00	0.00	0.00	0.00
	150	0.00	0.00	0.00	0.00	0.00	0.00	0.00	0.00	0.00

(a) Probabilities to yield an accuracy exceeding a certain threshold related to the test sample size.

	n	**mean**	**median**	**min**	**max**	**std**
**test sample size**	10	0.36	0.40	0.00	0.90	0.15
	20	0.36	0.35	0.05	0.70	0.10
	30	0.35	0.37	0.07	0.60	0.08
	40	0.36	0.35	0.15	0.60	0.07
	50	0.35	0.36	0.16	0.58	0.06
	60	0.36	0.35	0.18	0.52	0.06
	70	0.36	0.36	0.23	0.56	0.05
	80	0.36	0.36	0.24	0.51	0.04
	90	0.36	0.36	0.22	0.49	0.04
	100	0.36	0.36	0.23	0.48	0.04
	125	0.36	0.35	0.25	0.45	0.03
	150	0.36	0.36	0.25	0.45	0.03

(b) Summary of the accuracy value distributions for certain sample sizes.

Table F.52: Results as a function of variable test set sizes with a fixed classifier. To reduce the dimensionality of the feature space a **PCA** was performed and **all components** were retained. For **feature selection** an ANOVA was computed inside the the pipeline and the top 10 **features** were taken based on the ANOVA F-values. Following, a **linear SVM** was trained with default parameters. ($C = 1.0$)

test sample size n	≥ accuracy 50	55	60	65	70	75	80	85	90
10	0.70	0.70	0.58	0.47	0.22	0.22	0.06	0.06	0.01
20	0.83	0.71	0.57	0.36	0.21	0.10	0.04	0.03	0.00
30	0.92	0.85	0.59	0.41	0.16	0.08	0.01	0.00	0.00
40	0.95	0.84	0.67	0.33	0.13	0.03	0.00	0.00	0.00
50	0.97	0.90	0.68	0.36	0.11	0.03	0.00	0.00	0.00
60	0.98	0.89	0.65	0.31	0.08	0.01	0.00	0.00	0.00
70	0.99	0.94	0.66	0.33	0.05	0.00	0.00	0.00	0.00
80	1.00	0.95	0.73	0.29	0.05	0.00	0.00	0.00	0.00
90	1.00	0.98	0.72	0.32	0.03	0.00	0.00	0.00	0.00
100	1.00	0.98	0.79	0.27	0.03	0.00	0.00	0.00	0.00
125	1.00	0.99	0.81	0.24	0.01	0.00	0.00	0.00	0.00
150	1.00	1.00	0.85	0.22	0.01	0.00	0.00	0.00	0.00

(a) Probabilities to yield an accuracy exceeding a certain threshold related to the test sample size.

test sample size n	**mean**	**median**	**min**	**max**	**std**
10	0.63	0.60	0.10	1.00	0.15
20	0.63	0.65	0.30	0.95	0.11
30	0.63	0.63	0.37	0.87	0.08
40	0.63	0.62	0.42	0.85	0.07
50	0.63	0.62	0.44	0.86	0.06
60	0.63	0.63	0.47	0.82	0.06
70	0.63	0.63	0.46	0.83	0.05
80	0.63	0.64	0.49	0.79	0.05
90	0.63	0.63	0.51	0.74	0.04
100	0.63	0.63	0.52	0.75	0.04
125	0.63	0.63	0.54	0.73	0.03
150	0.63	0.63	0.53	0.73	0.03

(b) Summary of the accuracy value distributions for certain sample sizes.

Table F.53: Results as a function of variable test set sizes with a fixed classifier. To reduce the dimensionality of the feature space a **PCA** was performed and **all components** were retained. For **feature selection** an ANOVA was computed inside the the pipeline and the top 100 **features** were taken based on the ANOVA F-values. Following, a **Random Forest** was trained with default parameters. ($n_{\text{estimators}} = 100$)

		≥ accuracy								
	n	50	55	60	65	70	75	80	85	90
test sample size	10	0.51	0.51	0.20	0.16	0.03	0.03	0.00	0.00	0.00
	20	0.71	0.40	0.16	0.05	0.01	0.00	0.00	0.00	0.00
	30	0.79	0.55	0.13	0.04	0.00	0.00	0.00	0.00	0.00
	40	0.86	0.45	0.12	0.01	0.00	0.00	0.00	0.00	0.00
	50	0.90	0.59	0.10	0.01	0.00	0.00	0.00	0.00	0.00
	60	0.93	0.51	0.08	0.00	0.00	0.00	0.00	0.00	0.00
	70	0.96	0.63	0.06	0.00	0.00	0.00	0.00	0.00	0.00
	80	0.98	0.57	0.06	0.00	0.00	0.00	0.00	0.00	0.00
	90	0.97	0.64	0.04	0.00	0.00	0.00	0.00	0.00	0.00
	100	0.99	0.56	0.04	0.00	0.00	0.00	0.00	0.00	0.00
	125	1.00	0.64	0.02	0.00	0.00	0.00	0.00	0.00	0.00
	150	1.00	0.71	0.01	0.00	0.00	0.00	0.00	0.00	0.00

(a) Probabilities to yield an accuracy exceeding a certain threshold related to the test sample size.

	n	**mean**	**median**	**min**	**max**	**std**
test sample size	10	0.56	0.60	0.20	0.80	0.10
	20	0.56	0.55	0.35	0.80	0.07
	30	0.56	0.57	0.37	0.73	0.05
	40	0.56	0.55	0.40	0.68	0.04
	50	0.56	0.56	0.44	0.66	0.04
	60	0.56	0.57	0.45	0.67	0.03
	70	0.56	0.56	0.46	0.64	0.03
	80	0.56	0.56	0.47	0.64	0.03
	90	0.56	0.56	0.46	0.64	0.03
	100	0.56	0.56	0.48	0.64	0.03
	125	0.56	0.56	0.48	0.64	0.02
	150	0.56	0.56	0.50	0.61	0.02

(b) Summary of the accuracy value distributions for certain sample sizes.

Table F.54: Results as a function of variable test set sizes with a fixed classifier. To reduce the dimensionality of the feature space a **PCA** was performed and **all components** were retained. For **feature selection** an ANOVA was computed inside the the pipeline and the top 100 **features** were taken based on the ANOVA F-values. Following, an **SVM** with an **RBF kernel** was trained with default parameters. ($C = 1.0$; $\gamma = {}^{1}/_{n_{\text{feature}}}$)

test sample size n	≥ accuracy 50	55	60	65	70	75	80	85	90
10	0.70	0.70	0.55	0.44	0.20	0.20	0.06	0.06	0.01
20	0.83	0.67	0.52	0.31	0.17	0.07	0.01	0.01	0.00
30	0.88	0.81	0.55	0.37	0.13	0.07	0.01	0.00	0.00
40	0.93	0.78	0.61	0.27	0.10	0.03	0.00	0.00	0.00
50	0.96	0.87	0.66	0.34	0.08	0.02	0.00	0.00	0.00
60	0.98	0.88	0.62	0.26	0.05	0.01	0.00	0.00	0.00
70	0.98	0.92	0.62	0.28	0.04	0.00	0.00	0.00	0.00
80	1.00	0.94	0.68	0.24	0.04	0.00	0.00	0.00	0.00
90	0.99	0.95	0.69	0.27	0.02	0.00	0.00	0.00	0.00
100	1.00	0.96	0.73	0.19	0.02	0.00	0.00	0.00	0.00
125	1.00	0.98	0.75	0.19	0.01	0.00	0.00	0.00	0.00
150	1.00	0.99	0.79	0.16	0.01	0.00	0.00	0.00	0.00

(a) Probabilities to yield an accuracy exceeding a certain threshold related to the test sample size.

test sample size n	**mean**	**median**	**min**	**max**	**std**
10	0.63	0.60	0.20	1.00	0.14
20	0.62	0.60	0.25	0.90	0.10
30	0.62	0.63	0.37	0.87	0.09
40	0.62	0.62	0.35	0.88	0.07
50	0.62	0.62	0.44	0.82	0.06
60	0.62	0.63	0.45	0.83	0.05
70	0.62	0.63	0.41	0.77	0.05
80	0.62	0.62	0.47	0.76	0.05
90	0.62	0.62	0.48	0.76	0.04
100	0.62	0.62	0.50	0.75	0.04
125	0.62	0.62	0.50	0.72	0.03
150	0.62	0.62	0.53	0.73	0.03

(b) Summary of the accuracy value distributions for certain sample sizes.

Table F.55: Results as a function of variable test set sizes with a fixed classifier. To reduce the dimensionality of the feature space a **PCA** was performed and **all components** were retained. For **feature selection** an ANOVA was computed inside the the pipeline and the top 100 **features** were taken based on the ANOVA F-values. Following, a **linear SVM** was trained with default parameters. ($C = 1.0$)

		≥ **accuracy**								
	n	50	55	60	65	70	75	80	85	90
**test sample size**	10	0.57	0.57	0.44	0.31	0.12	0.12	0.04	0.04	0.01
	20	0.69	0.50	0.35	0.17	0.09	0.03	0.01	0.01	0.00
	30	0.77	0.65	0.35	0.21	0.06	0.03	0.00	0.00	0.00
	40	0.82	0.61	0.38	0.12	0.03	0.01	0.00	0.00	0.00
	50	0.87	0.68	0.39	0.13	0.02	0.00	0.00	0.00	0.00
	60	0.89	0.65	0.33	0.08	0.02	0.00	0.00	0.00	0.00
	70	0.92	0.72	0.32	0.09	0.01	0.00	0.00	0.00	0.00
	80	0.94	0.65	0.29	0.04	0.00	0.00	0.00	0.00	0.00
	90	0.95	0.75	0.29	0.06	0.00	0.00	0.00	0.00	0.00
	100	0.96	0.73	0.29	0.03	0.00	0.00	0.00	0.00	0.00
	125	0.99	0.84	0.28	0.01	0.00	0.00	0.00	0.00	0.00
	150	1.00	0.85	0.23	0.01	0.00	0.00	0.00	0.00	0.00

(a) Probabilities to yield an accuracy exceeding a certain threshold related to the test sample size.

	n	**mean**	**median**	**min**	**max**	**std**
**test sample size**	10	0.58	0.60	0.10	1.00	0.15
	20	0.58	0.57	0.25	0.90	0.11
	30	0.58	0.57	0.27	0.87	0.09
	40	0.58	0.57	0.35	0.80	0.07
	50	0.58	0.58	0.40	0.76	0.06
	60	0.58	0.58	0.43	0.77	0.06
	70	0.58	0.59	0.41	0.76	0.05
	80	0.58	0.57	0.44	0.71	0.05
	90	0.58	0.58	0.42	0.71	0.04
	100	0.58	0.58	0.44	0.70	0.04
	125	0.58	0.58	0.48	0.68	0.03
	150	0.58	0.58	0.48	0.69	0.03

(b) Summary of the accuracy value distributions for certain sample sizes.

Table F.56: Results as a function of variable test set sizes with a fixed classifier. To reduce the dimensionality of the feature space a **PCA** was performed and **all components** were retained. For **feature selection** an ANOVA was computed inside the the pipeline and the top 1,000 **features** were taken based on the ANOVA F-values. Following, a **Random Forest** was trained with default parameters. ($n_{\text{estimators}} = 100$)

test sample size	n	≥ accuracy 50	55	60	65	70	75	80	85	90
	10	0.53	0.53	0.33	0.23	0.06	0.06	0.01	0.01	0.00
	20	0.71	0.47	0.28	0.09	0.02	0.01	0.00	0.00	0.00
	30	0.73	0.56	0.23	0.09	0.01	0.00	0.00	0.00	0.00
	40	0.80	0.47	0.22	0.04	0.01	0.00	0.00	0.00	0.00
	50	0.83	0.57	0.20	0.03	0.00	0.00	0.00	0.00	0.00
	60	0.88	0.51	0.16	0.02	0.00	0.00	0.00	0.00	0.00
	70	0.90	0.61	0.13	0.01	0.00	0.00	0.00	0.00	0.00
	80	0.94	0.54	0.13	0.00	0.00	0.00	0.00	0.00	0.00
	90	0.94	0.59	0.09	0.01	0.00	0.00	0.00	0.00	0.00
	100	0.96	0.55	0.09	0.00	0.00	0.00	0.00	0.00	0.00
	125	0.98	0.60	0.07	0.00	0.00	0.00	0.00	0.00	0.00
	150	0.99	0.66	0.03	0.00	0.00	0.00	0.00	0.00	0.00

(a) Probabilities to yield an accuracy exceeding a certain threshold related to the test sample size.

test sample size	n	**mean**	**median**	**min**	**max**	**std**
	10	0.56	0.60	0.20	1.00	0.13
	20	0.57	0.55	0.30	0.85	0.09
	30	0.56	0.57	0.33	0.77	0.07
	40	0.56	0.55	0.40	0.78	0.06
	50	0.56	0.56	0.38	0.72	0.05
	60	0.56	0.57	0.42	0.70	0.05
	70	0.56	0.56	0.43	0.69	0.04
	80	0.56	0.56	0.44	0.68	0.04
	90	0.56	0.56	0.44	0.67	0.03
	100	0.56	0.56	0.45	0.65	0.03
	125	0.56	0.56	0.48	0.65	0.03
	150	0.56	0.56	0.49	0.63	0.02

(b) Summary of the accuracy value distributions for certain sample sizes.

Table F.57: Results as a function of variable test set sizes with a fixed classifier. To reduce the dimensionality of the feature space a **PCA** was performed and **all components** were retained. For **feature selection** an ANOVA was computed inside the the pipeline and the top 1,000 **features** were taken based on the ANOVA F-values. Following, an **SVM** with an **RBF kernel** was trained with default parameters. ($C = 1.0$; $\gamma = {}^{1}/_{n_{\text{feature}}}$)

test sample size: n	≥ accuracy: 50	55	60	65	70	75	80	85	90
10	0.77	0.77	0.67	0.54	0.27	0.27	0.10	0.10	0.02
20	0.91	0.81	0.68	0.44	0.27	0.12	0.04	0.04	0.00
30	0.93	0.87	0.67	0.50	0.21	0.12	0.03	0.01	0.00
40	0.97	0.90	0.78	0.47	0.21	0.07	0.01	0.00	0.00
50	0.99	0.95	0.82	0.53	0.19	0.05	0.00	0.00	0.00
60	0.99	0.96	0.81	0.49	0.17	0.03	0.00	0.00	0.00
70	1.00	0.98	0.83	0.56	0.18	0.04	0.00	0.00	0.00
80	1.00	0.98	0.89	0.49	0.15	0.02	0.00	0.00	0.00
90	1.00	1.00	0.88	0.56	0.11	0.02	0.00	0.00	0.00
100	1.00	0.99	0.92	0.55	0.11	0.01	0.00	0.00	0.00
125	1.00	1.00	0.96	0.55	0.09	0.00	0.00	0.00	0.00
150	1.00	1.00	0.98	0.59	0.05	0.00	0.00	0.00	0.00

(a) Probabilities to yield an accuracy exceeding a certain threshold related to the test sample size.

test sample size: n	**mean**	**median**	**min**	**max**	**std**
10	0.66	0.70	0.20	1.00	0.15
20	0.66	0.65	0.35	0.90	0.10
30	0.65	0.63	0.43	0.97	0.08
40	0.66	0.65	0.45	0.90	0.07
50	0.65	0.66	0.46	0.86	0.06
60	0.66	0.65	0.47	0.82	0.05
70	0.66	0.66	0.50	0.81	0.05
80	0.66	0.65	0.49	0.80	0.05
90	0.65	0.66	0.53	0.81	0.04
100	0.66	0.66	0.53	0.80	0.04
125	0.66	0.66	0.56	0.78	0.03
150	0.66	0.66	0.57	0.74	0.03

(b) Summary of the accuracy value distributions for certain sample sizes.

Table F.58: Results as a function of variable test set sizes with a fixed classifier. To reduce the dimensionality of the feature space a **PCA** was performed and **all components** were retained. For **feature selection** an ANOVA was computed inside the the pipeline and the top 1,000 **features** were taken based on the ANOVA F-values. Following, a **linear SVM** was trained with default parameters. ($C = 1.0$)
